# Feasibility of Prospectively Comparing Opioid Analgesia With Opioid-Free Analgesia After Outpatient General Surgery: A Pilot Randomized Clinical Trial

**DOI:** 10.1001/jamanetworkopen.2022.21430

**Published:** 2022-07-18

**Authors:** Uyen Do, Charbel El-Kefraoui, Makena Pook, Saba Balvardi, Natasha Barone, Philip Nguyen-Powanda, Lawrence Lee, Gabriele Baldini, Liane S. Feldman, Julio F. Fiore, and the McGill Better Opioid Prescribing Collaboration, Mohsen Alhashemi, Alen Antoun, Jeffrey S. Barkun, Krista M. Brecht, Prosanto K. Chaudhury, Dan Deckelbaum, Elise Di Lena, Sinziana Dumitra, Hiba Elhaj, Paola Fata, David Fleiszer, Gerald M. Fried, Jeremy Grushka, Pepa Kaneva, Kosar Khwaja, Maxime Lapointe-Gagner, Katherine M. McKendy, Ari N. Meguerditchian, Sarkis H. Meterissian, Haley Montgomery, Fateme Rajabiyazdi, Nadia Safa, Nawar Touma, Francine Tremblay

**Affiliations:** 1 Steinberg-Bernstein Centre for Minimally Invasive Surgery and Innovation, McGill University Health Centre, Montreal, Quebec, Canada; 2 Division of Experimental Surgery, McGill University, Montreal, Quebec, Canada; 3 Division of General Surgery, Department of Surgery, McGill University, Montreal, Quebec, Canada; 4 Faculty of Medicine and Health Sciences, McGill University, Montreal, Quebec, Canada; 5 Centre for Outcomes Research and Evaluation (CORE), Research Institute of the McGill University Health Centre, Montreal, Quebec, Canada; 6 Department of Anesthesia, McGill University, Montreal, Quebec, Canada; 7 Alan Edwards Pain Management Unit, Montreal, Quebec, Canada

## Abstract

**Importance:**

The overprescription of opioids to surgical patients is recognized as an important factor contributing to the opioid crisis. However, the value of prescribing opioid analgesia (OA) vs opioid-free analgesia (OFA) after postoperative discharge remains uncertain.

**Objective:**

To investigate the feasibility of conducting a full-scale randomized clinical trial (RCT) to assess the comparative effectiveness of OA vs OFA after outpatient general surgery.

**Design, Setting, and Participants:**

This parallel, 2-group, assessor-blind, pragmatic pilot RCT was conducted from January 29 to September 3, 2020 (last follow-up on October 2, 2020). at 2 university-affiliated hospitals in Montreal, Quebec, Canada. Participants were adult patients (aged ≥18 years) undergoing outpatient abdominal (ie, cholecystectomy, appendectomy, or hernia repair) or breast (ie, partial or total mastectomy) general surgical procedures. Exclusion criteria were contraindications to drugs used in the trial, preoperative opioid use, conditions that could affect assessment of outcomes, and intraoperative or early complications requiring hospitalization.

**Interventions:**

Patients were randomized 1:1 to receive OA (around-the-clock nonopioids and opioids for breakthrough pain) or OFA (around-the-clock nonopioids with increasing doses and/or addition of nonopioid medications for breakthrough pain) after postoperative discharge.

**Main Outcomes and Measures:**

Main outcomes were a priori RCT feasibility criteria (ie, rates of surgeon agreement, patient eligibility, patient consent, treatment adherence, loss to follow-up, and missing follow-up data). Secondary outcomes included pain intensity and interference, analgesic intake, 30-day unplanned health care use, and adverse events. Between-group comparison of outcomes followed the intention-to-treat principle.

**Results:**

A total of 15 surgeons were approached; all (100%; 95% CI, 78%-100%) agreed to have patients recruited and adhered to the study procedures. Rates of patient eligibility and consent were 73% (95% CI, 66%-78%) and 57% (95% CI, 49%-65%), respectively. Seventy-six patients were randomized (39 [51%] to OA and 37 [49%] to OFA) and included in the intention-to-treat analysis (mean [SD] age, 55.5 [14.5] years; 50 [66%] female); 40 (53%) underwent abdominal surgery, and 36 (47%) underwent breast surgery. Seventy-five patients (99%; 95% CI, 93%-100%) adhered to the allocated treatment; 1 patient randomly assigned to OFA received an opioid prescription. Seventeen patients (44%) randomly assigned to OA consumed opioids after discharge. Seventy-three patients (96%; 95% CI, 89%-99%) completed the 30-day follow-up. The rate of missing questionnaires was 37 of 3724 (1%; 95% CI, 0.7%-1.4%). All the a priori RCT feasibility criteria were fulfilled.

**Conclusions and Relevance:**

The findings of this pilot RCT support the feasibility of conducting a robust, full-scale RCT to inform evidence-based prescribing of analgesia after outpatient general surgery.

**Trial Registration:**

ClinicalTrials.gov Identifier: NCT04254679

## Key Points

**Question:**

Is it feasible to conduct a randomized clinical trial (RCT) comparing opioid analgesia with opioid-free analgesia after outpatient general surgery?

**Findings:**

In this pilot RCT, 76 patients were randomized 1:1 to receive opioid or opioid-free analgesia after postoperative discharge. The trial proposed was well accepted by the stakeholders involved, including patients and clinicians, and all a priori feasibility criteria were fulfilled.

**Meaning:**

These findings support the feasibility of an RCT to prospectively compare opioid analgesia with opioid-free analgesia after hospital discharge following outpatient general surgery.

## Teaser

This pilot randomized clinical trial (RCT) assessed the feasibility of a full-scale RCT to compare the outcomes of opioid analgesia vs opioid-free analgesia prescribed to adult patients after undergoing outpatient general surgery.

## Introduction

The overprescription of opioids by surgeons is recognized as a contributing factor to the current crisis of opioid misuse and addiction in North America.^1,2^ Patients undergoing outpatient general surgery (ie, with planned same-day discharge) are particularly at risk because they are often prescribed opioid tablets to be taken at home during the first postoperative days.^3,4^ Under current prescribing patterns, up to 6% of opioid-naive patients undergoing common outpatient general surgical procedures (ie, cholecystectomies, hernia repairs, and mastectomies) become persistent opioid users postoperatively.^5,6,7^ Those who do not become persistent users may also contribute to the opioid crisis by diverting unused tablets for nonmedical use by others.^8^ Literature suggests that to prevent opioid-related harms after outpatient general surgery, clinicians may consider prescribing only nonopioid drugs to manage pain after discharge.^9,10^ However, whereas this practice is common in some countries,^3,4^ evidence regarding the comparative effectiveness of opioid analgesia (OA) vs opioid-free analgesia (OFA) remains uncertain. Findings from a scoping review suggest that the number of randomized clinical trials (RCTs) in the field of pain management is limited,^11^ although existing small trials often challenged the value of prescribing opioids for postdischarge analgesia after general surgery.^12,13,14^ Owing to a lack of evidence in this research area, the decision to prescribe opioids largely depends on surgeons’ preference and the health care culture; hence, there is an urgent need for robust RCTs to guide clinical decision-making.

Owing to the complexity inherent in well-designed RCTs, pilot studies are a critical first step to assess acceptability, test logistical needs, optimize design, and inform the capacities required for a full-scale trial.^15^ Undertaking an RCT with OFA raises important practical concerns including surgeon and patient hesitation about pain treatment without opioids, the randomization approach, adherence, and optimal outcome measurement. Thus, the objective of this pilot study was to investigate the feasibility of conducting a full-scale RCT to assess the comparative effectiveness of analgesia regimens including opioids (OA) vs OFA regimens after outpatient general surgery.

## Methods

This pilot RCT was approved by the institutional ethics board of the McGill University Health Centre, and all participants provided written informed consent. The trial protocol (Supplement 1) was registered a priori (NCT04254679). Analyses and reporting followed the Consolidated Standards of Reporting Trials (CONSORT) reporting guideline extension for pilot trials (Supplement 2).^16^

### Study Design and Patients

This was a parallel, 2-group, assessor-blind, pragmatic pilot RCT with participants allocated 1:1 to receive OA or OFA after postoperative discharge. Recruitment of participants occurred between January 29 and September 3, 2020 (the last follow-up for self-reported outcomes was October 2, 2020). The trial was halted from March 15 to June 1, 2020, owing to COVID-19 restrictions. We included adult patients (aged ≥18 years) undergoing outpatient abdominal (ie, appendectomies, cholecystectomies, and hernia repairs) and breast (ie, partial and total mastectomies) general surgical procedures at 2 university-affiliated hospitals in Montreal, Quebec, Canada. All surgeries were performed by fellowship-trained surgeons; the surgeons’ agreement to have patients in the trial was required for inclusion. We excluded patients with contraindications to the drugs used in the trial (ie, substance use disorder, heart failure, allergy, peptic ulcer, bleeding disorders, and kidney or liver impairment),^17,18,19^ those who were taking opioids preoperatively, and those with conditions that could impact outcome assessment (eg, cognitive impairment, inability to understand English or French, and limited access to a telephone or computer). Patients were excluded postoperatively if they had intraoperative or early complications requiring a hospital stay.

### Randomization and Blinding

The random allocation sequence was generated electronically (via Sealed Envelope^20^) by an external researcher not involved in the trial and was uploaded to REDCap.^21^ Permuted blocks of varying sizes (2, 4, or 6) were used, and randomization was stratified by abdominal surgery vs breast surgery. There was no stratification by center because the trial sites were specialized in either procedure type. Randomizations were conducted by research staff present in the operating room using the project’s REDCap randomization module. Treatment allocations were concealed until patients were deemed ready to be discharged from the operating room to the postanesthesia care unit.

After randomization, patients and surgeons were not blinded to the treatment allocation owing to the pragmatic nature of the trial. The primary surgeon was informed about the randomization result in the operating room after skin closure and was provided a discharge analgesia prescription according to the group assignment. To prevent performance bias during the postanesthesia care unit stay (eg, patients in the OFA group receiving additional analgesia before discharge), the prescription was kept in a sealed opaque envelope until patients were deemed ready to leave the hospital. Outcome assessors (N.B., H.E., and H.M.) were blinded to treatment allocations. Blinding effectiveness was estimated by asking assessors to guess the patient’s group allocation after the last follow-up assessment. Any inadvertent unblinding was reported.

### Interventions

#### OA Group

The discharge prescription in the OA group (standard care) included around-the-clock nonopioid analgesics (acetaminophen and/or nonsteroidal anti-inflammatory drugs) and a supply of opioids to be used as rescue analgesia for breakthrough pain. Given the pragmatic nature of this trial, the specific OA regimen was determined by the patient’s primary surgeon considering the surgical procedure, comorbidities, and patient preference. The OA strategies used at each trial site were guided by the institution’s pain service team and followed Health Canada standards for safety and efficacy.^22^ Examples are described in eFigure 1 in Supplement 2.

#### OFA Group

The discharge prescription in the OFA group included only around-the-clock nonopioid analgesics (acetaminophen alone and/or nonsteroidal anti-inflammatory drugs). In case of breakthrough pain, rescue analgesia was provided by (1) increasing doses of nonopioid analgesics, (2) adding nonopioid drugs that were not included in the initial regimen, or (3) switching drugs according to single-dose efficacy evidence^23^ targeting individual variances in analgesia response.^24^ The regimen prescribed was determined by the patient’s primary surgeon. Suggested OFA strategies, developed with input from the pain service team according to Health Canada standards,^22^ are described in eFigure 2 in Supplement 2.

#### Management of Persistent Pain

In accordance with standard practice at the trial sites, in case of persistent pain despite the available prescription, patients in the OA group were advised to call the surgeon’s office or clinic during working hours (weekdays, 8 am to 4 pm) or visit the hospital emergency department (after hours and weekends) for assessment and potential pain management optimization.

Because OFA was new to the institutions in the trial, a strategy was implemented to ensure that patients received adequate pain management during the trial. At hospital discharge, patients receiving OFA had a backup prescription of opioids faxed to a pharmacy close to their residence. To prevent patients from filling out this prescription “just in case,” they were not informed about the availability of the prescription unless they reported persistent pain via a study hotline available 24 hours a day, 7 days a week (ie, a dedicated mobile telephone kept with study staff). When this line was called, patients were informed about the availability of the opioid prescription.

#### Other Aspects of Perioperative Care

Surgical techniques and in-hospital anesthesia and analgesia interventions were left to the discretion of the surgeons and anesthesiologists to best reflect routine practice. The use of nonpharmacological pain interventions (eg, ice compress, relaxation) were permitted and recorded.

### Measurement Strategy

#### Patient, Surgical, and Perioperative Care Characteristics

Details about patient, surgical, and perioperative care characteristics were obtained from electronic medical records. Preoperatively, we also collected self-reported data on pain catastrophizing (Pain Catastrophizing Scale^25,26^), potential for opioid misuse (Screener and Opioid Assessment for Patients With Pain^27^), preferred treatment group, and expectations for treatment effectiveness. Details are provided in eTable 1 in Supplement 2.

#### Feasibility Outcomes

As a pilot study, this trial primarily focused on a priori feasibility outcomes. A full-scale RCT would be deemed feasible if, during the study period, (1) at least 90% of the surgeons who conducted the procedures of interest agreed to have patients randomly assigned to treatment and adhered to the agreement (ie, they did not change their minds), (2) at least 70% of patients undergoing the procedures of interest were eligible to be randomly assigned, (3) at least 50% of eligible patients agreed to participate in the study and were randomly assigned, (4) at least 80% of the patients who were randomly assigned adhered to their allocated treatment (ie, they did not receive an opioid prescription if randomly assigned to receive OFA), (5) at least 80% of the randomly assigned patients completed an outcome assessment at 30 days, and (6) among patients who completed outcome assessments, the proportion of missing data (ie, nonresponse to questionnaires or to specific questionnaire items) was 10% or less.

#### Clinical Outcomes

Clinical outcomes were assessed secondarily to inform the measurement strategy and sample size requirements for a future full-scale RCT (ie, by estimating variability [SDs] and prevalence of key clinical outcomes). Our outcome measurement strategy included the Brief Pain Inventory–Short Form (pain intensity and pain interference domains),^28^ time to stopping pain medication,^29^ Patient-Reported Outcomes Measurement Information System 29 profile (physical function, anxiety, depression, fatigue, sleep disturbance, social roles and activities, pain intensity, and pain interference domains),^30^ perioperative Opioid-Related Symptom Distress Scale,^31^ Prescription Opioid Misuse Index,^32^ 30-day complications,^33,34^ 30-day unplanned health care use (emergency department visits, unplanned clinic visits, and/or hospital readmissions), 30-day drug-related adverse events (identified using the perioperative Opioid-Related Symptom Distress Scale, medical records, or MedDRA-classified self-report^35^), and prolonged opioid use (3-month follow-up). Details are given in eTable 2 in Supplement 2.

### Data Collection and Follow-up Procedures

Patient-reported outcomes were collected preoperatively (baseline), on postoperative days 1 to 7, and at postoperative weeks 2 (day 14), 3 (day 21), and 4 (day 28). Data collection was via electronic questionnaires distributed using REDCap and completed on a smartphone, tablet, or personal computer. Electronic data were transmitted directly to a REDCap database and verified by blinded assessors (N.B., H.E., and H.M.). Patients also had the option to complete questionnaires by telephone with a blinded assessor. Information regarding 30-day complications and unplanned health care use was obtained via self-report with electronic medical record confirmation. Information regarding opioid dispensing was monitored for 3 months on a provincewide medical database (Dossier Santé Québec). Treatment adherence was monitored (via the REDCap questionnaires or telephone) by unblinded study staff not involved in outcome assessment.

### Sample Size

In accordance with previous recommendations that at least 70 measured participants are required to estimate SDs with enough precision for future sample size calculations,^36^ we aimed to recruit and obtain outcome data from 80 patients (40 per group), allowing for an attrition rate of approximately 15%. This sample size is also consistent with recommendations regarding the minimal number of participants required to identify feasibility issues.^37^

### Statistical Analysis

Feasibility outcomes were estimated using descriptive statistics with 95% CIs. Between-group comparison of outcomes followed the intention-to-treat principle and focused on descriptive statistics and exploratory effect estimates with 95% CIs. Because this was a feasibility trial, no inferential statistics targeting statistical significance were analyzed.^16^ To inform the generalizability of our results, we compared the characteristics of randomly assigned patients with those of patients who did not consent to randomization. All analyses were performed using Stata, version 16 (StataCorp LLC).

## Results

**Figure 1.**
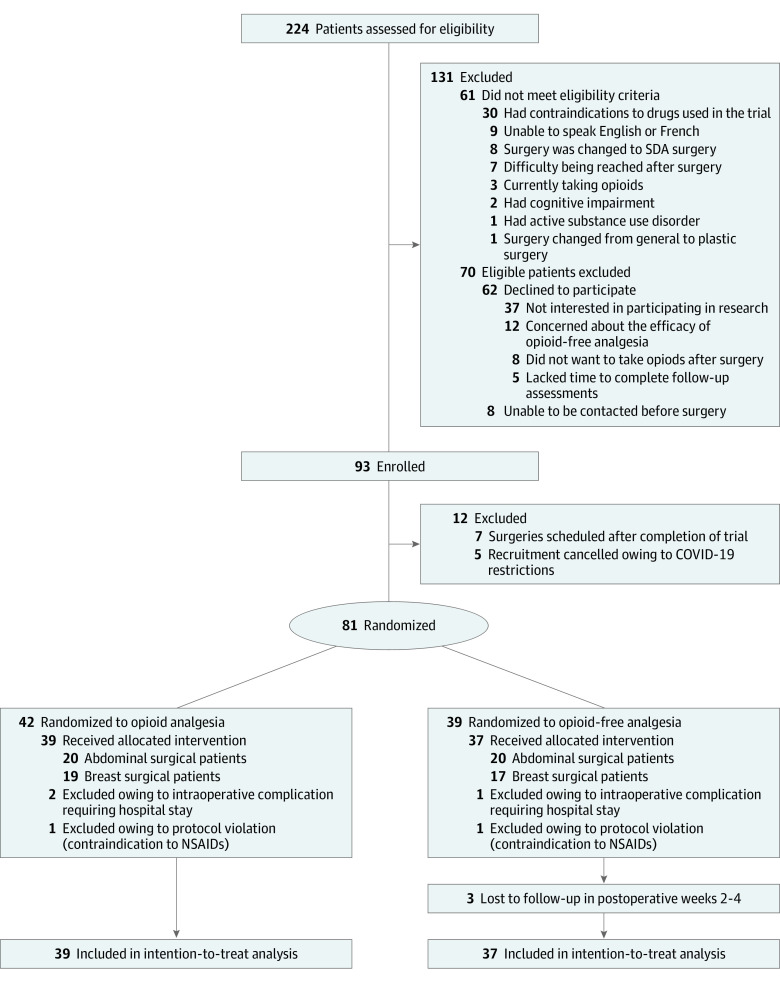
CONSORT Diagram NSAIDs indicates nonsteroidal anti-inflammatory drugs; SDA, same-day admission.

All 15 of the surgeons (100%; 95% CI, 78%-100%) who conducted the eligible surgeries during the study period agreed to have patients recruited and adhered to the study procedures. The CONSORT diagram is shown in Figure 1. Rates of patient eligibility and consent to randomization were 73% (95% CI, 66%-78%) and 57% (95% CI, 49%-65%), respectively. Among the 70 eligible patients who were excluded from enrollment, the most common reasons for declining to participate were unwillingness to take part in research while receiving care (42 [60%]) and having preconceptions about the use of opioids for postoperative analgesia (20 [29%]) (Figure 1). More specifically, 12 patients (17%) had concerns about the efficacy of OFA and 8 patients (11%) did not want to take opioids after surgery. The characteristics of randomly assigned patients vs those who did not consent were similar (eTable 3 in Supplement 2). Five patients were excluded after randomization (3 developed complications requiring a hospital stay and 2 had a contraindication to nonsteroidal anti-inflammatory drugs identified after randomization), but no patients in either group withdrew owing to lack of treatment efficacy or adverse effects. Overall, 76 patients (39 [51%] in the OA group and 37 [49%] in the OFA group) were included in the intention-to-treat analysis. Participants’ baseline and operative characteristics are reported in Table 1. The mean (SD) age of the sample was 55.5 (14.5) years (range, 21-85 years), and 50 patients (66%) were female. Forty patients (53%) underwent abdominal surgery (19 [48%] laparoscopic), and 36 (47%) underwent breast surgery (15 [42%] with sentinel node biopsy and 7 [19%] with axillary node dissection). Seventy-five patients (99%; 95% CI, 93%-100%) adhered to treatment allocation, and 73 (96%; 95% CI, 89%-99%) completed the 30-day follow-up. Thirty-seven of 3724 questionnaires (1%; 95% CI, 0.7%-1.4%) were missing, and 33 of 32 256 questionnaire items (0.1%; 95% CI, 0.1%-0.1%) were missing. Based on these findings, all the a priori feasibility criteria set for this pilot trial were fulfilled (Table 2). Outcome assessors correctly guessed the group allocation for 37 patients (49%) (no more than expected by chance), which is consistent with blinding effectiveness (eTable 4 in Supplement 2).

**Table 1.** Patient Baseline and Operative Characteristics

Characteristic	Patients^a^		
	Total (N = 76)	Opioid analgesia (n = 39)	Opioid-free analgesia (n = 37)
Age, y			
Mean (SD)	55.5 (14.5)	54.3 (15.1)	56.8 (14.0)
≥75	5 (7)	2 (5)	3 (8)
Sex			
Female	50 (66)	24 (61)	26 (70)
Male	26 (34)	15 (38)	11 (30)
BMI			
Mean (SD)	27.6 (7.0)^b^	26.4 (4.7)^c^	28.8 (8.7)^d^
≥30.0	18 (25)^b^	7 (19)^c^	11 (31)^d^
Physical status^e^			
I	15 (20)	6 (15)	9 (24)
II	53 (70)	25 (64)	28 (76)
III	8 (10)	2 (5)	6 (16)
Risk of opioid abuse score^f^			
Mean (SD)	1.9 (1.6)	2.0 (1.6)	1.8 (1.7)
Score ≥4	9 (12)	4 (10)	5 (14)
Pain catastrophizing score, mean (SD)^g^	13.6 (10.7)	13.5 (10.7)	13.6 (11.0)
Employment			
Employed, including self-employed	44 (58)	24 (62)	20 (54)
Retired	20 (26)	9 (23)	11 (30)
Homemaker	2 (3)	2 (5)	0
Student	1 (1)	0	1 (3)
Unemployed	5 (7)	1 (3)	4 (10)
Unable to work, receiving disability pension	4 (5)	3 (8)	1 (3)
Current smoker	13 (18)^c^	8 (21)^d^	5 (14)^d^
Prerandomization treatment group preference^h^			
Unsure or no preference	28 (37)	13 (33)	15 (41)
Opioid medication group	11 (15)	7 (18)	4 (10)
Opioid-free medication group	37 (49)	19 (49)	18 (49)
Prerandomization perceptions of opioid analgesia^i^			
Very effective	37 (49)	17 (44)	20 (54)
Somewhat effective	8 (11)	4 (10)	4 (10)
Not effective	1 (1)	0	1 (3)
No specific expectation	30 (39)	18 (46)	12 (33)
Prerandomization perceptions of opioid-free analgesia^j^			
Very effective	23 (30)	8 (21)	15 (41)
Somewhat effective	28 (37)	17 (44)	11 (30)
Not effective	2 (3)	0	2 (5)
No specific expectation	23 (30)	14 (35)	9 (24)
Abdominal surgery	40 (53)	20 (51)	20 (54)
Laparoscopic appendectomy	1 (1)	0	1 (3)
Laparoscopic cholecystectomy	9 (12)	3 (8)	6 (16)
Laparoscopic inguinal hernia repair	9 (12)	8 (21)^k^	1 (3)
Open inguinal hernia repair	17 (22)	8 (21)	9 (24)
Open umbilical hernia repair	3 (4)	1 (3)	2 (6)
Open incisional hernia repair	1 (1)	0	1 (3)
Breast surgery	36 (47)	19 (49)	17 (46)
Partial mastectomy	14 (18)	4 (10)	10 (27)
Partial mastectomy with sentinel node biopsy	11 (14)	7 (18)	4 (11)
Partial mastectomy with axillary node dissection	6 (8)	4 (10)	2 (6)
Partial mastectomy with sentinel node biopsy and reconstruction	1 (1)	0	1 (3)
Total mastectomy with sentinel node biopsy	2 (3)	2 (5)	0
Total mastectomy with sentinel node biopsy and reconstruction	1 (1)	1 (3)	0
Total mastectomy with axillary node dissection and reconstruction	1 (1)	1 (3)	0
Received intraoperative regional analgesia	57 (75)	31 (79)	26 (70)
Peripheral nerve block	11 (14)	5 (13)	6 (16)
Wound infiltration	57 (75)	31 (79)	26 (70)
Duration of surgery, mean (SD), min	91 (45)	97 (39)	84 (51)
Amount of opioids received in the PACU, mean (SD), MME	21 (18)^c^	18 (14)^d^	25 (21)^d^

Abbreviations: BMI, body mass index (calculated as weight in kilograms divided by height in meters squared); MME, morphine milligram equivalent; PACU, postanesthesia care unit.

^a^ Data are presented as the number (percentage) of patients unless otherwise indicated.

^b^ Data missing for 3 patients.

^c^ Data missing for 2 patients.

^d^ Data missing for 1 patient.

^e^ Based on American Society of Anesthesiology score.

^f^ Assessed by the Screener and Opioid Assessment for Patients With Pain Short Form (total score range, 0-20; score ≥4 indicates a likely high risk of opioid misuse after prescription).^27^

^g^ Assessed by the Pain Catastrophizing Scale (recall period not specific; total score range, 0-52, with higher scores indicating worse pain catastrophizing).^25,26^

^h^ Patients were asked, “What treatment group do you prefer to be in?”

^i^ Patients were asked, “If you are in the group using opioids for pain treatment, what is your expectation of treatment effectiveness?”

^j^ Patients were asked, “If you are in the group not using opioids for pain treatment, what is your expectation of treatment effectiveness?”

^k^ Includes 1 patient who had an umbilical hernia repair during the same procedure.

**Table 2.** Feasibility Outcomes

Feasibility criteria	No./total No. (%) [95% CI]
≥90% Of surgeons agreed to have their patients randomly assigned to treatment and adhered to the agreement	15/15 (100%) [ 78%-100%]
≥70% Of screened patients were eligible to be randomly assigned to treatment	163/224 (73%) [66%-78%]
≥50% Of eligible patients agreed to participate in the study	93/168 (57%) [49%-65%]
≥80% Of patients randomly assigned adhered to their allocated treatment	75/76 (99%) [93%-100%]
≥80% Of patients randomly assigned completed the outcome assessment 30 d after surgery	73/76 (96%) [89%-99%]
≤10% Of data were missing among patients who completed the outcome assessment	
Questionnaires	37/3724 (1%) [0.7%-1.4%]
Questionnaire items	33/32 256 (0.1%) [0.1%-0.1%]

Before randomization, most patients stated a preference for being randomly assigned to the OFA group (37 patients [49%]) or had no preference (28 patients [37%]). Thirty-seven patients (49%) expected that OA would be very effective, and 28 (37%) expected that OFA would be somewhat effective. The OA and OFA regimens prescribed at discharge are described in eTable 5 in Supplement 2. In the OA group, the mean (SD) amount of opioids prescribed was 106 (82) morphine milligram equivalents^38^ (equivalent to approximately 14 pills of oxycodone, 5 mg), 25 patients (64%) filled their opioid prescription, and 17 (44%) reported consuming opioids after discharge. In the OFA group, 8 patients (22%) used the rescue nonopioid analgesia available in their prescription. Only 1 patient (3%) in the OFA group (after an open inguinal hernia repair) filled an opioid prescription after calling the study hotline owing to uncontrolled pain.

**Figure 2.**
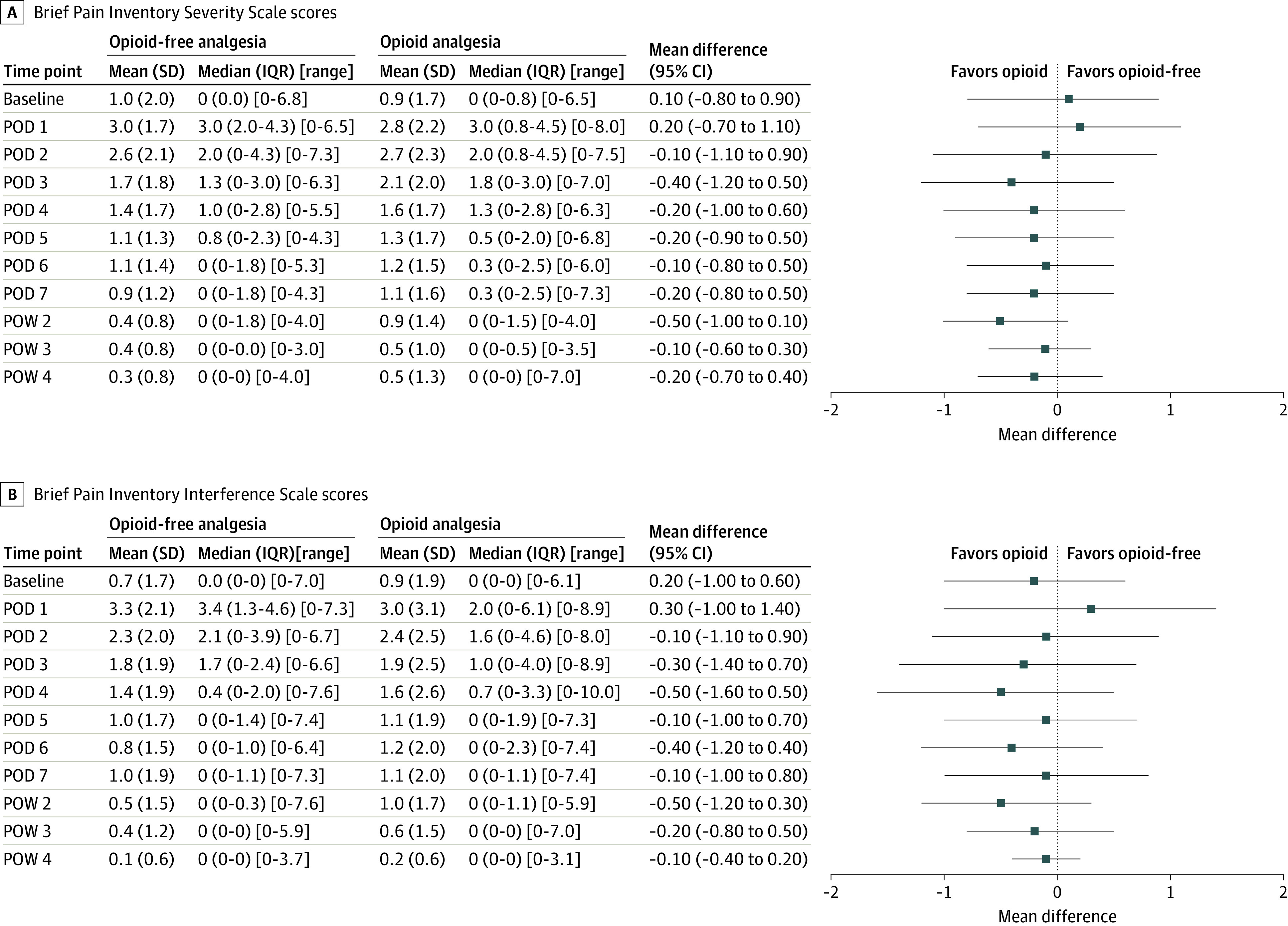
Between-Group Differences in Postoperative Pain Intensity and Interference A, The Brief Pain Inventory Severity Scale is a composite of 4 items with a score range of 0 to 10 (higher scores indicate greater pain severity).^28^ B, The Interference Scale is a composite of 7 items with a score range of 0 to 10 (higher scores indicate a greater degree of pain-related interference with functioning).^28^ Follow-up data were missing for 2 patients in postoperative week (POW) 3 and for 3 patients in POW 4. Squares indicate mean differences, with horizontal lines indicating 95% CIs. Vertical lines represent minimal clinically important differences.^39,40^ POD indicates postoperative day.

Data regarding postoperative pain intensity and interference are reported in Figure 2.^39,40^ Domains for the Assessment of Patient-Reported Outcomes Measurement Information System 29 are reported in eFigure 3 in Supplement 2. Subgroup analyses by surgery type (abdominal and breast) are reported in eFigures 4 to 7 in Supplement 2. Satisfaction with pain management and time to stopping pain medication are reported in Table 3. One patient (1%) was at risk of opioid misuse disorder (Prescription Opioid Misuse Index score, ≥2) at 30 days. During the 3-month follow-up, 3 patients filled new opioid prescriptions (OA group: 1 [3%]; OFA group: 2 [6%]), all owing to a new surgical procedure (revision of breast resection margin).

**Table 3.** Postoperative Outcomes

Outcome	Patients, No. (%)		Between-group difference (95% CI)^a^
	Opioid analgesia (n = 39)	Opioid-free analgesia (n = 37)	
Filled out an opioid prescription^b^			
Postoperative week 1	25 (64)	1 (3)	−61.4 (−78.2 to −44.6)
Postoperative week 2	0	0	0.0 (0.0 to 0.0)
Postoperative week 3	0	0	0.0 (0.0 to 0.0)
Postoperative week 4	0	0	0.0 (0.0 to 0.0)
Postoperative month 2	0	1 (3)	2.7 (−2.5 to 7.9)
Postoperative month 3	1 (3)	2 (6)	2.8 (−6.1 to 11.7)
Time to stopping pain medications, mean (SD), d^c^	9 (9.0)	9 (7.9)	−1.1 (−4.3 to 3.5)
Prescription Opioid Misuse Index score			
Mean (SD)^d^	0.1 (0.4)	0.1 (0.4)	0.0 (−0.21 to 0.13)
Score ≥2	0	1 (3)	2.7 (−2.7 to 8.6)
Satisfied or very satisfied with pain management^e^	37 (95)	34 (92)	−3.0 (−14.2 to 8.2)
Wished to have received a better pain management strategy^f^	6 (15)	6 (16)	0.8 (−17.7 to 16.1)
Postoperative complications at 30 d^g^	6 (15)	2 (5)	−10.0 (−23.4 to 3.4)
Surgical site infection	2 (5)	0	−5.1 (−12.1 to 1.8)
Breast hematoma	1 (3)	0	−2.6 (−7.5 to 2.4)
Urinary retention	1 (3)	0	−2.6 (−7.5 to 2.4)
Neuropathic pain	1 (3)	0	−2.6 (−7.5 to 2.4)
Scrotal ecchymosis	1 (3)	0	−2.6 (−7.5 to 2.4)
Testicular hematoma	0	1 (3)	2.7 (−2.5 to 7.9)
Breast seroma	0	1 (3)	2.7 (−2.5 to 7.9)
Postoperative complication score at 30 d^h^			
I	3 (8)	1 (3)	−5.0 (−14.9 to 4.9)
II	2 (5)	0	−5.1 (−12.1 to 1.8)
IIIa/b	1 (3)	1 (3)	0.1 (−7.1 to 7.3)
Comprehensive Complication Index score at 30 d, median (IQR)^i^	2.6 (7.2)	0.9 (4.5)	1.7 (−1.1 to 4.4)
Unplanned health care use at 30 d			
Any	6 (15)	1 (3)	−12.7 (−25.1 to 0.2)
ED visit	5 (13)	0	−12.8 (−19.8 to −0.7)
Readmission	1 (3)	1 (3)	0.1 (−7.2 to 7.9)
Outpatient clinic visit	2 (5)	0	−5.1 (−12.1 to 1.8)

Abbreviation: ED, emergency department.

^a^ Between-group difference indicates the mean difference for continuous variables and the proportion difference (as a percentage) for dichotomous variables.

^b^ Data collected from Dossier Santé Québec; data for 1 patient from the opioid analgesia group were missing owing to restricted access to the patient’s files.

^c^ Time to the first report of stopping the use of pain medication was calculated based on the first of 2 consecutive reports of “did not use pain medication” from postoperative day 1 to 7. If analgesia intake continued beyond postoperative day 7, patients were asked to recall the last day of pain medication use at postoperative weeks 2, 3, and 4. For patients who were lost to follow-up (3 patients in the opioid-free analgesia group), the last reported dates of medication use were used in the analysis.

^d^ Assessed by the Prescription Opioid Abuse Index^32^ (recall period, 4 weeks; total score range, 0-6; score ≥2 indicates a likely diagnosis of medication misuse disorder).

^e^ At postoperative day 7, patients were asked, “How satisfied are you with the pain treatment you have received after the operation?” Response options were “very dissatisfied,” “dissatisfied,” “satisfied,” or “very satisfied.”

^f^ At postoperative day 7, patients were asked, “Do you wish that your pain was better managed by the health care team?” Response options were “yes” or “no.”

^g^ Data were collected from patients’ clinical medical records.

^h^ Clavien-Dindo classification.^33^

^i^ Score ranges from 0 to 100; higher scores indicate greater severity of complications.^34^

Rates of adverse events identified using the perioperative Opioid-Related Symptom Distress Scale questionnaire are reported in eTable 6 in Supplement 2. Most events were reported within 7 days and included nausea (OA group: 8 patients [21%]; OFA group: 6 [16%]), vomiting (OA group: 3 [8%]; OFA group: 1 [3%]), constipation (OA group: 16 [41%]; OFA group: 12 [32%]), and itching (OA group: 13 [33%]; OA group: 7 [19%]). Other postoperative health issues spontaneously reported by patients included headache (OA group: 4 [10%]; OFA group: 1 [3%]) and diarrhea (OA group: 1 [3%]; OFA group: 2 [5%]) (eTable 7 in Supplement 2). Postoperative complications occurred in 6 patients in the OA group (15%) and 2 patients in the OFA group (5%) (Table 3). Unplanned health care use was required by 6 patients in the OA group (15%) and 1 patient in the OFA group (3%).

## Discussion

Findings from this pilot trial support the feasibility of conducting a full-scale RCT to compare OA with OFA after outpatient general surgery. Overall, the trial proposed was welcomed by all the stakeholders involved (ie, funders, ethics committee, patients, scientists, surgeons, anesthesiologists, and other perioperative care clinicians), supporting recognition of the uncertainty regarding comparative effectiveness of OA vs OFA after postoperative discharge.

The most common barrier to participation among eligible patients was a lack of willingness to take part in research while receiving care (42 [60%]); however, a considerable proportion of patients (20 [29%]) did not consent to randomization because of preconceptions about the use of opioids for postoperative analgesia. Although some patients (12 [17%]) were concerned about the efficacy of OFA, others (8 [11%]) did not want to take opioids given the risk of addiction and adverse effects (Figure 1). This finding supports that recruitment for a full-scale trial may be facilitated by addressing implicit biases and emphasizing the equipoise between the 2 interventions. Of interest, most patients stated a preference for being randomly assigned to the OFA group (49%) or had no preference (37%). Among patients randomly assigned to OA, only 64% filled their prescription and 44% used opioids after discharge. The latter finding corroborates previous literature showing that a considerable number of opioid pills prescribed to surgical patients go unused.^8^ Of note, patients’ option for not taking opioids (even when randomly assigned to receive a prescription) is inherent to a pragmatic trial aiming to assess the value of opioid prescribing in real-world settings.

The prescription of opioids to surgical patients often results from concerns of inadequate pain control after discharge, which may be associated with increased emergency visits and readmissions.^41,42^ Given this concern, trial participants randomized to the OFA group had a study hotline available to report uncontrolled pain and a backup opioid prescription faxed to their pharmacy. During the study period, only 2 patients used the study hotline to report uncontrolled pain. One patient ultimately filled the backup opioid prescription, whereas the other reported improvement after optimizing the dosage of nonopioid drugs (previously taken incorrectly). None of the episodes of unplanned health care use had uncontrolled pain as the chief complaint. However, of note, the overall rates of unplanned health care use tended to be higher among patients randomly assigned to OA (15% vs 3%). Postoperative complications, which were the main causes of emergency department visits and readmissions, also tended to be higher among patients in the OA group (15% vs 5%). Although these findings may have occurred by chance given the study’s small sample size, they warrant further investigation in a full-scale RCT.

### Limitations

This study has limitations. This pilot RCT was not statistically powered to detect differences in outcomes, so any between-group comparison should be interpreted with caution.^16^ Our feasibility findings were obtained in 2 tertiary academic hospitals in Canada and may not be generalizable to other contexts of care. Patient recruitment was interrupted owing to the COVID-19 pandemic; we cannot exclude that widespread social isolation may have affected some aspects of the trial (ie, seeking care for potential complications or adverse events). Randomization of patients in the postanesthesia care unit (immediately before hospital discharge) would have optimized concealment of allocation, but this was considered impractical by surgeons who often write their prescriptions in the operating room after skin closure. Two patients were excluded from the trial after randomization because they had contraindications to nonsteroidal anti-inflammatory drugs not documented in electronic medical records. This indicates that further screening measures are warranted in a full-scale RCT (ie, confirming patient eligibility with the medical team before randomization). An ongoing qualitative study involving perioperative care clinicians and patients who participated in the trial will further elucidate challenges and mitigation strategies in preparation for a future full-scale RCT.

## Conclusions

The overprescription of opioids postoperatively is recognized as a contributing factor to the current opioid crisis. Patients undergoing outpatient general surgery are frequently prescribed opioids after discharge, but the value of this practice remains uncertain. Findings from this pilot trial support the feasibility of conducting a robust, full-scale trial to inform evidence-based analgesia prescribing.

## Notes

Supplement 1.
Trial Protocol

Supplement 2. **eFigure 1.** Postoperative Analgesia Regimens for Opioid Analgesia (OA) Group **eFigure 2.** Postoperative Analgesia Regimens for Opioid-free Analgesia (OFA) Group **eTable 1.** Screening Measures: Constructs Targeted, Corresponding Measures (or Sources of Data), and Description **eTable 2.** Outcome Measures: Constructs Targeted, Corresponding Measures (or Sources of Data), and Description **eTable 3.** Relevant Baseline and Operative Characteristics of Eligible Patients Who Consented and Did Not Consent to Participation **eTable 4.** Comparison Between the Outcome Assessors’ Guesses About Allocation (Opioid vs Opioid-free Analgesia) and Actual Allocation at Postoperative Week 4 After Surgery **eFigure 3.** Between-group Differences in the PROMIS-29 Domains T-scores **eFigure 4.** Subgroup Analysis of Brief Pain Inventory in Patients Undergoing Abdominal Surgery **eFigure 5.** Subgroup Analysis of Brief Pain Inventory in Patients Undergoing Breast Surgery **eFigure 6.** Subgroup Analysis of PROMIS-29 Domains T-scores in Patients Undergoing Abdominal Surgery **eFigure 7.** Subgroup Analysis of PROMIS-29 Domains T-scores in Patients Undergoing Breast Surgery **eTable 5.** Postoperative Pain Management Regimens **eTable 6.** Adverse Events Identified via the Perioperative Opioid-Related Symptom Distress Scale **eTable 7.** Patient-reported Postoperative Health Issues (Classified Using MedDRA)
eReferences

Supplement 3.
Data Sharing Statement
