# Supplementary material for: Feasibility of Prospectively Comparing Opioid Analgesia With Opioid-Free Analgesia After Outpatient General Surgery: A Pilot Randomized Clinical Trial
Source: JAMA Netw Open. 2022 Jul 18;5(7):e2221430. doi: 10.1001/jamanetworkopen.2022.21430 (PMC9294998; doi:10.1001/jamanetworkopen.2022.21430)
Supplement: Supplement 1. — Trial Protocol [file jamanetwopen-e2221430-s001.pdf]

Supplement 1. Study protocol as approved by ethics

Project title: Opioid-free analgesia after outpatient general surgery: A pilot randomized controlled trial

RATIONALE

Canada is in the midst of an epidemic of opioid use and abuse fueled by increased prescriptions by physicians. Overprescription has been implicated as a driving force behind the growing number of overdoses and deaths caused by opioids. Canada has the second highest rate of opioid prescription per-capita in the world after the United States[1]. Physicians wrote on average one opioid prescription for every two Canadians in 2017[2]. In the same year, at least 4100 opioid-related deaths occurred across Canada[3]. This death toll increased to 4460 in 2018, which represents an average of 12 Canadians dying from opioid overdoses every day[3]. The estimated economic cost of opioid misuse in Canada, accounting for health, justice, lost productivity and other direct costs, tops $3.5 billion per year[4]. As a response to this grim statistic, the federal Minister of Health has made combatting the 'opioid crisis' a top priority[5].

Surgery often serves as the initial event for opioid-naïve patients to obtain a prescription for opioids and spiral into misuse and addiction[6,7]. Those undergoing outpatient surgery (i.e., with same day discharge), which represent nearly 80% of all surgeries performed in Canada and the United States[8], are particularly vulnerable as they invariably require some form of analgesia to be taken at home during the first postoperative days. In North America, analgesia for these patients often includes over-the-counter non-opioid drugs [e.g., acetaminophen and/or non-steroid anti-inflammatory drugs (NSAIDs)/Cox-2 inhibitors (COX-2)] and prescription opioid tablets to be taken 'as needed' in case of breakthrough pain. With this current prescription pattern, up to 1-in-10 patients become persistent opioid users postoperatively, i.e., they continue to take the drug for more than three months after surgery[6,9,10]. Those who do not become persistent users may also contribute to the opioid epidemic by diverting unused tablets for nonmedical use by others. A recent systematic review suggests that of all opioid tablets obtained by surgical patients 42% to 71% go unused[11]. In other words, they are prescribed

unnecessarily and become a readily available source for diversion. It is estimated that over 50% of people who abuse opioids obtain the drug via diversion from friends or relatives with unused prescriptions[12]. Although the prescription of opioids after outpatient surgery seems harmless to many, postoperative overprescription is an urgent element of the opioid crisis given how commonly it may contribute to misuse, diversion, addiction and death.

From the perspective of surgeons and other perioperative care clinicians, the answer to the opioid crisis may be preventing opioid prescriptions whenever possible using opioid-free analgesia. In European countries, postoperative discharge prescriptions commonly include only non-opioid drugs while, interestingly, pain-related outcomes (i.e., satisfaction with pain treatment) are often superior to North America[13-15]. Moreover, evidence regarding the benefits of postoperative opioids has largely relied on unimodal, single-dose studies conducted for regulatory purposes under strict experimental conditions[16]. Arguably, a more appropriate approach to guide clinical practice is to examine the impact of postoperative opioids in 'real-world' conditions, where analgesia strategies are often multimodal and pain treatment span several days. Data from a scoping review recently completed by our research group (currently under peer-review for publication) supports that the number of comparative studies in this field is limited, while existing small trials often challenge the value of adding opioids to multimodal analgesia regimens[17-19]. Lack of evidence in this field means that the decision to prescribe opioids after outpatient surgery largely depends on healthcare culture and surgeon preference. Hence, there is an urgent need for robust randomized clinical trials (RCTs) to guide clinical decision-making.

Due to the complexity inherent to well-designed RCTs, pilot studies are critical to assess acceptability, test logistical aspects, optimize design and build the capacities required for a full-scale trial[20]. Undertaking an RCT of opioid-free analgesia raises important practical concerns including: surgeon and patient hesitation about pain treatment without opioids, decision regarding participation under preoperative stress, treatment adherence and optimal measurement strategies. Thus, the overarching objective of the proposed pilot study is to investigate the feasibility of conducting a full-scale, pragmatic RCT aimed to estimate the extent to which analgesia regimens

including opioids (opioid analgesia, OA) impact postoperative outcomes after outpatient general surgery in comparison to regimens that are opioid-free (opioid-free analgesia, OFA). By addressing the prevention of opioid prescription after outpatient surgery, this proposal tackles the first pillar of the New Canadian Drugs and Substances Strategy (CDSS), i.e., preventing problematic drug and substance use supported by a strong evidence base[21].

SPECIFIC RESEARCH OBJECTIVES

PART 1. Main study (Pilot RCT)

1.1. To estimate the proportion of screened patients who meet eligibility criteria.

1.2. To assess the willingness of surgeons to recruit/randomize patients undergoing different surgical procedures.

1.3. To estimate the proportion of eligible patients who consent to randomization.

1.4. To estimate the proportion of patients who adhere to the interventions proposed.

1.5. To estimate follow-up completion rates.

1.6. To inform the calculation of sample size requirements for a full-scale RCT.

PART 2. Embedded qualitative study

2.1. To inform, via qualitative research methods, optimal study design of a full-scale RCT by assessing patient and clinician perspectives on trial conduct, participation, interventions and measurement strategy.

METHODS

PART 1. Main study (Pilot RCT)

This study will be a parallel, two-group, assessor-blind, pilot randomized trial with participants individually allocated on a 1:1 ratio to treatment with either OA or OFA. To maximize applicability of the study to current perioperative care settings, the trial was designed to be pragmatic; i.e. it will be undertaken in routine clinical practice under “real world” conditions. Eligibility criteria will facilitate enrollment of diverse patients

undergoing outpatient surgery (day surgery) and interventions will be delivered with flexibility in medication selection. An embedded qualitative study will be conducted to help optimize trial design based on clinicians' and patients' perspective[22]. The study protocol will be reviewed by the McGill University Health Centre (MUHC) Research Ethics Board and patient recruitment will start after ethics approval. All participants will sign a written consent form and a paper copy of the form will be attached to the patient medical chart. Trial registration and protocol information will be made available at the ClinicalTrials.gov website. The planned flow of participants through the study is summarized in Figure 1. A trial management team (TMT), composed by trial leaders (Drs. Fiore, Baldini and Feldman) and trial managers (Ms Pepa Kaneva, Ms Uyen Do and Mr Charbel El Kefraoui) will meet weekly to discuss the progress of the trial and address any issues that may arise.

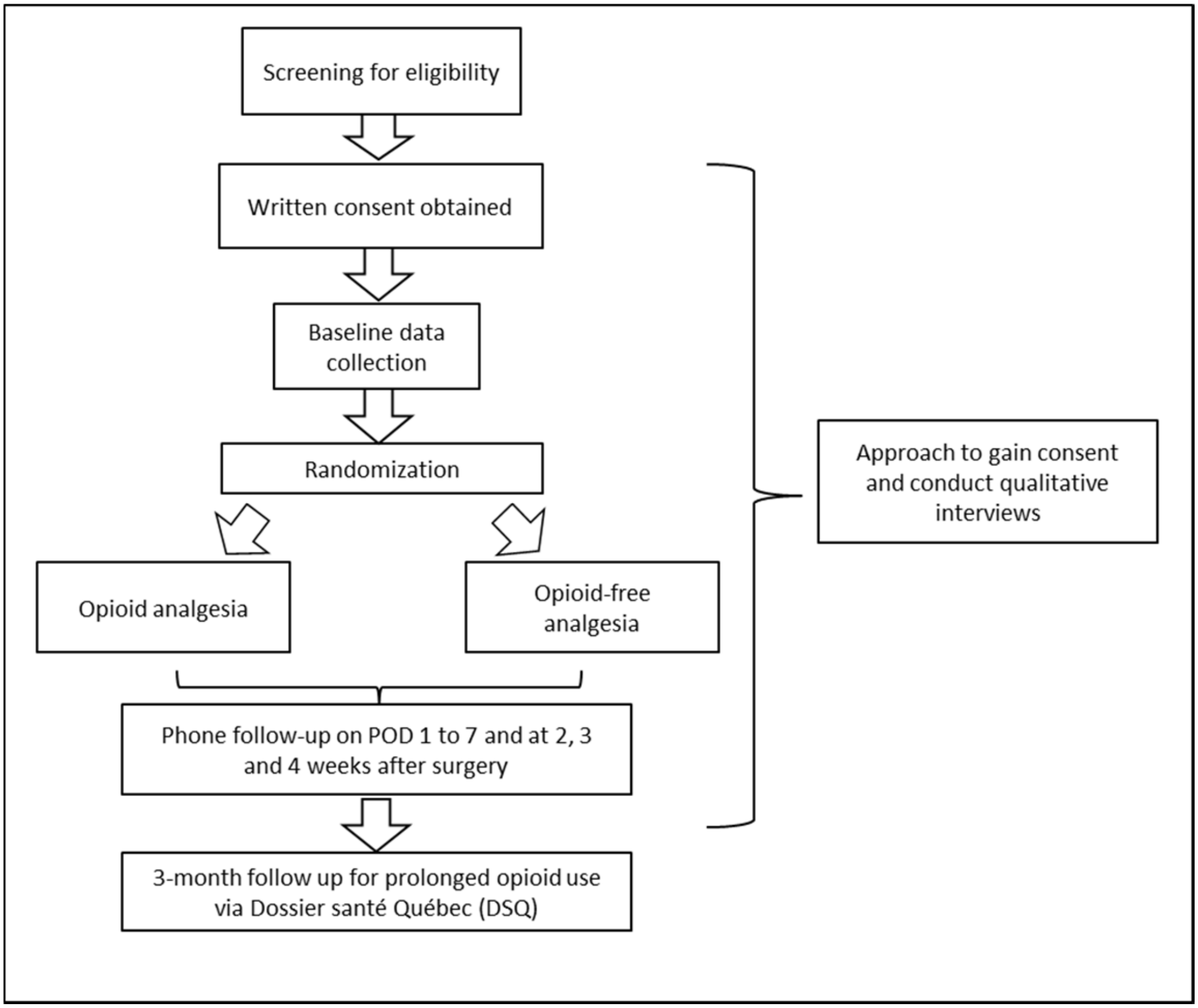

Figure 1. Flow of participants through the study. POD = postoperative day

Patients

Adult patients (over 18 years old) undergoing elective outpatient surgery (with planned discharge same day on the day of the operation) in two sites of the McGill University Health Centre (MUHC) in Montreal, Canada (Montreal General Hospital and Royal Victoria Hospital) will be considered for inclusion. Eligibility will span a wide range of general surgery procedures that are routinely conducted with same day discharge, including procedures in abdominal (i.e., cholecystectomies, hernia repairs) and breast surgery (i.e., lumpectomies, partial and complete mastectomies, axillary node dissections).

As a pragmatic trial, we will keep exclusion criteria to the minimum necessary to ensure both patient safety and internal validity. Patients with intraoperative or early postoperative complications (i.e., diagnosed in the Post-Anesthesia Care Unit (PACU)) that require postoperative hospital stay will be excluded. Other reasons for exclusion are: contraindications to any of the drugs used in the trial according to Health Canada Monographs (i.e. active substance use disorder, pregnancy, severe heart failure, allergy, active symptomatic peptic ulcer or gastrointestinal bleeding, bleeding disorders, severe renal or liver impairment)[23-25], conditions that could interfere with outcome assessment [e.g., cognitive impairment, inability to speak English or French, difficulty to be reached after surgery (e.g., limited access to a telephone or a computer)].

Overview of recruitment and consent procedures

(1) Eligible patients scheduled for elective outpatient general surgery will be informed about the study by their primary surgeon during the preoperative surgical consultation, (2) those who are interested in the study will be advised by the treating clinician that a member of the study group will contact them to discuss the study in detail during their subsequent standard visit to the preoperative assessment clinic or by telephone (if the clinic is bypassed), (3) patients who are eligible and interested in participating will be asked to sign the consent form and complete the study's preoperative questionnaires in the preoperative clinic or at home. In the latter case, consent will be obtained via pre-

paid mail and preoperative questionnaires will be completed online or by phone. It will be up to patients to choose the preferred method of completing the questionnaires.

Trial posters will be displayed in waiting areas of the MGH and RVH preoperative clinics to raise awareness of the study for both patients and clinicians. Study promotional materials are attached to this application (Figure 5-6).

Randomization and blinding

Treatment allocations will be concealed until patients are deemed ready to be discharged home from the PACU – i.e., when a discharge order is signed by the primary surgeon, or a delegated clinician member of his/her team. Randomization will be conducted via a secure web-based randomization service (www.sealedenvelope.com). Research staff will have password-protected access to the randomization website by means of a computer or smart phone. No personal information about participants will be entered in this platform. To yield balanced yet unpredictable groups, randomization will use computer-generated, permuted, balanced blocks of randomly varying size (2, 4 or 6). To achieve group balance for important covariates, randomization will be stratified by procedure type (abdominal, breast). Participants and clinicians will be informed verbally of the treatment allocation at the point of randomization. The primary surgeon, or a delegated clinician member of his/her team, will be responsible for signing a pre-written analgesia discharge prescription in accordance with the treatment that patients have been allocated to.

Participants and treating clinicians (i.e., surgeons, anesthetists, and nurses) will not be blinded to treatment allocation due to the complexity of the medication prescribing strategies. To reduce potential risk of detection bias (systematic differences between groups in how outcomes are determined), outcome assessors will be blinded to treatment allocation. Patient-reported outcomes and treatment adherence data will be collected via self-administered electronic questionnaires distributed using REDCap (http://project-redcap.org/) and completed by patients via smartphone, tablet or personal computer. Electronic outcome data will be transmitted directly to the REDCap database and verified by a blinded assessor. Adherence data will be verified by unblinded study staff. Patients who are not computer savvy, have limited access, or prefer non-

electronic assessment will complete the questionnaires via telephone interviews with a blinded assessor; in this case, data will be recorded in paper forms and subsequently transferred to the REDCap database. Prior to every telephone interview, patients will be reminded not to disclose their allocation status or information about pain medications. To prevent unblinding, telephone follow-ups to monitor treatment adherence will be done by a team member not involved in outcome assessment.

Outcome data that are not patient-reported (e.g., postoperative complications, unplanned healthcare utilization, chronic opioid use) will be obtained from medical records by a blinded assessor. Any inadvertent unblinding will be reported. Effectiveness of blinding will be estimated by asking assessors to guess patients' group allocation at one month after surgery (after the last patient questionnaire is responded). Statistical analysis will also be blinded with information regarding allocation protected by codes that will be revealed only after all analyses are completed.

Interventions

Opioid analgesia (OA) group

Patients randomized to the OA group will receive the current standard of care in the participating centers, which includes the prescription of around-the-clock non-opioid analgesics (acetaminophen and/or NSAIDs/COX-2) and a supply of opioids to be used as a rescue in case of breakthrough pain (i.e., pain that erupts while a patient is already medicated with painkillers). Prior to hospital discharge, patients will undergo a medication education session with the PACU nurse and be advised to fill their prescription at a pharmacy of their preference. Medication education sessions with a nurse prior to discharge are part of standard care at MUHC. In light of the pragmatic nature of this trial, the specific round-the-clock analgesia and rescue opioid regimens will be determined by the patient's primary surgeon considering the surgical procedure, comorbidities and patient's preference. Postoperative pain management strategies currently used at the MUHC are set with input from pain specialists (Alan Edwards Pain Management Unit) and follow Health Canada standards for safety and efficacy[26]. Examples are included in eFigure1.

To confirm if patients randomized to this group are treated according to current standards of care, we will conduct a retrospective chart review of post-discharge analgesics prescribed to patients who underwent the eligible surgeries between September 01 to October 31, 2019. We estimate that, within this 2-month period, the electronic medical charts of approximately 100 patients will be reviewed. Only data regarding the surgical procedure conducted and analgesia regimen prescribed (pain medication received, dosage, frequency of administration, treatment duration) will be collected by the research team.

Opioid-free (OF) analgesia group

Patients randomized to the OFA group will receive a prescription of around-the-clock non-opioid analgesics (Acetaminophen alone or combined with NSAIDs/COX-2). In case of breakthrough pain, rescue analgesia may be provided by (1) increasing doses of non-opioid analgesics, (2) adding non-opioid drugs that were not included in the initial regimen or (3) switching drugs according to single-dose efficacy evidence[27,28] targeting individual variances in analgesia response[29]. As per standard care, prior to hospital discharge, patients will undergo a medication education session with the PACU nurse and be advised to fill their prescription at a pharmacy of their preference. Considering the pragmatic nature of this trial, the specific non-opioid analgesia regimens will be determined by the patient’s primary surgeon considering the surgical procedure, comorbidities and patient’s preference. The pain specialists involved in this trial [Dr. Gabriele Baldini (Anesthesia), Dr. Avinash Sinha (Anesthesia), Dr. Suzanne Morin (Internal Medicine), and Ms Krista Brecht (Alan Edwards Pain Management Unit)] have set potential analgesia strategies for the OFA group, according to Health Canada standards for safety and efficacy [26] (eFigure 2).

Management of persistent pain

As opioid-free analgesia is new to our setting, specific strategies will be implemented to ensure that patients are receiving adequate pain management during the pilot trial. A ‘hotline’ (dedicated mobile phone that will be kept with study staff in shifts) will be available 24/7 in case patients experience persistent pain despite the use of rescue analgesia. When this line is called, study staff will inform patients about the

management options available according to their treatment allocation. An information sheet containing the ‘hotline’ contact details will be provided to patients prior to PACU discharge (see **Discharge Information Sheet – Opioid-free Group**).

Patients in the opioid-free group will have a back-up prescription of opioids (regimen decided by the primary surgeon) faxed to the 24h pharmacy closest to their residence. This prescription will be faxed upon patient discharge from the hospital, with a brief letter informing the study and ethics approval (see **Information Sheet for Pharmacy**). When a patient calls the study staff reporting persistent pain, they will be informed about the availability of the prescription and the pharmacy address. To prevent patients to fill their opioid prescription ‘just in case’, they will not be informed about the availability of the prescription unless they report persistent pain. When the prescription is filled, education about the use of opioids will be given by the pharmacist as per routine pharmacy services. If pain persists despite the use of opioids, patients will be advised to proceed according to the management of persistent pain in the opioid group, as described below.

As per the institutions’ current practice, patients in the opioid group who experience persistent pain will be advised to call their primary surgeon’s office/clinic during working hours (weekdays, 8AM to 4PM) or visit a hospital emergency room (ER) for further evaluation (after-hours and weekends). If an ER visit is required, patients will be asked to give preference to visiting the ER of the hospital where his/her surgery had been performed. An information sheet containing specific instruction will be provided to patients prior to PACU discharge (see **Discharge Information Sheet – Opioid Group**). Changes of initial prescription will be entirely up to the patients’ surgical team and/or ER physician.

Adherence and study discontinuation

Treatment adherence (i.e., patients in each group taking their pain medications as prescribed) will be monitored via self-administered electronic questionnaires distributed using REDCap (http://project-redcap.org/) and completed by patients via smartphone, tablet or personal computer from postoperative day (POD) 1 to POD 7 and at 2, 3 and 4 weeks after surgery. Electronic adherence data will be transmitted directly to the

REDCap database and verified by unblinded study staff. Patients will also be offered the option to respond to adherence questionnaires via telephone; in this case, data will be recorded in paper forms by unblinded staff and subsequently transferred to the REDCap database. Patients will be instructed to take medications for postoperative pain only in accordance with the initial discharge prescription or based on prescriptions given by healthcare providers after hospital discharge. If patients desire discontinuation of any of the study medications, they will be advised to discuss other medication options with the surgical team and/or their outpatient care provider. Surgeons may change pain medications or put an end to a patient participation in the trial at any time if he/she considers this to be in the best interest of the patient.

Other aspects of perioperative care

Surgical techniques, anesthesia procedures, or preoperative/intraoperative analgesia protocols will be left to the discretion of the attending surgeon and anesthesiologist to best reflect routine clinical practice. However, technical details about the surgery, anesthesia and perioperative analgesia interventions (including preoperative use of analgesics in preparation for surgery, e.g., gabapentin, and intraoperative use of local anesthetics infiltration or blocks) will be obtained from electronic medical records and recorded for study purpose. Any nonpharmacological therapies for pain recommended by the surgical team or outpatient healthcare providers (e.g., heat or ice compress, acupuncture, massage therapy) will be permitted and recorded during follow-up assessments. Considering the pragmatic nature of this trial, medication education provided by nurses and all other aspects of perioperative care will be according to the institutions' routine practice, which include detailed care pathways for selected surgical procedures (http://www.muhcpatienteducation.ca/surgery-guides.html).

Measurement Strategy

As a pilot RCT, this study will primarily focus on feasibility outcomes. Clinical outcomes will be assessed secondarily to inform the measurement strategy and sample size requirements for a future full-scale RCT.

Assessment of feasibility outcomes (primary)

A full-scale RCT be deemed feasible if, during the pilot study period (4 months):

- At least 70% of patient undergoing the outpatient general surgery procedures of interest are eligible to be randomized.
- At least 90% of the surgeons who agreed to have their patients randomized will comply with the agreement, i.e., not change their minds (see section 'pilot study sample size and feasibility' below).
- At least 50% of eligible patients agree to participate in the study and are randomized.
- At least 80% of the randomized patients comply with their allocated treatment (i.e. will take their pain medications as prescribed).
- At least 80% of the patients randomized complete outcome assessment at 30-days after surgery.
- Among patients who complete outcome assessments, the proportion of missing data is less than 10% (i.e., non-response to questionnaires or specific questionnaire items).

To determine recruitment rates, study staff will keep a screening log of patients approached, patients who fulfill eligibility criteria and those who do not fulfill eligibility criteria. Reasons for ineligibility will be recorded. This log will also record information about eligible patients who were successfully recruited, and those who were not recruited despite being eligible. In the event of surgeons opting for not recruiting patients despite eligibility, rates and reasons will be recorded. Adherence to treatment will be assessed by comparing patients' analgesia prescription at discharge to self-reported analgesic intake at each time-point of assessment. Follow-up completion rates and missing outcome data will be computed based on REDCap entries (date- and time-stamped). Patients will be considered to have withdrawn from the trial if they miss three consecutive assessments and then permanently stop responding the questionnaires. Reasons for patients not consenting participation, not completing follow-ups or withdrawing from the trial will be recorded whenever possible.

Assessment of clinical outcomes (secondary)

Our clinical outcome measurement strategy was informed by the World Health Organization (WHO)'s International Classification of Functioning and Disability (ICF) and will cover constructs in the domains of impairment, activity limitation and participation restriction[30]. A range of outcome measures were identified as being potentially useful for a full-scale trial on OA versus OFA. One of the main goals for this pilot study is to determine their appropriateness and usability. Due to the subjective nature of pain and response to analgesia, we placed special focus on PROMs, i.e., reports of health status coming directly from the patient. Preference was given to measures that (1) have validity evidence supporting their use in surgical populations[31,32], (2) have been recommended by surgery, anesthesia and pain societies[32-34], (3) use scoring systems based on modern psychometric methods (Item-Response Theory, Rasch analysis)[35], (4) have been used in previous literature on postoperative/opioid analgesia, (5) have short recall periods (preferably 24 hours, no more than 7 days) and (6) have low response burden (i.e. are brief). Author-generated questions will be used to assess constructs that have not been addressed by existing measures or that have been addressed in a context that is not applicable to the current study. The outcome measures addressed in this study include: the Brief Pain Inventory Short-Form[36-38], time to stopping pain medication[37], Patient-Reported Outcomes Measurement Information System 29 Profile (PROMIS-29); domains: physical function, anxiety, depression, fatigue, sleep disturbance, social roles and activities, pain intensity and pain interference)[33,38,39], Perioperative Opioid-Related Symptom Distress Scale[40], Prescription Opioid Misuse Index[41], recovery from surgery (author-generated question), return to work or normal activities (author-generated question), impression of treatment effectiveness (author-generated question), satisfaction with the pain treatment received (author-generated question), 30-day postoperative complications[42,43], 30-day unplanned healthcare utilization, 30-day adverse drug events[44-46], and prolonged opioid use (3-month follow-up). See eTable 3 for a complete description of these measures.

Patient-reported outcome data will be obtained via (1) electronic questionnaires or (2) telephone interviews, according to the patient's preference. Electronic questionnaires will be completed remotely (via smartphone, tablet or personal computer) using our REDCap platform. A link to the daily questionnaires will be distributed to patients via

text message or email (according to the patient's preference) in the morning, with up to 3 reminders sent in case of no response. Participants will be asked to, preferably, complete the questionnaires in the morning to prevent bias associated to chronobiological variations in pain[47]. Patients who opt for non-electronic assessment will complete the questionnaires via telephone interviews, preferably conducted before 12PM. Information regarding postoperative complications and unplanned healthcare utilization will be obtained via patient self-report (week 4) and verified using electronic medical records. Information regarding opioid prescription dispensing will be obtained using Dossier Santé Québec (DSQ), accessed by a physician-collaborator (Dr. Mohsen Alhashemi, Minimally Invasive Surgery Fellow) upon patient authorization via study consent form. Details of our follow-up schedule are summarized in Figure 4.

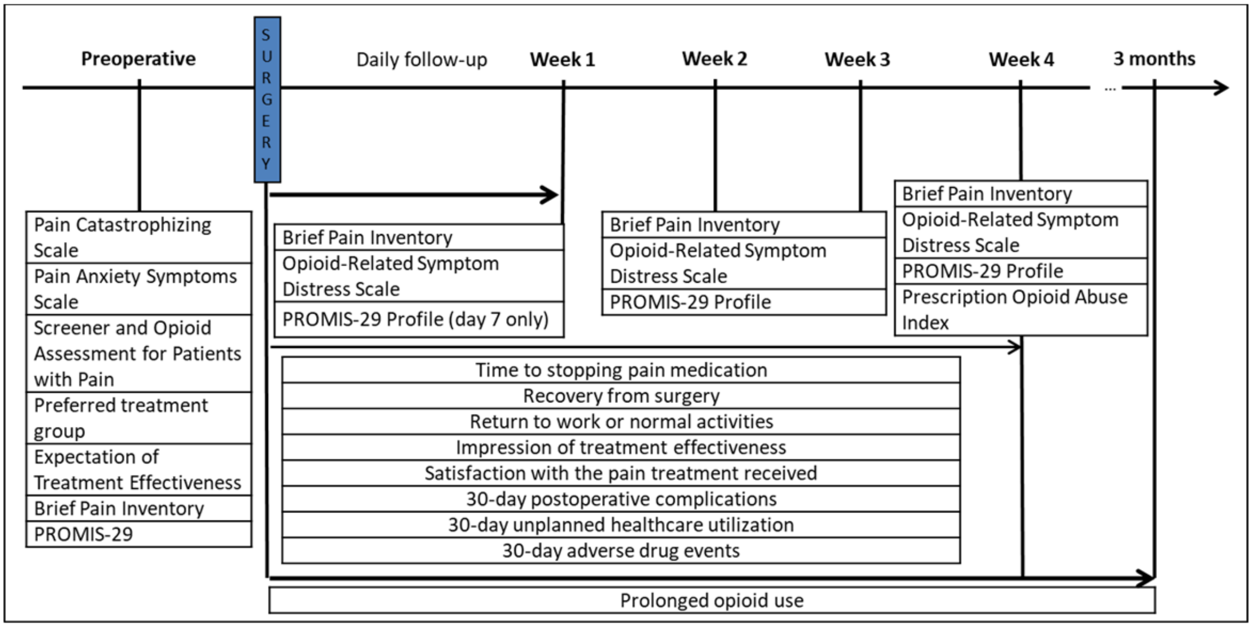

Figure 2. Patient follow-up schedule, POD = Postoperative day

Preoperative screening measures

These measures focus on potential prognostic factors for difficult pain control, need for opioid analgesia and opioid seeking behavior after surgery. In a future full-scale RCT, they may help refining inclusion and exclusion criteria, as well as setting stratification strategies to balance important covariates between treatment groups. Screening measures addressed in this pilot study include: demographic and operative information (data also used to characterize the patient population), the Pain Catastrophizing

Scale[48,49], the Pain Anxiety Symptoms Scale (short version)[19,50], the Screener and Opioid Assessment for Patients with Pain (SOAPP)[51], preferred treatment group (author generated question) and expectations for treatment effectiveness (author generated question). See eTable 2 for a complete description of these screening measures.

Data management plan and analysis

Data collection and storage will be according to the MUHC's Regulatory Framework in Health Research, which is in line with provincial and federal legislations. All data will be entered and stored in a password-protected system of electronic data capture (REDCap, http://project-redcap.org/) and quality will be ensured via in-built validation checks (i.e., missing data, out-of-range values and invalid responses). Data analysis will be conducted using Stata version 14 software (StataCorp). Analysis and trial reporting will be according to the Consolidated Standards of Reporting Trials (CONSORT) Guidelines extension for Pilot and Feasibility Trials[52].

Data generated from the pilot study will help inform a full-scale RCT by testing the study procedures; therefore, no inferential statistical analyses will be performed to compare groups. Continuous variables will be summarised using means, standard deviations (SDs), medians, lower and upper quartiles, minimum, maximum and number of observations. Categorical variables will be summarised using frequencies and percentages. To address feasibility, descriptive statistics of patients approached, screened, eligible, consented and randomised, treatment adherence and follow-up completion rates will be computed. Completeness of follow-up will be compared between trial arms. Reasons for non-consent, exclusion and trial withdraw will be recorded and reported. Baseline data will be summarized descriptively to assess comparability between treatment arms and to highlight any differences between patients who were randomized, who withheld consent and who did not meet eligibility criteria. Analyses of postoperative outcomes will be exploratory, descriptive and follow the intention-to-treat principle, with all patients analyzed in their assigned treatment group.

The primary outcome measure to be addressed in the full-scale RCT will be informed by data from this pilot trial. Decision will be based on acceptability and relevance to patients and clinicians (qualitative study described below), completion rates, evidence of

measurement properties according to previous literature, effect sizes and sample size requirements. There are no planned interim data analyses; however, if the TMT identifies that recruitment, randomization and data collection are below target, strategies will be implemented to improve progress. Any changes to methods after trial commencement will be documented and reported. Any future revisions to protocol and consent forms will be implemented only after IRB approval.

Pilot study sample size and feasibility

This pilot trial is not confirmatory; therefore, a formal sample size calculation was not conducted. In accordance to previous recommendation that at least 70 measured participants are required for estimating SDs of continuous measures[53], we aim to recruit and obtain outcome data from 80 patients (40 per group), allowing for a ~15% attrition rate. This sample size is also in line with recommendations regarding the minimal number of participants required to identify feasibility issues[54].

This pilot study will be conducted in two high volume centres where approximately 1000 eligible outpatient abdominal and breast surgeries are performed every year. In May 2019, we circulated our study protocol (draft) and conducted an electronic survey of surgeons across the two institution; 10 surgeons (7 General, 3 Breast) agreed to have their patients recruited for this pilot trial. Based on previous trial experience, approximately 60% of the patients approached during the trial period will be eligible and agree to participate. Therefore, we estimate that 80 participants could be feasibly enrolled in 4 months. With additional 3 months required to finalize patient follow-up and the time required for data analyses and report/manuscript preparation, we anticipate that the time required to complete this study is approximately one year. Specific details about our timeline are presented in.

PART II. Embedded qualitative study

A qualitative study involving patients and clinicians will be integrated within this pilot trial to provide further fundamental insights into the design of a future full-scale RCT.

Study objective:

The objective of this study is to inform, via qualitative research methods, optimal study design of a full-scale RCT by assessing patient and clinician perspectives on trial conduct, participation, interventions and measurement strategy.

Research questions:

1. What are participants and non-participants' perspectives on the pilot trial conduct, participation (or non-participation), interventions, and measurement strategy?
2. What are clinicians' perspectives on the acceptability of the pilot trial, experience operationalizing the study in practice, treatment effectiveness, challenges that may impact on the feasibility of a full-scale RCT, and areas for improvement in the future trial design?

Interviews will be conducted until thematic saturation is reached (i.e., the point in data collection after which no new themes emerge), accounting for a minimal targeted sample of five patients and five clinicians. Our methodological approach will follow Braun and Clarke's guideline for the use of thematic analysis in qualitative studies [55]. As demonstrated by O'Cathain et al. (2013), qualitative analysis is a valuable tool to optimize interventions in comparative-effectiveness research. Reporting of this qualitative study will be in line with the Consolidated Criteria for Reporting Qualitative Studies (COREQ) guidelines [56].

Interviews with patients

A sub-sample of patients who participated in the recruitment process for the pilot trial will be invited to participate in one-on-one qualitative interviews. Patients who do not consent to randomization in the trial will also be invited to participate in the interviews as they may provide relevant insights regarding the consent process and study acceptability. In order to capture the heterogeneity of outpatient general surgery procedures and improve sample representativeness, we will use a quota sampling method [57] targeting patients representing a broad spectrum of demographic, clinical and surgical characteristics (Table 1). Patients will be offered the opportunity to be interviewed face-to-face or by telephone. Patients will be informed about the qualitative interviews during preoperative recruitment and those who are interested will be

contacted after their involvement with the trial. A consent form specific to the qualitative study will be signed prior to the interviews. To ensure accurate recall, patients will be interviewed no later than 6 weeks after their surgery. Interviews will focus on (1) acceptability of the study, (2) personal experience with the process of recruitment and randomization, (3) reasons for not accepting randomization (where appropriate), (4) perceived value and experiences with the intervention, (5) perceived value and experienced with the outcome assessments, (6) reasons for not completing outcome assessment (where appropriate), and (7) areas for improvement in trial design.

Table 1. Qualitative study interviews: Target sampling quotas for patients

Characteristic	Targeted quota
**Age**	
≤ 30 years	≥20%
≥ 65 years	≥20%
**Gender**	
Male	≥40%
Female	≥40%
**Surgery**	
Abdominal	≥20%
Breast	≥20%
**Education**	
Low (less than high school)	≥20%
High (university degree or above)	≥20%
**Employment status**	
Working/studying	≥30%
Retired	≥30%
**Postoperative complications after hospital**	
Yes	≥10%
No	≥60%
**Consented randomization**	
Yes	≥70%
No	≥20%

Patient recruitment process

Subsequent contact for participation in the qualitative study will be made upon patient authorization. Patients will be approached as follows, depending on whether they agreed or not to participate in the pilot RCT:

(1) Patients who agreed to participate in the pilot RCT and signed the informed consent form: In the consent form for the Pilot RCT (see " **Informed consent form - Pilot RCT** "), we will ask whether we have permission to contact the patient to inquire about participation in the qualitative part of this project (check "YES" or "NO"). Those who checked "YES" will be contacted after their participation in the Pilot RCT. A separate informed consent form (See "**Informed consent form - Interview with patients**") will be signed prior to the qualitative interview.

(2) Patients who refused to participate in the Pilot RCT: Those who refused to participate in the Pilot RCT will be informed about the qualitative study and be offered to sign a "**Permission to contact form**" if they agree to be contacted regarding participation in the qualitative study. Patient who agree to participate will sign separate informed consent form prior to the qualitative interview (See "**Informed consent form - Interview with patients**").

Interviews with clinicians

A sample of clinicians (surgeons, nurses, anesthesiologists) involved in the perioperative care (i.e., prescription, education about postoperative analgesia) of patients undergoing the surgeries of interest in this trial will be invited to participate in one-on-one qualitative interviews. Interviews will be conducted face-to-face or by telephone after informed consent is obtained. In order to improve sample representativeness, we will use a quota sampling method [57] targeting clinicians representing a broad spectrum of demographic and professional characteristics (Table 2). Interviews will be conducted within the period of patient recruitment to ensure accurate recall. Interviews with clinicians will focus on (1) acceptability of the study, (2) experience operationalizing the study in practice (i.e., recruiting patients and providing interventions), (3) reasons for not recruiting patients (where appropriate), (4) perspectives on treatment effectiveness, (5) local issues that may impact on the feasibility of a full-scale RCT and (6) areas for improvement in trial design.

Table 2. Qualitative study interviews: Target sampling quotas for clinicians

Characteristic	Targeted quota
**Years of clinical experience (after residency)**	
≤ 5 years	≥20%
≥ 15 years	≥20%
**Practice location**	
Montreal General Hospital	≥40%
Royal Victoria Hospital	≥40%
**Training background**	
Surgery	≥60%
Anesthesia	≥20%
Nursing	≥20%
**Received formal research training (Masters, PhD)**	
Yes	≥40%
No	≥20%
**(For surgeons) Specialty**	
General (abdominal)	≥20%
Breast Surgery	≥20%
**(For surgeons) Had patients involved in the trial**	
Yes	≥40%
No (or low randomization rate, <3 patients)	≥20%

Clinician recruitment process

All clinicians (surgeons, nurses, anesthesiologists) who care for patients undergoing the surgeries eligible for this study will be informed about the qualitative study by their respective Division Chiefs (see team of collaborators in "Expertise and Resources Available"). Clinicians who meet eligibility criteria will be contacted via email by a member of the study team. Their contact information will be obtained via the McGill and/or MUHC website. Those who agree to participate will sign a consent form (See "**Informed consent form – Interview with Clinicians**") prior to the qualitative interview.

Interview procedures, data management and analysis

Interviews will follow semi-structured guides designed with open-ended questions to elicit patients' and clinicians' personal perspectives about the trial. Initial guides will be

drafted by the trial steering committee and pilot tested for terminology, flow and redundancy. All interviews will be digitally recorded using high quality audio equipment and transcribed verbatim by a third-party ISO certified transcription company. Analysis of interview data will be conducted via inductive thematic analysis informed by Braun and Clarke (2006)[55]. Thematic analysis is a method used to identify, analyze, and report themes and subthemes within the interviews to provide a rich description of the qualitative data. The inductive approach to thematic analysis is data-driven, where the themes will be derived from within the data themselves and no pre-existing coding framework will be applied during analysis. Based on data obtained from the first interviews, two independent researchers (coders) will code each interview transcription and search for recurring themes. The coding process will be conducted using the software MAXQDA 12 (VERBI GmbH, Berlin, Germany). For every two transcripts coded, coders will meet to (1) compare the codes assigned, (2) revise the codes iteratively as new information emerges, (3) cluster the codes (via thematic mapping) into initial themes and sub-themes to inform the subsequent development and refinement of themes, and 4) generate a clear definition and name for each of the theme. Assessment of saturation will be conducted iteratively (after every 2 interviews) using a saturation grid[58].

The findings from this qualitative study will be regularly fed back to the trial steering committee so that aspects of the pilot study conduct can be reviewed iteratively where appropriate. Themes for which saturation is reached will be classified as meaningful issues to inform the optimal design of the full-scale RCT.

Summary of sample size estimates

PART I. Main study (Pilot RCT)

80 participants (40 per group).

PART II. Embedded qualitative study

20 participants (estimate) - A minimal of 10 participants (5 patients, 5 clinicians) will be recruited but the total sample may vary according to data saturation.

Total sample size

100 participants (estimate).

EXPERTISE AND RESOURCES AVAILABLE

This project builds on the expertise of scientists and clinicians with extensive experience and knowledge in the fields of surgery and postoperative analgesia. Dr. Julio Fiore Jr (Outcomes Researcher) is the principal investigator and primarily responsible for writing the study protocol. He will be in charge of the overall coordination and supervision of all aspects of this pilot RCT, including recruitment, randomization and data management. He has substantial experience with the design and conduct of pilot and full-scale RCTs. Dr. Gabriele Baldini (Anesthetist) and Dr. Liane Feldman (Surgeon) are co-investigators and knowledge users (i.e. prescribers of postoperative pain medications). They will be responsible for supervising all clinical aspects of the study (i.e. analgesia interventions) and for liaising with clinicians across both study sites. Our team of collaborators bring in a wide range of clinical and research expertise to this project: RCTs (Dr. Kaberi Dasgupta, Physician/Epidemiologist), acute pain assessment and management (Dr. Suzanne Morin, Physician/Epidemiologist), postoperative analgesia (Dr. Avinash Sinha, Anesthetist; Ms Krista Brecht, Pain Nurse), surgery (Dr. Sarkis Meterissian, Breast Clinic Director; Dr. Mohsen Alhashemi, Minimally Invasive Surgery Fellow), opioid misuse (Dr. Marc Martel, Psychologist) and qualitative research (Dr. Fatemeh Rajabiyazdi, Postdoctoral Fellow/Qualitative Researcher). Statistical support from the RI-MUHC Biostatistics Support Unit has been sought and incorporated in this pilot trial in preparation for a full-scale RCT.

The project will be coordinated by the Steinberg-Bernstein Centre for Minimally Invasive Surgery, based at the Montreal General Hospital. The centre offers dedicated office space (100m^2) with computer facilities for data collection and warehousing and employs a full-time research coordinator (Ms. Pepa Keneva, MSc). Two master's students (Ms Uyen Do and Mr Charbel El Kefraoui) will coordinate the day-to-day management of the project at the two sites under the supervision of Drs. Fiore, Baldini and Feldman. Our

experienced multidisciplinary team has all the necessary elements (i.e. infrastructure, methodological and context expertise) to successfully conclude this project.

ANTICIPATED CHALLENGES AND MITIGATION STRATEGIES

Prescription of opioids to treat breakthrough pain after surgery is imbedded in Canada' healthcare culture. For this reason, we cannot exclude that (1) certain clinicians may be wary of discharging patients without an opioid prescription and (2) ethical issues may be raised anticipating a negative impact on pain outcomes. However, considering the current opioid crisis, changes have been observed in the paradigm of 'mandatory opioid prescription' as some surgeons across the MUHC began managing pain after outpatient general surgery using only non-opioid drugs. According to their personal experience, this practice did not increase unplanned healthcare visits due to uncontrolled pain and, importantly, satisfaction with pain control reported during scheduled postoperative visits seems unchanged in comparison to when opioids were regularly prescribed. Besides this anecdotal data, preliminary results from our scoping review suggest that previous comparative studies do not support the value of prescribing opioids after outpatient surgery[17-19] – these results, however, must be confirmed in a formal systematic review/meta-analysis. In other patient populations such as chronic musculoskeletal pain and acute extremity pain, the role of opioid analgesia has also recently been questioned in large RCTs showing non-superiority[38,59] and increased adverse events[38]. In light of this evidence and considering the ongoing paradigm change at a local level, this pilot trial gained support from key stakeholders in our surgical departments and divisions who are committed to encouraging recruitment across both study sites.

As certain surgeons may heavily rely on opioids to treat postoperative pain, we anticipate that some may refuse to recruit selected patients or refuse to recruit patients altogether. Similarly, some patients may be doubtful about the efficacy of pain treatment without opioids and refuse randomization. This issue will be addressed by comparing demographic and surgical data of randomized patients versus non-randomized patients. Differences may suggest that our results are not generalizable to certain surgical populations, indicating venues to improve our patient selection criteria and/or recruitment process. Our integrated qualitative study including interviews with patients

who refused randomization and surgeons with low recruitment rates will provide fundamental insights into the strategies to mitigate these potential issues. The qualitative study will also provide relevant information to optimize our measurement strategy, which currently includes daily follow-up in the first 7 days after surgery. The use of daily outpatient follow-up assessment has been successful in a recent RCT on postoperative analgesia[37] but, if proven unfeasible in our setting, strategies will be implemented to reduce patient burden (e.g., reducing follow-up frequency).

Finally, surgeons from different specialities may give preference to different non-opioid drugs, e.g. NSAIDs/COX-2 may be avoided by some surgeons due to potential risk of bleeding[60], while others may be concerned about risk of liver failure when using acetaminophen[61]. In line with the pragmatic nature of this trial, surgeons will have the freedom to, within the analgesia principles of each intervention group, choose the regimen that they find most appropriate according to surgical procedure, comorbidities and individual preference. To ensure safety, analgesia prescriptions will follow Health Canada monographs for maximum dosages and length of treatment[26]. Potential treatment adverse events will be identified and reported according to internationally accepted standards supported by Health Canada[44-46,62].

DATA COLLECTION AND CONFIDENTIALITY

Retrospective chart review: All the information collected during our preliminary chart review will remain confidential to the extent required and provided by law. A study ID number will be assigned to each patient's chart. No code linking patient identifiers to patient data will be kept and it will not be possible to identify patients.

Pilot Trial: All data collected in our pilot trial will be entered and stored in a password-protected system of electronic data capture (REDCap; Research Electronic Data Capture, hosted at Research Institute of MUHC), and subsequently transferred to the statistical program for analysis. A study ID number will be assigned to each participant. Information collected in paper-based forms will be kept in locked cabinets within a locked office (R2-111). Participants will be identified by a code to protect their identity. A document linking the codes to the participants' identity will be kept separately in a password protected file, which can only be accessed by the study staff.

All data will be kept under safe storage for 7 years and then deleted, shredded or incinerated. Only investigators will have access to the data. Furthermore, the results and the project may be published, but patients' identity will not be revealed.

KNOWLEDGE TRANSLATION (KT) PLAN

Results from this pilot trial will inform the planning and commissioning of a future full-scale RCT on opioid-free analgesia after outpatient general surgery. If proven feasible, this full-scale RCT will inform guidelines targeting sustainable changes in surgical care to mitigate the negative downstream effects of postoperative opioid overprescription. Our findings will be disseminated according to CIHR's Guide to Knowledge Translation (KT) Planning[63] and target a broad audience of surgeons, anesthetists, nurses, pharmacists, surgical outcomes scientists and research funders. Our KT strategies include, but are not limited to, conference presentations (local, national and international), publication of a peer-reviewed paper, and diffusion of findings in websites, newsletters and social media platforms. As opioids are part of standard postoperative care in North America, we believe that our study will contribute feasibility data to support and encourage further opioid-free analgesia research beyond our immediate research setting in Canada and internationally (i.e., the United States).

SIGNIFICANCE

The overprescription of opioids to surgical patients is recognized as one of the driving forces behind the current opioid crisis. Patients undergoing outpatient general surgery are frequently prescribed opioids to be taken at home postoperatively, but this practice is not supported by evidence. Alternatives to opioids are often overlooked by Canadian surgeons, while they should be incorporated as the foundation of postoperative analgesia whenever possible. If proven effective in a future full-scale RCT, the use of opioid-free analgesia after outpatient surgery may ultimately contribute to preventing opioid-related harms. Hence, the pilot study described in this protocol is an essential first step for building a strong body of evidence to mitigate the negative downstream effects of postoperative opioid overprescription in Canada.

AMENDMENTS TO THE PROTOCOL AFTER INITIAL ETHICS APPROVAL

Change	Reason
**October 2019. Prior to patient recruitment**<br>***Retrospective chart review***	• To confirm that patients randomized to OA group are treated according to current standards of care, a retrospective chart review was conducted to collect data on post-discharge analgesics prescribed to patients who underwent the eligible surgeries in 2019 [period of January 01 to December 31, 2019]. This data (not reported in the manuscript) supported that patients in the OA group were treated according to standard care.
**October 2019. Prior to patient recruitment**<br>***Randomization strategy***	• After discussion with surgeons, the team realized that the randomization of patients in the PACU (with discharge prescriptions written right before hospital discharge) would be impractical as surgeons often write their prescriptions in the OR after skin closure. For this reason, randomizations were conducted in the OR.
**September 2020. After patient recruitment**<br>***Knowledge translation plan***	• After discussion, the team decided that the two components of this pilot study (quantitative and qualitative) would be reported in separate manuscripts.
**June 2021. After patient recruitment**<br>***Outcome measure/data analysis***<br>Data on overall impression of treatment effectiveness at each postoperative timepoint.	• We noticed that this author-generated question was accidently excluded from the final version of the Redcap questionnaire distributed to patients. Therefore, these data were not analyzed or reported in the manuscript. Impressions about treatment effectiveness were detected via other patient-reported questionnaires.
**June 2021. After patient recruitment and data analysis**<br>***Outcome measure/data analysis***<br>Data regarding satisfaction with pain management at postoperative week 4	• After data analyses, the team realized that findings regarding satisfaction with pain management at postoperative week 4 were redundant (did not add relevant information in comparison to the data reported by patients on week 1). For this reason, this information was

	not reported in the manuscript. This data would not be useful as it is subject to recall bias given that most patients do not use pain medications beyond week 1.

REFERENCES

1. International Narcotics Control Board. *Report 2019*. 2019. Accessed March 1, 2021. https://www.incb.org/documents/Publications/AnnualReports/AR2019/Annual_Report_Chapters/English_ebook_AR2019.pdf

2. Canadian Institute of Health Information. Pan-Canadian trends in the prescribing of opioids and benzodiazepines, 2012 to 2017. https://www.cihi.ca/sites/default/files/document/opioid-prescribing-june2018-en-web.pdf.

3. Canadian Institute of Health Information. National report: Apparent opioid-related deaths in Canada (January 2016 to December 2018). Accessed July 31, 2019.

4. Canadian Centre on Substance Use and Addiction. *Canadian Substance Use Costs and Harms*. 2020. Accessed March 1, 2021. https://www.ccsa.ca/sites/default/files/2020-06/CSUCH-Canadian-Substance-Use-Costs-Harms-Report-2020-en.pdf

5. Government of Canada. Federal actions on opioids to date. Accessed March 1, 2021. https://www.canada.ca/en/health-canada/services/substance-use/problematic-prescription-drug-use/opioids/federal-actions/overview.html

6. Alam A, Gomes T, Zheng H, Mamdani MM, Juurlink DN, Bell CM. Long-term analgesic use after low-risk surgery: a retrospective cohort study. *Arch Intern Med*. 2012;172(5):425-30. doi:10.1001/archinternmed.2011.1827

7. Sun EC, Darnall BD, Baker LC, Mackey S. Incidence of and Risk Factors for Chronic Opioid Use Among Opioid-Naive Patients in the Postoperative Period. *JAMA Intern Med*. 2016;176(9):1286-93. doi:10.1001/jamainternmed.2016.3298

8. Castoro C, Berlinato L, Baccaglini U, Drace CA, McKee M. *Day surgery : making it happen*. 2007. Accessed March 1, 2021. http://www.who.int/iris/handle/10665/107831

9. United States for Non-Dependence. *An analysis of the impact of opioid overprescribing in America*. 2017. Accessed March 1, 2021. https://www.planagainstpain.com/wp-content/uploads/2017/09/PlanAgainstPain_USND.pdf

10. Jiang X, Orton M, Feng R, et al. Chronic Opioid Usage in Surgical Patients in a Large Academic Center. *Ann Surg*. 2017;265(4):722-727. doi:10.1097/sla.0000000000001780

11. Bicket MC, Long JJ, Pronovost PJ, Alexander GC, Wu CL. Prescription Opioid Analgesics Commonly Unused After Surgery: A Systematic Review. *JAMA Surg*. 2017;152(11):1066-1071. doi:10.1001/jamasurg.2017.0831

12. Hughes AWM, Lipari RN, Bose J, Copello EAP, Kroutil LA. Prescription Drug Use and Misuse in the United States:

Results from the 2015 National Survey on Drug Use and Health 2016;

13. Lindenhovius AL, Helmerhorst GT, Schnellen AC, Vrahas M, Ring D, Kloen P. Differences in prescription of narcotic pain medication after operative treatment of hip and ankle fractures in the United States and The Netherlands. *J Trauma*. 2009;67(1):160-4. doi:10.1097/TA.0b013e31818c12ee

14. Chapman CR, Stevens DA, Lipman AG. Quality of postoperative pain management in American versus European institutions. *J Pain Palliat Care Pharmacother*. 2013;27(4):350-8. doi:10.3109/15360288.2013.846955

15. Helmerhorst GT, Lindenhovius AL, Vrahas M, Ring D, Kloen P. Satisfaction with pain relief after operative treatment of an ankle fracture. *Injury*. 2012;43(11):1958-61. doi:10.1016/j.injury.2012.08.018

16. Moore RA, Derry S, McQuay HJ, Wiffen PJ. Single dose oral analgesics for acute postoperative pain in adults. *Cochrane Database Syst Rev*. 2011;(9):Cd008659. doi:10.1002/14651858.CD008659.pub2

17. Mitchell A, McCrea P, Inglis K, Porter G. A randomized, controlled trial comparing acetaminophen plus ibuprofen versus acetaminophen plus codeine plus caffeine (Tylenol 3) after outpatient breast surgery. *Ann Surg Oncol*. 2012;19(12):3792-800. doi:10.1245/s10434-012-2447-7

18. Mitchell A, van Zanten SV, Inglis K, Porter G. A randomized controlled trial comparing acetaminophen plus ibuprofen versus acetaminophen plus codeine plus caffeine after outpatient general surgery. *J Am Coll Surg*. 2008;206(3):472-9. doi:10.1016/j.jamcollsurg.2007.09.006

19. Helmerhorst GTT, Zwiers R, Ring D, Kloen P. Pain Relief After Operative Treatment of an Extremity Fracture: A Noninferiority Randomized Controlled Trial. *J Bone Joint Surg Am*. 2017;99(22):1908-1915. doi:10.2106/jbjs.17.00149

20. Kistin C, Silverstein M. Pilot Studies: A Critical but Potentially Misused Component of Interventional Research. *Jama*. 2015;314(15):1561-2. doi:10.1001/jama.2015.10962

21. Government of Canada. The new canadian drugs and substances strategy. Accessed March 1, 2021. https://www.canada.ca/en/health-canada/news/2016/12/new-canadian-drugs-substances-strategy.html

22. O'Cathain A, Thomas KJ, Drabble SJ, Rudolph A, Hewison J. What can qualitative research do for randomised controlled trials? A systematic mapping review. *BMJ Open*. 2013;3(6)doi:10.1136/bmjopen-2013-002889

23. Health Canada. Basic product monograph information for Nonsteroidal Anti-Inflammatory Drugs (NSAIDs). Accessed March 1, 2021. https://www.canada.ca/en/health-canada/services/drugs-health-products/drug-products/applications-submissions/guidance-documents/nonsteroidal-anti-inflammatory-drugs-nsaids/guidance-document-basic-product-monograph-information-nonsteroidal-anti-inflammatory-drugs-nsaids.html

24. Health Canada. Revised guidance document: Acetaminophen labelling standard. Accessed March 1, 2021. https://www.canada.ca/en/health-canada/services/drugs-health-products/drug-products/applications-submissions/guidance-documents/revised-guidance-document-acetaminophen-labelling-standard.html

25. Health Canada. Scientific advisory panel on ppioid use and contraindications (SAP-OUC). Accessed March 1, 2021. https://www.canada.ca/en/health-canada/services/drugs-health-products/drug-products/scientific-expert-advisory-panels/opioid-use-contraindications/record-proceedings-2017-03-24.html

26. Health Canada. Drug products. Accessed March 1, 2021. https://www.canada.ca/en/health-canada/services/drugs-health-products/drug-products/drug-product-database.html

27. Moore RA, Derry S, Aldington D, Wiffen PJ. Single dose oral analgesics for acute postoperative pain in adults - an overview of Cochrane reviews. *Cochrane Database Syst Rev*. 2015;2015(9):Cd008659. doi:10.1002/14651858.CD008659.pub3

28. Gaskell H, Derry S, Wiffen PJ, Moore RA. Single dose oral ketoprofen or dexketoprofen for acute postoperative pain in adults. *Cochrane Database Syst Rev*. 2017;5(5):Cd007355. doi:10.1002/14651858.CD007355.pub3

29. Moore A, Derry S, Eccleston C, Kalso E. Expect analgesic failure; pursue analgesic success. *Bmj*. 2013;346:f2690. doi:10.1136/bmj.f2690

30. World Health Organization. *International classification of functioning, disability and health: ICF*. 2001. Accessed March 1, 2021.

31. Fiore JF, Jr., Figueiredo S, Balvardi S, et al. How Do We Value Postoperative Recovery?: A Systematic Review of the Measurement Properties of Patient-reported Outcomes After Abdominal Surgery. *Ann Surg*. 2018;267(4):656-669. doi:10.1097/sla.0000000000002415

32. Chou R, Gordon DB, de Leon-Casasola OA, et al. Management of Postoperative Pain: A Clinical Practice Guideline From the American Pain Society, the American Society of Regional Anesthesia and Pain Medicine, and the American Society of Anesthesiologists' Committee on Regional Anesthesia, Executive Committee, and Administrative Council. *J Pain*. 2016;17(2):131-57. doi:10.1016/j.jpain.2015.12.008

33. Abola RE, Bennett-Guerrero E, Kent ML, et al. American Society for Enhanced Recovery and Perioperative Quality Initiative Joint Consensus Statement on Patient-Reported Outcomes in an Enhanced Recovery Pathway. *Anesth Analg*. 2018;126(6):1874-1882. doi:10.1213/ane.0000000000002758

34. Jammer I, Wickboldt N, Sander M, et al. Standards for definitions and use of outcome measures for clinical effectiveness research in perioperative medicine: European Perioperative Clinical Outcome (EPCO) definitions: a statement from the ESA-ESICM joint taskforce on perioperative outcome measures. *Eur J Anaesthesiol*. 2015;32(2):88-105. doi:10.1097/eja.0000000000000118

35. Hobart JC, Cano SJ, Zajicek JP, Thompson AJ. Rating scales as outcome measures for clinical trials in neurology: problems, solutions, and recommendations. *Lancet Neurol*. 2007;6(12):1094-105. doi:10.1016/s1474-4422(07)70290-9

36. Keller S, Bann CM, Dodd SL, Schein J, Mendoza TR, Cleeland CS. Validity of the brief pain inventory for use in documenting the outcomes of patients with noncancer pain. *Clin J Pain*. 2004;20(5):309-18. doi:10.1097/00002508-200409000-00005

37. Hah J, Mackey SC, Schmidt P, et al. Effect of Perioperative Gabapentin on Postoperative Pain Resolution and Opioid Cessation in a Mixed Surgical Cohort: A Randomized Clinical Trial. *JAMA Surg*. 2018;153(4):303-311. doi:10.1001/jamasurg.2017.4915

38. Krebs EE, Gravely A, Nugent S, et al. Effect of Opioid vs Nonopioid Medications on Pain-Related Function in Patients With Chronic Back Pain or Hip or Knee Osteoarthritis Pain: The SPACE Randomized Clinical Trial. *Jama*. 2018;319(9):872-882. doi:10.1001/jama.2018.0899

39. van der Meij E, Anema JR, Huirne JAF, Terwee CB. Using PROMIS for measuring recovery after abdominal surgery: a pilot study. *BMC Health Serv Res*. 2018;18(1):128. doi:10.1186/s12913-018-2929-9

40. Chan KS, Chen WH, Gan TJ, et al. Development and validation of a composite score based on clinically meaningful events for the opioid-related symptom distress scale. *Qual Life Res*. 2009;18(10):1331-40. doi:10.1007/s11136-009-9547-2

41. Knisely JS, Wunsch MJ, Cropsey KL, Campbell ED. Prescription Opioid Misuse Index: a brief questionnaire to assess misuse. *J Subst Abuse Treat*. 2008;35(4):380-6. doi:10.1016/j.jsat.2008.02.001

42. Dindo D, Demartines N, Clavien PA. Classification of surgical complications: a new proposal with evaluation in a cohort of 6336 patients and results of a survey. *Ann Surg*. 2004;240(2):205-13. doi:10.1097/01.sla.0000133083.54934.ae

43. Slankamenac K, Graf R, Barkun J, Puhan MA, Clavien PA. The comprehensive complication index: a novel continuous scale to measure surgical morbidity. *Ann Surg*. 2013;258(1):1-7. doi:10.1097/SLA.0b013e318296c732

44. MedDRA. Medical Dictionary for Regulatory Activities. Accessed March 1, 2021. https://www.meddra.org/

45. Colevas AD, Setser A. The NCI Common Terminology Criteria for Adverse Events (CTCAE) v 3.0 is the new standard for oncology clinical trials. *J Clin Oncol*. 2004;22(14):6098-6098.

46. World Health Organization. The use of the WHO-UMC system for standardised case causality assessment. Accessed March 1, 2021. http://www.who.int/medicines/areas/quality_safety/safety_efficacy/WHOcausality_assessment.pdf

47. Boscariol R, Gilron I, Orr E. Chronobiological characteristics of postoperative pain: diurnal variation of both static and dynamic pain and effects of analgesic therapy. *Can J Anaesth*. 2007;54(9):696-704. doi:10.1007/bf03026866

48. Suffeda A, Meissner W, Rosendahl J, Guntinas-Lichius O. Influence of depression, catastrophizing, anxiety, and resilience on postoperative pain at the first day after otolaryngological surgery: A prospective single center cohort observational study. *Medicine (Baltimore)*. 2016;95(28):e4256. doi:10.1097/md.0000000000004256

49. Sullivan MJL, Bishop SR, Pivik J. The pain catastrophizing scale: development and validation. *Psychol Assess*. 1995;7(4):524-532.

50. McCracken LM, Dhingra L. A short version of the Pain Anxiety Symptoms Scale (PASS-20): preliminary development and validity. *Pain Res Manag*. 2002;7(1):45-50. doi:10.1155/2002/517163

51. Akbik H, Butler SF, Budman SH, Fernandez K, Katz NP, Jamison RN. Validation and clinical application of the Screener and Opioid Assessment for Patients with Pain (SOAPP). *J Pain Symptom Manage*. 2006;32(3):287-93. doi:10.1016/j.jpainsymman.2006.03.010

52. Eldridge SM, Chan CL, Campbell MJ, et al. CONSORT 2010 statement: extension to randomised pilot and feasibility trials. *Bmj*. 2016;355:i5239. doi:10.1136/bmj.i5239

53. Teare MD, Dimairo M, Shephard N, Hayman A, Whitehead A, Walters SJ. Sample size requirements to estimate key design parameters from external pilot randomised controlled trials: a simulation study. *Trials*. 2014;15:264. doi:10.1186/1745-6215-15-264

54. Viechtbauer W, Smits L, Kotz D, et al. A simple formula for the calculation of sample size in pilot studies. *J Clin Epidemiol*. 2015;68(11):1375-9. doi:10.1016/j.jclinepi.2015.04.014

55. Braun V, Clarke V. Using thematic analysis in psychology. *Qualitative Research in Psychology*. 2006;3(2):77–101.

56. Tong A, Sainsbury P, Craig J. Consolidated criteria for reporting qualitative research (COREQ): a 32-item checklist for interviews and focus groups. *Int J Qual Health Care*. 2007;19(6):349-57. doi:10.1093/intqhc/mzm042

57. Luborsky MR, Rubinstein RL. Sampling in Qualitative Research: Rationale, Issues, and Methods. *Res Aging*. 1995;17(1):89-113. doi:10.1177/0164027595171005

58. Kerr C, Nixon A, Wild D. Assessing and demonstrating data saturation in qualitative inquiry supporting patient-reported outcomes research. *Expert Rev Pharmacoecon Outcomes Res*. 2010;10(3):269-81. doi:10.1586/erp.10.30

59. Chang AK, Bijur PE, Esses D, Barnaby DP, Baer J. Effect of a Single Dose of Oral Opioid and Nonopioid Analgesics on Acute Extremity Pain in the Emergency Department: A Randomized Clinical Trial. *Jama*. 2017;318(17):1661-1667. doi:10.1001/jama.2017.16190

60. Marquez-Lara A, Hutchinson ID, Nuñez F, Jr., Smith TL, Miller AN. Nonsteroidal Anti-Inflammatory Drugs and Bone-Healing: A Systematic Review of Research Quality. *JBJS Rev*. 2016;4(3)doi:10.2106/jbjs.Rvw.O.00055

61. Kelley BP, Bennett KG, Chung KC, Kozlow JH. Ibuprofen May Not Increase Bleeding Risk in Plastic Surgery: A Systematic Review and Meta-Analysis. *Plast Reconstr Surg*. 2016;137(4):1309-1316. doi:10.1097/prs.0000000000002027

62. Tanne J. Paracetamol causes most liver failure in UK and US. *BMJ*. 2006;332(7542):628-628.

63. Health Canada. About the medical dictionary for regulatory activities. Accessed March 1, 2021. https://www.canada.ca/en/health-canada/services/drugs-health-products/medeffect-canada/adverse-reaction-database/about-medical-dictionary-regulatory-activities-canada-vigilance-adverse-reaction-online-database.html
