# Supplementary material for: Feasibility of Prospectively Comparing Opioid Analgesia With Opioid-Free Analgesia After Outpatient General Surgery: A Pilot Randomized Clinical Trial
Source: JAMA Netw Open. 2022 Jul 18;5(7):e2221430. doi: 10.1001/jamanetworkopen.2022.21430 (PMC9294998; doi:10.1001/jamanetworkopen.2022.21430)
Supplement: Supplement 2. — eFigure 1. Postoperative Analgesia Regimens for Opioid Analgesia (OA) Group eFigure 2. Postoperative Analgesia Regimens for Opioid-free Analgesia (OFA) Group eTable 1. Screening Measures: Constructs Targeted, Corresponding Measures (or Sources of Data), and Description eTable 2. Outcome Measures: Constructs Targeted, Corresponding Measures (or Sources of Data), and Description eTable 3. Relevant Baseline and Operative Characteristics of Eligible Patients Who Consented and Did Not Consent to Participation eTable 4. Comparison Between the Outcome Assessors’ Guesses About Allocation (Opioid vs Opioid-free Analgesia) and Actual Allocation at Postoperative Week 4 After Surgery eFigure 3. Between-group Differences in the PROMIS-29 Domains T-scores eFigure 4. Subgroup Analysis of Brief Pain Inventory in Patients Undergoing Abdominal Surgery eFigure 5. Subgroup Analysis of Brief Pain Inventory in Patients Undergoing Breast Surgery eFigure 6. Subgroup Analysis of PROMIS-29 Domains T-scores in Patients Undergoing Abdominal Surgery eFigure 7. Subgroup Analysis of PROMIS-29 Domains T-scores in Patients Undergoing Breast Surgery eTable 5. Postoperative Pain Management Regimens eTable 6. Adverse Events Identified via the Perioperative Opioid-Related Symptom Distress Scale eTable 7. Patient-reported Postoperative Health Issues (Classified Using MedDRA) eReferences [file jamanetwopen-e2221430-s002.pdf]

# Supplementary Online Content

Do U, El-Kefraoui C, Pook M, et al; McGill Better Opioid Prescribing Collaboration. Feasibility of prospectively comparing opioid analgesia with opioid-free analgesia after outpatient general surgery: a pilot randomized clinical trial. *JAMA Netw Open*. 2022;5(7):e2221430. doi:10.1001/jamanetworkopen.2022.21430

**eFigure 1.** Postoperative Analgesia Regimens for Opioid Analgesia (OA) Group

**eFigure 2.** Postoperative Analgesia Regimens for Opioid-free Analgesia (OFA) Group

**eTable 1.** Screening Measures: Constructs Targeted, Corresponding Measures (or Sources of Data), and Description

**eTable 2.** Outcome Measures: Constructs Targeted, Corresponding Measures (or Sources of Data), and Description

**eTable 3.** Relevant Baseline and Operative Characteristics of Eligible Patients Who Consented and Did Not Consent to Participation

**eTable 4.** Comparison Between the Outcome Assessors' Guesses About Allocation (Opioid vs Opioid-free Analgesia) and Actual Allocation at Postoperative Week 4 After Surgery

**eFigure 3.** Between-group Differences in the PROMIS-29 Domains T-scores

**eFigure 4.** Subgroup Analysis of Brief Pain Inventory in Patients Undergoing Abdominal Surgery

**eFigure 5.** Subgroup Analysis of Brief Pain Inventory in Patients Undergoing Breast Surgery

**eFigure 6.** Subgroup Analysis of PROMIS-29 Domains T-scores in Patients Undergoing Abdominal Surgery

**eFigure 7.** Subgroup Analysis of PROMIS-29 Domains T-scores in Patients Undergoing Breast Surgery

**eTable 5.** Postoperative Pain Management Regimens

**eTable 6.** Adverse Events Identified via the Perioperative Opioid-Related Symptom Distress Scale

**eTable 7.** Patient-reported Postoperative Health Issues (Classified Using MedDRA)

**eReferences**

This supplementary material has been provided by the authors to give readers additional information about their work.

© 2022 Do U et al. *JAMA Network Open*.

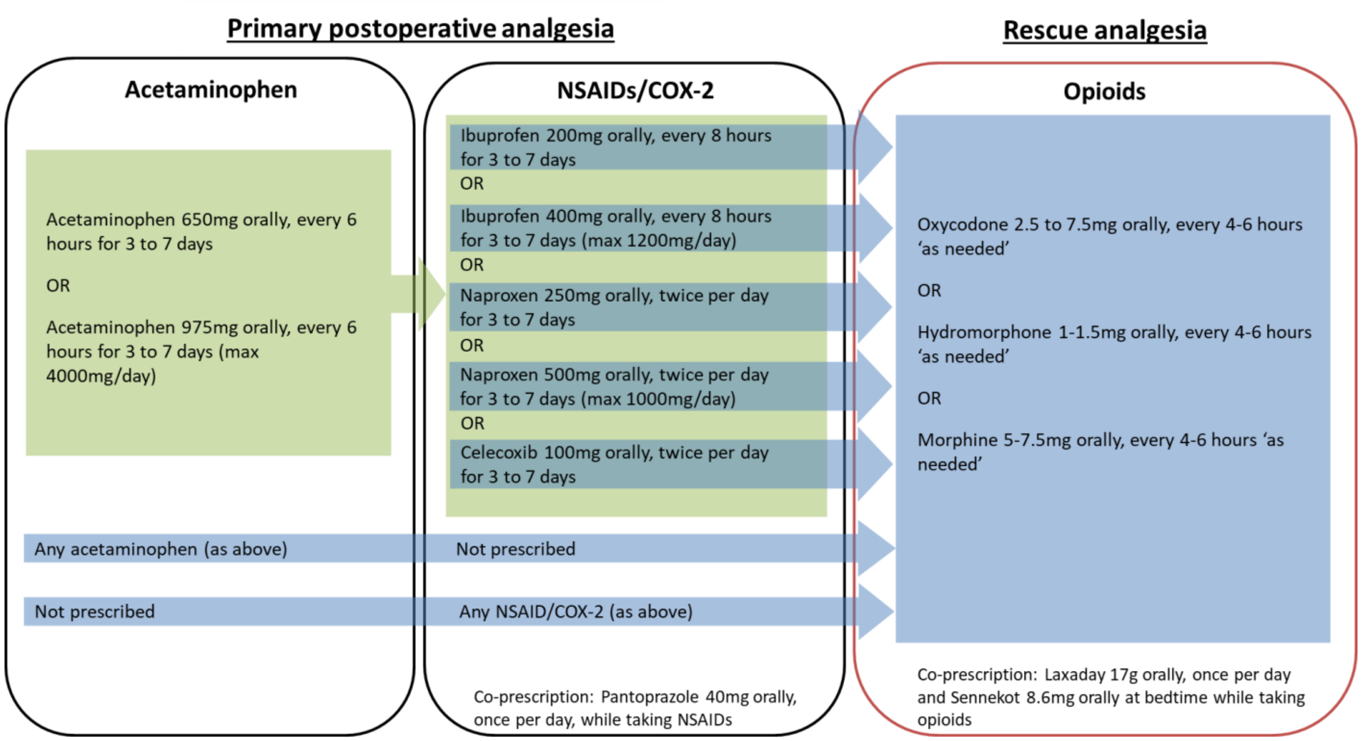


**eFigure 1. Postoperative analgesia regimens for Opioid Analgesia (OA) group**

© 2022 Do U et al. *JAMA Network Open*.

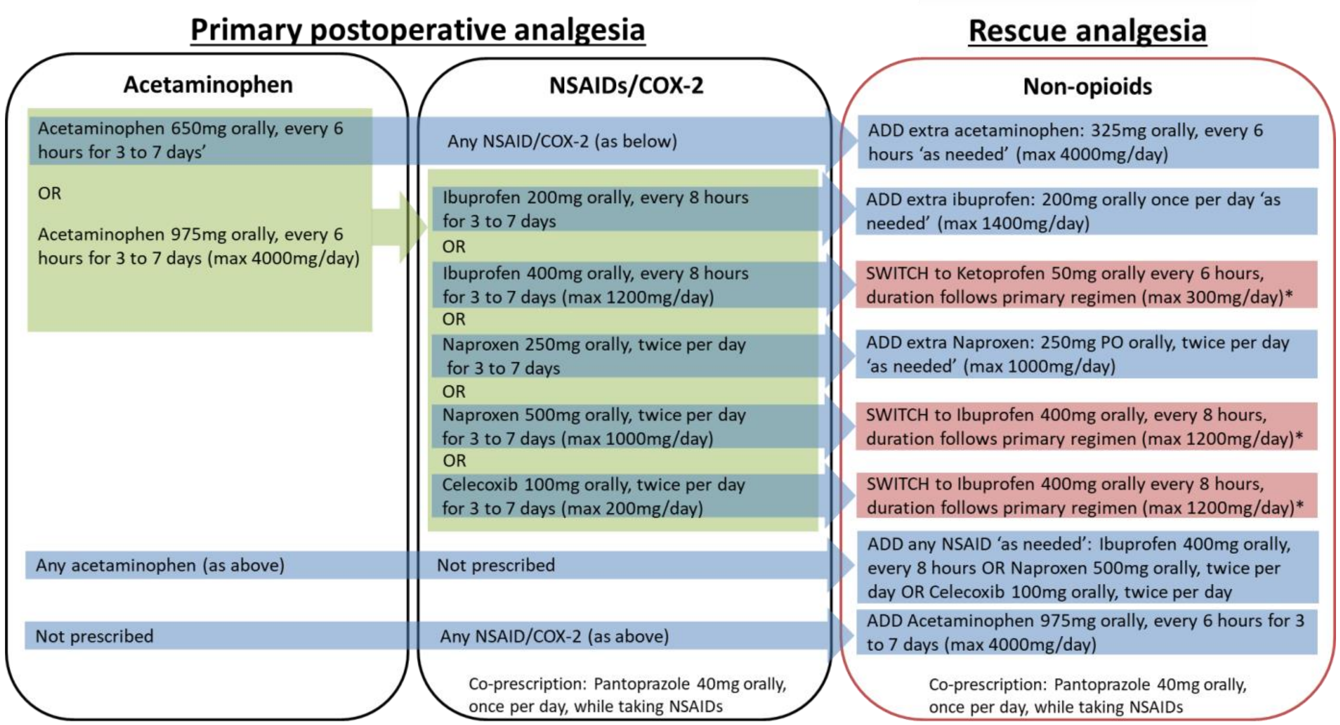


**eFigure 2. Postoperative analgesia regimens for Opioid-Free Analgesia (OFA) group**

*Drug switching informed by single-dose efficacy evidence[1,2] targeting individual variances in analgesia Response [3]. Ketoprofen is not routinely used as primary analgesia and will only be used as a rescue.

© 2022 Do U et al. *JAMA Network Open*.

**eTable 1. Screening measures: Constructs targeted, corresponding measures (or sources of data), and description**

| Target construct | Measure/data source | Description |
|---|---|---|
| Demographic and operative information | Data obtained from electronic medical records | Patient demographics and information relevant to the surgical procedure was obtained from electronic medical records. This included: age, sex, gender, BMI, diagnosis, American Society of Anesthesiologists (ASA) score, surgery performed, technical details, anesthesia information (i.e., use of local infiltrations, blocks and other adjuncts), surgery duration, transfusion requirements, intraoperative and early postoperative complications. |
| Pain Catastrophizing | Pain Catastrophizing Scale[4,5] | This 13-item questionnaire (5-point scale, 0= “not at all”, 4= “all the time”) aims to quantify an individual's tendency to magnify the threat value of pain and to feel helpless in its presence. The recall period is not specific (‘when you are experiencing pain…’). Scoring algorithms provide a total pain catastrophizing score (range 0-52, best-worst), as well as subscale scores (rumination, magnification and helplessness). This questionnaire was administered only preoperatively. |
| Risk for opioid-related aberrant behaviors | Screener and Opioid Assessment for Patients with Pain (SOAPP) Version 1.0-SF[6] | This 5-item self-reported questionnaire was designed to predict aberrant medication-related behaviors among pain patients considered for opioid therapy. Questions focus on history of substance abuse, legal problems, craving medication, heavy smoking, and mood swings. A score of 4 or above indicate high risk of opioid abuse after prescription. This questionnaire was administered only preoperatively. |
| Preferred treatment group | Author-generated question | Patients were asked to state their preferred treatment group using an author-generated question (response options: pain treatment using opioids/pain treatment not using opioids/no preference). Patients responded to this question only preoperatively. |
| Expectation for treatment effectiveness | Author-generated question | Using an author-generated question, patients were asked to state their expectation of treatment effectiveness for pain treatment using opioids and not using opioids (response options: not effective/somewhat effective/very effective). Patients responded to this question only preoperatively. |

BMI = Body Mass Index.

© 2022 Do U et al. *JAMA Network Open*.

**eTable 2. Outcome measures: Constructs targeted, corresponding measures (or sources of data), and description**

| Target construct | Measure/data source | Description |
|---|---|---|
| Pain intensity<br>Pain interference | Brief Pain Inventory Short-Form[7-9] | This is a 9-item questionnaire that addresses pain severity (11-point scale, 0="no pain", 10= "worst pain imaginable") in the last 24 hours. The questionnaire also inquires about pain location, impact of pain on daily function, pain medications (types and amount) and experienced of pain relief. Pain intensity score was calculated as the average of pain at its "worst", "least", "average", and "now" (current pain). Pain interference score was calculated as the average of 7 items: general activity, walking, work, mood, enjoyment of life, relations with others, and sleep. This questionnaire was administered preoperatively (also as a screening measure), on POD 1 to 7 and at 2, 3 and 4 weeks after surgery. Modifications: (1) the diagram indicating pain location was excluded (patients were specifically inquired about pain around the surgical incision(s)) and (2) The item inquiring about the use of specific pain medications, which may unblind assessors to group allocation, was excluded. |
| Time to stopping pain medication | Brief Pain Inventory Short-Form[8] | The time to the first report of stopping the use of pain medication was calculated based on information obtained via treatment adherence telephone follow-ups (assessment not blinded). For follow-ups on POD 1 to POD 7, the time to stopping pain medication was calculated based on the first of two consecutive reports of 'no pain medication'. If pain treatment continued beyond POD 7, patients were asked to recall the last day of pain medication use at 2, 3 and 4 weeks after surgery, as appropriate. |
| Physical function<br>Anxiety<br>Depression<br>Fatigue<br>Sleep disturbance<br>Social roles and activities<br>Pain intensity<br>Pain interference | PROMIS-29 Physical function<br>PROMIS-29 Anxiety<br>PROMIS-29 Depression<br>PROMIS-29 Fatigue<br>PROMIS-29 Sleep disturbance<br>PROMIS-29 Social roles and activities<br>PROMIS-29 Pain intensity<br>PROMIS-29 Pain interference[9-11] | This generic health-related quality of life survey, derived from the US National Institutes of Health (NIH) PROMIS item bank, assesses 7 domains of health (physical function, anxiety, depression, fatigue, sleep disturbance, ability to participate in social roles and activities, and pain interference). It contains 29 items, including four items from each primary domain (5-point scale, ranging from 1 to 5, with different response options for different domains) plus a single item for pain intensity rating (11-point rating scale, where 0=no pain and 10=worst imaginable pain). Recall periods vary between domains (7 days or non-specific). Higher scores indicate more of the particular scale's domains, which may represent a desirable outcome (e.g., higher scores for the Physical Function scale represent better function) or an undesirable outcome (e.g., higher scores on the Depression scale indicate more depressive symptoms). Scoring is based on item-response theory. Raw scores are calculated separately for each domain and expressed as T-scores, representing a standardized score with a mean of 50 (corresponding to the mean score in the US general population) and a standard deviation (SD) of 10. This questionnaire was administered preoperatively (also as a screening measure), on POD 7 and at 2, 3 and 4 weeks after surgery. |
| Opioid side effects | Perioperative Opioid-Related Symptom Distress Scale[12] | This 10-item questionnaire measures symptom distress due to common adverse effects experienced by patients who receive opioids to relieve postoperative pain (fatigue, drowsiness, inability to concentrate, confusion, nausea, dizziness, constipation, itching, difficulty with urination, and retching/vomiting). These adverse effects are assessed across 3 distress dimensions: frequency ('rarely' to 'almost constantly'), severity ('slight' to 'very severe') and degree of bother ('not at all' to 'very much'). The recall period is 24 hours. We calculated a composite score based on clinically meaningful events as published by Chan et al, as well individual scores for each |

© 2022 Do U et al. *JAMA Network Open*.

| | | |
|---|---|---|
| | | symptom. This questionnaire was administered on POD 7 and at 2, 3 and 4 weeks after surgery. |
| Opioid misuse | Prescription Opioid Abuse Index[13] | This 6-item questionnaire includes questions regarding excessive dose, frequency of use, need for early refills, feeling high from the medication, taking the medication due to stress and obtaining prescriptions from multiple physicians. An affirmative answer to more than one question correctly classified an individual as an opioid misuser. This questionnaire was administered at 4 weeks after surgery. |
| 30-day postoperative complications | Data obtained from electronic medical records | Data regarding postoperative complications was obtained from medical records and graded by severity using the Clavien-Dindo classification.[42] This system grades complications according to the therapy needed for treatment (grades I to IV, best to worse). Complications within 30 days after surgery were recorded. In addition, the Comprehensive Complication Index (CCI) was generated for each patient to summarize the complete spectrum of postoperative complications and their severity in a single score ranging from 0 to 100 (best to worse) [14]. |
| 30-day unplanned healthcare utilization | Data on obtained from the electronic medical records and verified with patients via phone follow-up | Unplanned healthcare utilization (ED visits and unplanned hospital readmissions) within 30 days after surgery was extracted from the electronic medical records and verified with patients via phone follow-up. Patients were also be inquired about ED visits and admissions to non-MUHC sites, as well as emergency visits to outpatient care providers (i.e., family doctor, walk in-clinics, surgery clinic). |
| 30-day adverse drug events | Data obtained from spontaneous patient reporting and electronic medical records | Data regarding adverse drug events was obtained from spontaneous patient reporting (Trigger question "Did you have any significant medical problem related or unrelated to your surgery since the last study assessment?") and from data reported by clinicians in electronic medical records. Two independent clinicians, blinded to treatment allocation, will code adverse event data using the MedDRA coding dictionary [15].Disagreements regarding coding, were resolved by consensus. Adverse drug events were monitored up to 30 days after surgery. |
| Prolonged opioid use (3-month follow up) | Data obtained via the Dossier Santé Québec | Requirement for extra opioid prescriptions was monitored for 3 months via the Dossier Santé Québec, which is a province-wide electronic health information system that includes drug prescriptions received in hospitals and outpatient settings. The percentage of patients receiving opioids was calculated weekly in the first month, and then monthly until the 3rd month after surgery. |
| Recovery from surgery | Author-generated question | Patients were asked whether they consider themselves to be completely recovered from the surgery (response options: yes/no). Patients responded to this question at 4 weeks after surgery. Time to complete recovery was calculated based on the difference (in days) between date perceived of feeling fully recovered and surgery date. |
| Return to work or normal activities | Author-generated question | Patients were asked whether they have returned to work (any vocational activity, paid or not paid) or, if unemployed or retired, if they returned to pre-operative levels of activity (response options: yes/no). Patients responded to this question at 4 weeks after surgery. |
| Overall impression of treatment effectiveness | Author-generated question | Patients were asked to state their overall impression about the effectiveness of the pain treatment that they are receiving (response options: not effective/somewhat effective/very effective). Patients responded to this question at each postoperative time-point, until they reported having stopped using pain medication. |
| Overall satisfaction with the pain treatment received | Author-generated question | Patients were asked to rate their overall satisfaction with the pain treatment that they received (response options: very dissatisfied /dissatisfied/satisfied/very satisfied). Patients responded to this question when they report having stopped using pain medication or at 4 weeks after surgery, whichever comes first. |

© 2022 Do U et al. *JAMA Network Open*.

WHO = World Health Organization, MedDRA = Medical Dictionary for Regulatory Activities, CTCAE = Common Terminology Criteria for Adverse Events, WHO/UMC = World Health Organization/Uppsala Monitoring Centre, PROMIS = Patient-Reported Outcomes Measurement Information System.

© 2022 Do U et al. *JAMA Network Open*.

**eTable 3. Relevant baseline and operative characteristics of eligible patients who consented and did not consent to participation**

| | Eligible and consented (n=76) | Eligible and did not consent (n=70) | p-value |
|---|---|---|---|
| Age, mean (SD), y | 55.5 (14.5) | 57.9 (14.6) | 0.32 |
| ≥ 75 years old | 5 (7) | 6 (9) | 0.65 |
| Female | 50 (66) | 41 (59) | 0.37 |
| BMI, mean (SD)[a] | 27.6 (7.0)[b] | 26.6 (5.1)[b] | 0.36 |
| ≥ 30.0 | 18 (25)[b] | 11 (16)[b] | 0.18 |
| Physical status (ASA) | | | |
| I | 15 (20) | 11 (16) | 0.58 |
| II | 53 (70) | 53 (78) | 0.26 |
| III | 8 (10) | 4 (6) | 0.31 |
| Current smoker | 13 (18)[c] | 11 (16) | 0.77 |
| Current at-risk alcohol use[e] | 5 (7) | 8 (11) | 0.33 |
| Previous surgery | 65 (86) | 53 (76)[d] | 0.18 |
| Abdominal surgery | 40 (53) | 35 (50) | 0.75 |
| Laparoscopic appendectomy | 1 (1) | 1 (1) | 0.95 |
| Laparoscopic cholecystectomy | 9 (12) | 8 (11)[f] | 0.94 |
| Laparoscopic inguinal hernia repair | 9 (12)[f] | 6 (9) | 0.52 |
| Laparoscopic incisional hernia repair | 0 (0) | 1 (1) | 0.30 |
| Open inguinal hernia repair | 17 (22) | 16 (23) | 0.94 |
| Open umbilical hernia repair | 3 (4) | 3 (4) | 0.92 |
| Open incisional hernia repair | 1 (1) | 0 (0) | 0.34 |
| Breast surgery | 36 (47) | 35 (50) | 0.75 |
| Partial mastectomy | 14 (18) | 13 (19) | 0.98 |
| Partial mastectomy with sentinel node biopsy | 11 (14) | 6 (9) | 0.27 |
| Partial mastectomy with axillary node dissection | 6 (8) | 9 (13) | 0.33 |
| Partial mastectomy with reconstruction | 0 (0) | 1 (1) | 0.30 |
| Partial mastectomy with sentinel node biopsy and reconstruction | 1 (1) | 1 (1) | 0.95 |
| Total mastectomy with sentinel node biopsy | 2 (3) | 1 (1) | 0.61 |
| Total mastectomy with sentinel node biopsy and reconstruction | 1 (1) | 2 (3) | 0.51 |
| Total mastectomy with axillary node dissection and reconstruction | 1 (1) | 2 (3) | 0.51 |
| Duration of surgery (minutes) | 91±45 | 94±46[c] | 0.68 |
| Received intraoperative regional analgesia | | | |
| Peripheral nerve block | 11 (14) | 14 (20) | 0.38 |
| Local infiltration | 57 (75) | 56 (80) | 0.47 |

Data are n (%), median (IQR) or mean±SD. Continuous variables compared using Mann–Whitney U test and categorical variables compared using Chi-square test. BMI=body mass index; ASA=American Society of Anesthesiologists

© 2022 Do U et al. *JAMA Network Open*.

[a] Calculated as weight in kilograms divided by square of heights in meters.
[b] Missing data for 3 patients.
[c] Missing data for 2 patients.
[d] Missing data for 1 patient.
[e] Alcohol consumption above Canada's Low-Risk Alcohol Drinking Guidelines (2017) for men (>15 standard drinks per week) and women (>10 standard drinks per week) [16].
[f] Includes one patient who had an open umbilical hernia repair during the same procedure.

© 2022 Do U et al. *JAMA Network Open*.

**eTable 4. Comparison between the outcome assessors' guesses about allocation (opioid vs opioid-free analgesia) and actual allocation at postoperative week 4 after surgery**

| | Opioid analgesia | Opioid-free analgesia |
|---|---|---|
| Correct guess | 19/39 (49) | 18/37 (49) |
| Incorrect guess | 20/39 (51) | 19/37 (51) |

Data are number of correct guesses/total number of guesses (%).

© 2022 Do U et al. *JAMA Network Open*.

## A. Physical functioning

| Timepoint | Opioid-free analgesia Mean | SD | Median | IQR | Range | Opioid analgesia Mean | SD | Median | IQR | Range | Mean difference [95% CI] |
|---|---|---|---|---|---|---|---|---|---|---|---|
| Baseline | 50.8 | 8.6 | 57.0 | [46.1 - 57.0] | [30.6 - 57.0] | 50.2 | 8.3 | 57.0 | [43.3 - 57.0] | [31.1 - 57.0] | 0.60 [ -3.30, 4.40] |
| POW 1 | 44.1 | 9.1 | 44.6 | [38.4 - 47.8] | [22.6 - 57.0] | 44.9 | 8.3 | 44.6 | [39.8 - 47.8] | [27.9 - 57.0] | -0.80 [ -4.80, 3.10] |
| POW 2 | 47.9 | 8.1 | 47.8 | [42.9 - 57.0] | [27.7 - 57.0] | 47.2 | 7.5 | 45.7 | [41.6 - 57.0] | [32.5 - 57.0] | 0.60 [ -2.90, 4.20] |
| POW 3 | 50.6 | 7.3 | 56.3 | [45.1 - 57.0] | [35.5 - 57.0] | 49.6 | 7.7 | 48.1 | [42.9 - 57.0] | [34.5 - 57.0] | 1.00 [ -2.50, 4.50] |
| POW 4 | 51.5 | 7.2 | 57.0 | [46.1 - 57.0] | [37.2 - 57.0] | 52.1 | 7.0 | 57.0 | [45.1 - 57.0] | [36.9 - 57.0] | -0.60 [ -3.90, 2.70] |

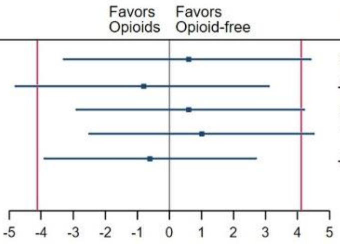


## B. Social participation

| Timepoint | Opioid-free analgesia Mean | SD | Median | IQR | Range | Opioid analgesia Mean | SD | Median | IQR | Range | Mean difference [95% CI] |
|---|---|---|---|---|---|---|---|---|---|---|---|
| Baseline | 52.9 | 8.7 | 52.2 | [46.1 - 58.5] | [32.9 - 64.2] | 55.0 | 9.4 | 57.5 | [48.2 - 64.2] | [38.5 - 64.2] | -2.10 [ -6.20, 2.10] |
| POW 1 | 47.3 | 10.4 | 46.1 | [42.2 - 51.8] | [27.5 - 64.2] | 49.6 | 9.6 | 50.2 | [41.8 - 58.1] | [31.6 - 64.2] | -2.30 [ -6.80, 2.30] |
| POW 2 | 54.4 | 8.3 | 55.5 | [49.5 - 64.2] | [37.2 - 64.2] | 54.5 | 8.8 | 55.9 | [44.8 - 64.2] | [38.6 - 64.2] | -0.10 [ -4.00, 3.80] |
| POW 3 | 55.6 | 8.6 | 57.5 | [48.3 - 64.2] | [37.4 - 64.2] | 56.5 | 8.0 | 57.5 | [50.1 - 64.2] | [37.2 - 64.2] | -0.90 [ -4.70, 3.00] |
| POW 4 | 56.6 | 8.4 | 58.4 | [51.8 - 64.2] | [37.8 - 64.2] | 57.2 | 8.4 | 58.5 | [51.6 - 64.2] | [35.1 - 64.2] | -0.60 [ -4.50, 3.40] |

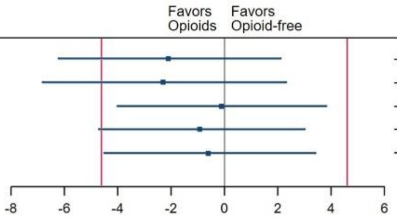


## C. Anxiety

| Timepoint | Opioid-free analgesia Mean | SD | Median | IQR | Range | Opioid analgesia Mean | SD | Median | IQR | Range | Mean difference [95% CI] |
|---|---|---|---|---|---|---|---|---|---|---|---|
| Baseline | 53.0 | 8.5 | 53.6 | [48.1 - 61.3] | [40.3 - 69.5] | 53.3 | 9.9 | 56.0 | [40.3 - 60.0] | [40.3 - 71.4] | -0.30 [ -4.50, 4.00] |
| POW 1 | 48.6 | 8.0 | 49.0 | [40.3 - 55.8] | [40.3 - 68.1] | 46.4 | 8.4 | 40.3 | [40.3 - 55.3] | [40.3 - 65.1] | 2.20 [ -1.60, 5.90] |
| POW 2 | 44.7 | 6.9 | 40.3 | [40.3 - 50.4] | [40.3 - 63.5] | 45.9 | 8.1 | 40.3 | [40.3 - 51.4] | [40.3 - 65.2] | -1.20 [ -4.60, 2.30] |
| POW 3 | 43.2 | 5.6 | 40.3 | [40.3 - 40.7] | [40.3 - 58.0] | 44.4 | 6.4 | 40.3 | [40.3 - 48.2] | [40.3 - 59.7] | -1.20 [ -4.00, 1.60] |
| POW 4 | 44.4 | 6.7 | 40.3 | [40.3 - 48.1] | [40.3 - 57.9] | 44.5 | 6.7 | 40.3 | [40.3 - 48.2] | [40.3 - 63.5] | -0.10 [ -3.20, 3.10] |

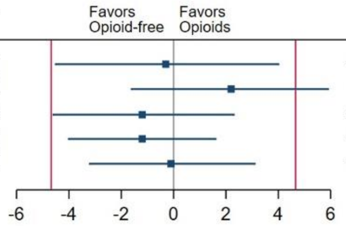


## D. Depression

| Timepoint | Opioid-free analgesia Mean | SD | Median | IQR | Range | Opioid analgesia Mean | SD | Median | IQR | Range | Mean difference [95% CI] |
|---|---|---|---|---|---|---|---|---|---|---|---|
| Baseline | 48.0 | 8.2 | 41.0 | [41.0 - 55.9] | [41.0 - 69.5] | 47.1 | 8.7 | 41.0 | [41.0 - 54.1] | [41.0 - 67.5] | 0.90 [ -3.00, 4.70] |
| POW 1 | 46.2 | 7.2 | 41.0 | [41.0 - 49.3] | [41.0 - 62.2] | 45.6 | 6.8 | 41.0 | [41.0 - 49.1] | [41.0 - 60.6] | 0.60 [ -2.60, 3.80] |
| POW 2 | 43.6 | 5.8 | 41.0 | [41.0 - 41.0] | [41.0 - 62.2] | 45.1 | 6.7 | 41.0 | [41.0 - 49.8] | [41.0 - 62.2] | -1.50 [ -4.30, 1.40] |
| POW 3 | 43.7 | 5.0 | 41.0 | [41.0 - 49.8] | [41.0 - 57.5] | 44.7 | 6.4 | 41.0 | [41.0 - 49.8] | [41.0 - 60.6] | -1.00 [ -3.70, 1.70] |
| POW 4 | 44.3 | 6.0 | 41.0 | [41.0 - 48.9] | [41.0 - 58.7] | 44.1 | 5.3 | 41.0 | [41.0 - 48.9] | [41.0 - 57.5] | 0.20 [ -2.40, 2.90] |

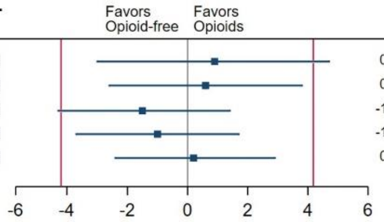


## E. Pain interference

| Timepoint | Opioid-free analgesia Mean | SD | Median | IQR | Range | Opioid analgesia Mean | SD | Median | IQR | Range | Mean difference [95% CI] |
|---|---|---|---|---|---|---|---|---|---|---|---|
| Baseline | 48.4 | 8.9 | 41.6 | [41.6 - 68.9] | [41.6 - 68.9] | 49.1 | 9.0 | 41.6 | [41.6 - 65.5] | [41.6 - 65.5] | -0.60 [ -4.70, 3.50] |
| POW 1 | 55.5 | 9.1 | 55.7 | [52.2 - 61.3] | [41.6 - 75.6] | 55.7 | 8.3 | 55.7 | [51.7 - 61.3] | [41.6 - 71.3] | -0.20 [ -4.20, 3.70] |
| POW 2 | 49.9 | 7.1 | 52.4 | [41.6 - 54.9] | [41.6 - 61.3] | 50.5 | 8.9 | 51.7 | [41.6 - 57.5] | [41.6 - 67.8] | -0.60 [ -4.30, 3.10] |
| POW 3 | 46.5 | 7.3 | 41.6 | [41.6 - 53.9] | [41.6 - 64.7] | 48.5 | 7.6 | 41.6 | [41.6 - 55.7] | [41.6 - 65.2] | -2.00 [ -5.40, 1.50] |
| POW 4 | 46.7 | 7.1 | 41.6 | [41.6 - 52.4] | [41.6 - 62.3] | 45.9 | 6.8 | 41.6 | [41.6 - 51.7] | [41.6 - 61.1] | 0.90 [ -2.40, 4.10] |

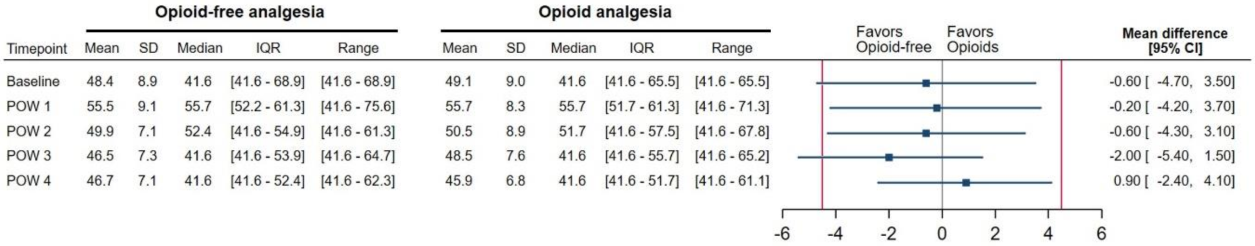


## F. Sleep disturbance

| Timepoint | Opioid-free analgesia Mean | SD | Median | IQR | Range | Opioid analgesia Mean | SD | Median | IQR | Range | Mean difference [95% CI] |
|---|---|---|---|---|---|---|---|---|---|---|---|
| Baseline | 49.3 | 8.6 | 52.1 | [46.4 - 54.5] | [32.0 - 62.1] | 46.8 | 9.8 | 46.4 | [41.2 - 51.4] | [32.0 - 69.4] | 2.50 [ -1.70, 6.70] |
| POW 1 | 47.4 | 9.1 | 46.4 | [42.1 - 52.8] | [32.0 - 69.4] | 47.1 | 8.5 | 46.4 | [41.2 - 53.3] | [32.0 - 63.8] | 0.30 [ -3.70, 4.40] |
| POW 2 | 46.9 | 9.0 | 47.2 | [41.2 - 52.8] | [32.0 - 63.8] | 45.2 | 7.5 | 46.3 | [41.2 - 49.6] | [32.0 - 58.7] | 1.70 [ -2.00, 5.50] |
| POW 3 | 46.1 | 9.3 | 48.3 | [40.0 - 53.3] | [32.0 - 60.6] | 45.0 | 8.3 | 43.8 | [38.4 - 51.1] | [32.0 - 62.0] | 1.10 [ -3.00, 5.20] |
| POW 4 | 44.3 | 9.3 | 45.3 | [32.0 - 49.6] | [32.0 - 63.4] | 43.6 | 8.5 | 44.1 | [36.9 - 51.1] | [32.0 - 61.9] | 0.70 [ -3.50, 4.80] |

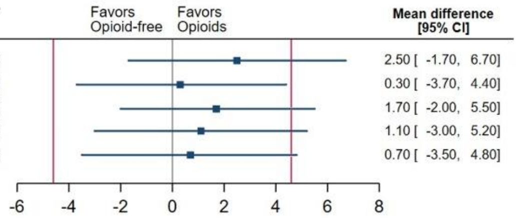


## G. Fatigue

| Timepoint | Opioid-free analgesia Mean | SD | Median | IQR | Range | Opioid analgesia Mean | SD | Median | IQR | Range | Mean difference [95% CI] |
|---|---|---|---|---|---|---|---|---|---|---|---|
| Baseline | 47.7 | 10.0 | 48.6 | [39.7 - 57.1] | [33.7 - 64.7] | 45.9 | 9.5 | 46.0 | [33.7 - 53.2] | [33.7 - 66.6] | 1.80 [ -2.70, 6.20] |
| POW 1 | 50.2 | 11.2 | 48.6 | [43.1 - 57.1] | [33.7 - 75.8] | 48.4 | 9.4 | 48.6 | [43.1 - 53.2] | [33.7 - 71.5] | 1.80 [ -2.90, 6.60] |
| POW 2 | 44.6 | 10.3 | 46.0 | [33.7 - 48.6] | [33.7 - 64.7] | 43.0 | 9.6 | 43.1 | [33.7 - 51.0] | [33.7 - 62.7] | 1.60 [ -2.90, 6.20] |
| POW 3 | 42.4 | 9.9 | 39.8 | [33.7 - 48.6] | [33.7 - 64.7] | 41.8 | 8.2 | 40.6 | [33.7 - 48.6] | [33.7 - 57.1] | 0.60 [ -3.60, 4.80] |
| POW 4 | 42.2 | 9.8 | 36.7 | [33.7 - 48.6] | [33.7 - 64.8] | 40.9 | 7.7 | 39.8 | [33.7 - 48.6] | [33.7 - 55.2] | 1.20 [ -2.90, 5.30] |

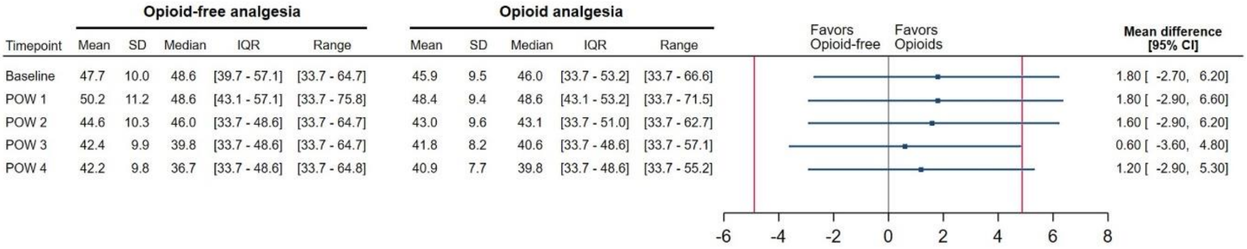


**eFigure 3. Between-group differences in the PROMIS-29 domains T-scores.**

Red lines represent minimal clinically important differences (MCID) (MCIDs were estimated as 0.5 standard deviation at baseline for each domain[17]). Missing follow-up data: POW3 n = 2, POW4 n = 3. Higher scores on physical function and social participation domains indicate desirable outcomes. Higher scores on anxiety, depression, pain interference, sleep disturbance, and fatigue domains indicate undesirable outcomes. Pain intensity scores are not based on the same unit of measure of the other domains (T-scores) and, therefore, are not included in the graph. PROMIS-29 pain intensity data are reported in the table below.

© 2022 Do U et al. *JAMA Network Open*.

## PROMIS-29® pain intensity scores[a]

| | Opioid analgesia (n=39) | | | Opioid-free analgesia (n=37) | | | Between-group difference (95%CI)[b] |
|---|---|---|---|---|---|---|---|
| | Mean (SD) | Median (IQR) | Range (max – min) | Mean (SD) | Median (IQR) | Range (max – min) | |
| Baseline | 2.2 (2.5) | 1 (0-4) | 9 (0-9) | 2.2 (2.6) | 2 (0-3) | 8 (0-8) | 0.1 (-1.1 to 1.2) |
| Postoperative week 1 | 2.5 (1.9) | 2 (1-4) | 7 (0-7) | 2.7 (2.1) | 3 (1-4) | 7 (0-7) | 0.2 (-0.7 to 1.1) |
| Postoperative week 2 | 1.5 (1.7) | 1 (0-2) | 6 (0-6) | 1.2 (1.4) | 1 (0-2) | 6 (0-6) | -0.3 (-1.0 to 0.4) |
| Postoperative week 3 | 0.8 (1.2) | 0 (0-2) | 5 (0-5) | 1.0 (1.4) | 1 (0-1) | 6 (0-6) | 0.2 (-0.5 to 0.8) |
| Postoperative week 4 | 0.7 (1.4) | 0 (0-1) | 7 (0-7) | 0.8 (1.1) | 0 (0-1) | 5 (0-5) | 0.1 (-0.6 to 0.6) |

Data are mean (SD). PROMIS® = Patient-Reported Outcomes Measurement Information System. POW3 OFA n =35; POW4 OFA n = 34 (due to losses of follow-up).
[a] Pain intensity rating is a 11-point rating scale (range, 0-10; 0 = no pain and 10 = worst imaginable pain). Recall period is 7 days.
[b] Between-group difference represents mean difference.

© 2022 Do U et al. *JAMA Network Open*.

## A. Pain intensity

| Timepoint | Opioid-free analgesia Mean | SD | Median | IQR | Range | Opioid analgesia Mean | SD | Median | IQR | Range | Mean difference [95% CI] |
|---|---|---|---|---|---|---|---|---|---|---|---|
| Baseline | 1.4 | 2.4 | 0 | [0 - 2.3] | [0 - 6.8] | 1.3 | 1.9 | 0 | [0 - 2.8] | [0 - 5.0] | 0.20 [ -1.20, 1.50] |
| POD 1 | 3.3 | 1.6 | 3.4 | [2.4 - 4.4] | [0 - 6.3] | 3.2 | 2.0 | 3.8 | [2.0 - 4.8] | [0 - 6.5] | 0.10 [ -1.00, 1.30] |
| POD 2 | 2.8 | 2.1 | 2.5 | [1.5 - 4.6] | [0 - 6.3] | 3.2 | 2.1 | 3.6 | [1.9 - 4.8] | [0 - 7.5] | -0.40 [ -1.70, 1.00] |
| POD 3 | 2.2 | 2.0 | 2.0 | [0.3 - 3.5] | [0 - 6.3] | 2.5 | 2.1 | 2.1 | [0.9 - 4.0] | [0 - 7.0] | -0.20 [ -1.50, 1.00] |
| POD 4 | 1.6 | 1.8 | 1.1 | [0 - 3.0] | [0 - 5.5] | 2.0 | 1.9 | 1.6 | [0 - 3.4] | [0 - 6.3] | -0.30 [ -1.50, 0.90] |
| POD 5 | 1.2 | 1.4 | 0.5 | [0 - 2.5] | [0 - 4.3] | 1.6 | 2.1 | 0.8 | [0 - 2.6] | [0 - 6.8] | -0.40 [ -1.50, 0.80] |
| POD 6 | 1.2 | 1.5 | 0.5 | [0 - 1.9] | [0 - 5.3] | 1.6 | 1.8 | 1.0 | [0 - 3.1] | [0 - 6.0] | -0.40 [ -1.50, 0.60] |
| POD 7 | 0.9 | 1.0 | 0.5 | [0 - 1.8] | [0 - 2.8] | 1.4 | 2.0 | 0.4 | [0 - 2.3] | [0 - 7.3] | -0.50 [ -1.50, 0.50] |
| POW 2 | 0.4 | 0.6 | 0 | [0 - 0.6] | [0 - 2.0] | 1.1 | 1.4 | 0.1 | [0 - 2.1] | [0 - 4.0] | -0.70 [ -1.40, 0.00] |
| POW 3 | 0.5 | 1.0 | 0 | [0 - 0.8] | [0 - 3.0] | 0.8 | 1.2 | 0 | [0 - 1.9] | [0 - 3.5] | -0.30 [ -1.00, 0.40] |
| POW 4 | 0.4 | 1.0 | 0 | [0 - 0] | [0 - 4.0] | 0.8 | 1.8 | 0 | [0 - 1.0] | [0 - 7.0] | -0.40 [ -1.30, 0.50] |

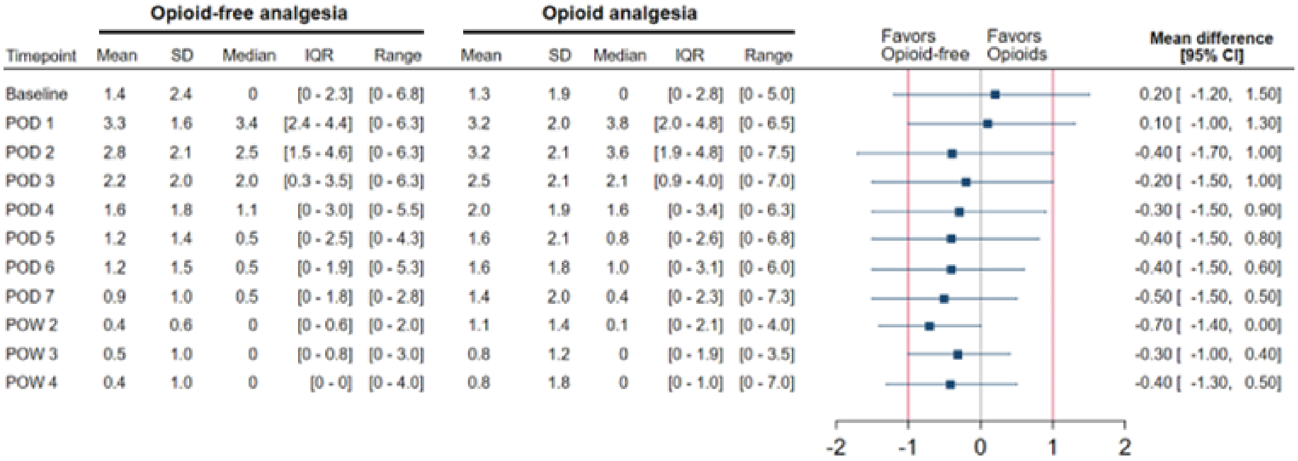


## B. Pain interference

| Timepoint | Opioid-free analgesia Mean | SD | Median | IQR | Range | Opioid analgesia Mean | SD | Median | IQR | Range | Mean difference [95% CI] |
|---|---|---|---|---|---|---|---|---|---|---|---|
| Baseline | 1.1 | 2.1 | 0 | [0 - 0.7] | [0 - 7.0] | 1.2 | 2.1 | 0 | [0 - 2.2] | [0 - 5.9] | -0.20 [ -1.50, 1.20] |
| POD 1 | 4.1 | 1.8 | 4.2 | [3.2 - 5.2] | [0 - 7.1] | 3.9 | 3.0 | 3.6 | [1.6 - 6.5] | [0 - 8.6] | 0.20 [ -1.30, 1.80] |
| POD 2 | 3.0 | 2.2 | 3.4 | [1.1 - 5.0] | [0 - 6.7] | 3.2 | 2.4 | 3.7 | [0.9 - 4.7] | [0 - 8.0] | -0.20 [ -1.60, 1.30] |
| POD 3 | 2.5 | 2.2 | 2.0 | [0.2 - 4.6] | [0 - 6.6] | 2.7 | 2.5 | 2.7 | [0.1 - 4.9] | [0 - 7.0] | -0.20 [ -1.60, 1.30] |
| POD 4 | 1.8 | 2.3 | 0.6 | [0 - 4.1] | [0 - 7.6] | 2.1 | 2.4 | 0.9 | [0 - 4.0] | [0 - 7.9] | -0.30 [ -1.80, 1.20] |
| POD 5 | 1.4 | 2.1 | 0.3 | [0 - 1.6] | [0 - 7.4] | 1.5 | 2.1 | 0.5 | [0 - 2.1] | [0 - 7.3] | -0.10 [ -1.50, 1.20] |
| POD 6 | 1.2 | 1.8 | 0 | [0 - 2.0] | [0 - 6.4] | 1.7 | 2.1 | 0.4 | [0 - 3.4] | [0 - 5.9] | -0.50 [ -1.80, 0.70] |
| POD 7 | 1.5 | 2.4 | 0 | [0 - 2.1] | [0 - 7.3] | 1.4 | 2.1 | 0.4 | [0 - 2.2] | [0 - 6.4] | 0.10 [ -1.30, 1.50] |
| POW 2 | 0.6 | 1.3 | 0 | [0 - 0.6] | [0 - 5.3] | 1.1 | 1.7 | 0.1 | [0 - 1.1] | [0 - 5.6] | -0.50 [ -1.50, 0.50] |
| POW 3 | 0.7 | 1.6 | 0 | [0 - 0] | [0 - 5.9] | 1.0 | 1.9 | 0 | [0 - 0.8] | [0 - 7.0] | -0.30 [ -1.40, 0.90] |
| POW 4 | 0.2 | 0.8 | 0 | [0 - 0] | [0 - 3.7] | 0.4 | 0.8 | 0 | [0 - 0.6] | [0 - 3.1] | -0.20 [ -0.70, 0.30] |

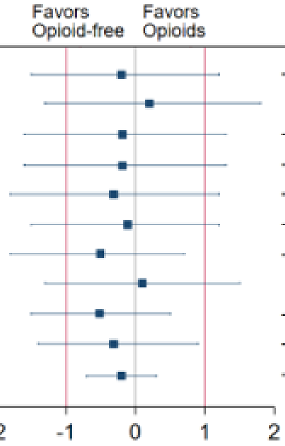


**eFigure 4. Subgroup analysis of Brief Pain Inventory in patients undergoing abdominal surgery. Plots represent between-group differences in the Brief Pain Inventory severity scale (composite of 4 items, score 0-10) and interference scale (composite of 7 items, score 0-10). Red lines represent minimal clinically important differences[18,19]. POW3 OFA n =35; POW4 OFA n = 34 (due to losses of follow-up).**

© 2022 Do U et al. *JAMA Network Open*.

**A. Pain intensity**

| | Opioid-free analgesia | | | | | Opioid analgesia | | | | | |
|---|---|---|---|---|---|---|---|---|---|---|---|
| Timepoint | Mean | SD | Median | IQR | Range | Mean | SD | Median | IQR | Range | Mean difference [95% CI] |
| Baseline | 0.4 | 1.2 | 0 | [0 - 0] | [0 - 4.5] | 0.4 | 1.5 | 0 | [0 - 0] | [0 - 6.5] | -0.10 [ -1.00, 0.90] |
| POD 1 | 2.6 | 1.8 | 2.5 | [1.8 - 4.0] | [0 - 6.5] | 2.4 | 2.4 | 1.5 | [0 - 3.8] | [0 - 8.0] | 0.30 [ -1.20, 1.70] |
| POD 2 | 2.2 | 2.2 | 2.0 | [0 - 3.8] | [0 - 7.3] | 2.1 | 2.4 | 1.0 | [0 - 3.8] | [0 - 7.3] | 0.20 [ -1.40, 1.70] |
| POD 3 | 1.2 | 1.5 | 0.8 | [0 - 2.3] | [0 - 4.3] | 1.7 | 1.9 | 1.0 | [0 - 2.8] | [0 - 5.8] | -0.50 [ -1.70, 0.60] |
| POD 4 | 1.2 | 1.5 | 0.8 | [0 - 2.3] | [0 - 4.3] | 1.3 | 1.6 | 1.0 | [0 - 2.5] | [0 - 4.5] | -0.10 [ -1.20, 0.90] |
| POD 5 | 1.0 | 1.2 | 0.8 | [0 - 2.0] | [0 - 3.5] | 1.0 | 1.3 | 0 | [0 - 1.8] | [0 - 4.3] | 0.00 [ -0.80, 0.90] |
| POD 6 | 0.9 | 1.3 | 0 | [0 - 1.8] | [0 - 4.5] | 0.8 | 1.0 | 0 | [0 - 1.8] | [0 - 3.0] | 0.10 [ -0.70, 0.90] |
| POD 7 | 1.0 | 1.3 | 0 | [0 - 1.8] | [0 - 4.3] | 0.8 | 1.0 | 0 | [0 - 1.3] | [0 - 3.0] | 0.20 [ -0.60, 1.00] |
| POW 2 | 0.5 | 1.0 | 0 | [0 - 1.0] | [0 - 4.0] | 0.8 | 1.4 | 0 | [0 - 0.8] | [0 - 4.0] | -0.20 [ -1.10, 0.60] |
| POW 3 | 0.2 | 0.5 | 0 | [0 - 0] | [0 - 1.8] | 0.2 | 0.7 | 0 | [0 - 0] | [0 - 2.8] | 0.00 [ -0.40, 0.40] |
| POW 4 | 0.1 | 0.3 | 0 | [0 - 0] | [0 - 1.0] | 0.1 | 0.3 | 0 | [0 - 0] | [0 - 1.3] | 0.10 [ -0.10, 0.30] |

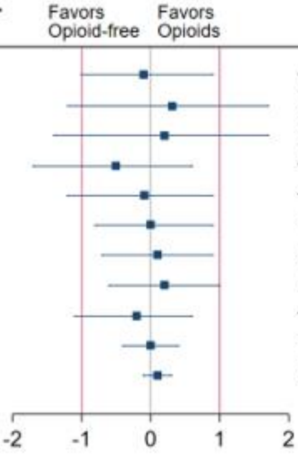


**B. Pain interference**

| | Opioid-free analgesia | | | | | Opioid analgesia | | | | | |
|---|---|---|---|---|---|---|---|---|---|---|---|
| Timepoint | Mean | SD | Median | IQR | Range | Mean | SD | Median | IQR | Range | Mean difference [95% CI] |
| Baseline | 0.3 | 0.9 | 0 | [0 - 0] | [0 - 3.6] | 0.5 | 1.5 | 0 | [0 - 0] | [0 - 6.1] | -0.20 [ -1.10, 0.60] |
| POD 1 | 2.3 | 2.0 | 2.0 | [0.6 - 3.4] | [0 - 7.3] | 2.1 | 3.0 | 0.7 | [0 - 3.1] | [0 - 8.9] | 0.10 [ -1.60, 1.90] |
| POD 2 | 1.5 | 1.5 | 1.4 | [0 - 2.6] | [0 - 4.4] | 1.6 | 2.5 | 0.4 | [0 - 1.7] | [0 - 7.4] | -0.10 [ -1.50, 1.30] |
| POD 3 | 0.9 | 1.1 | 0.3 | [0 - 1.9] | [0 - 3.0] | 1.5 | 2.5 | 0.1 | [0 - 2.3] | [0 - 8.9] | -0.60 [ -2.00, 0.70] |
| POD 4 | 0.9 | 1.2 | 0.3 | [0 - 1.6] | [0 - 3.7] | 1.7 | 2.8 | 0.1 | [0 - 2.1] | [0 - 10.0] | -0.80 [ -2.30, 0.70] |
| POD 5 | 0.5 | 0.8 | 0 | [0 - 0.7] | [0 - 2.4] | 0.8 | 1.7 | 0 | [0 - 0.6] | [0 - 6.6] | -0.20 [ -1.10, 0.70] |
| POD 6 | 0.4 | 0.8 | 0 | [0 - 0.3] | [0 - 3.1] | 0.7 | 1.8 | 0 | [0 - 0.4] | [0 - 7.4] | -0.40 [ -1.30, 0.60] |
| POD 7 | 0.4 | 0.8 | 0 | [0 - 0.4] | [0 - 3.3] | 0.7 | 1.8 | 0 | [0 - 0.4] | [0 - 7.4] | -0.30 [ -1.30, 0.70] |
| POW 2 | 0.5 | 1.8 | 0 | [0 - 0] | [0 - 7.6] | 0.9 | 1.8 | 0 | [0 - 1.4] | [0 - 5.9] | -0.40 [ -1.60, 0.80] |
| POW 3 | 0 | 0.2 | 0 | [0 - 0] | [0 - 0.7] | 0.2 | 0.5 | 0 | [0 - 0] | [0 - 2.3] | -0.10 [ -0.40, 0.20] |
| POW 4 | 0 | 0 | 0 | [0 - 0] | [0 - 0.1] | 0 | 0 | 0 | [0 - 0] | [0 - 0] | 0.00 [ 0.00, 0.00] |

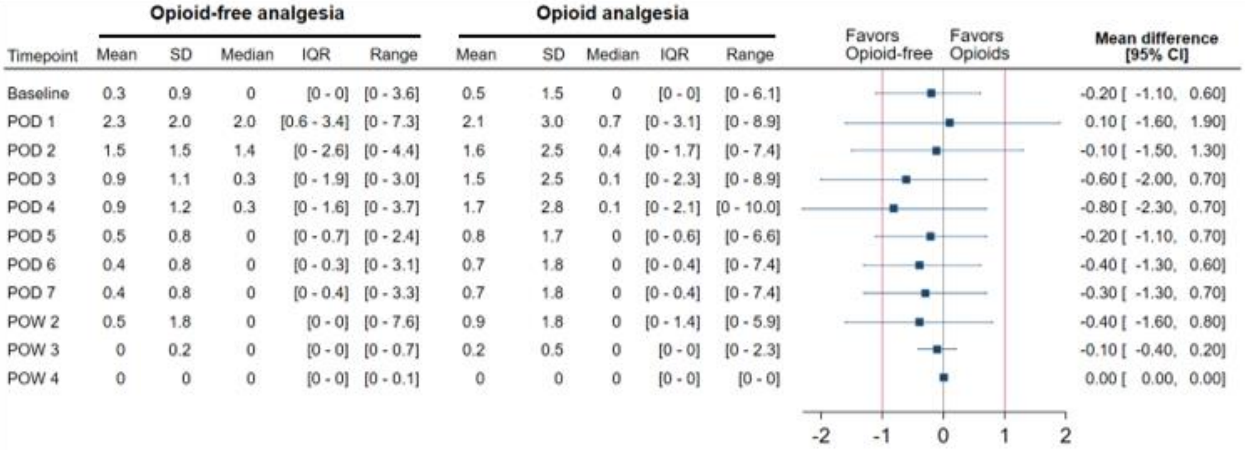


**eFigure 5. Subgroup analysis of Brief Pain Inventory in patients undergoing breast surgery. Plots represent between-group differences in the Brief Pain Inventory severity scale (composite of 4 items, score 0-10) and interference scale (composite of 7 items, score 0-10). Red lines represent minimal clinically important differences[18,19]. POW3 OFA n =35; POW4 OFA n = 34 (due to losses of follow-up).**

© 2022 Do U et al. *JAMA Network Open*.

**A. Physical functioning**

| | Opioid-free analgesia | | | | | Opioid analgesia | | | | | |
|---|---|---|---|---|---|---|---|---|---|---|---|
| Timepoint | Mean | SD | Median | IQR | Range | Mean | SD | Median | IQR | Range | Mean difference [95% CI] |
| Baseline | 50.3 | 8.5 | 57.0 | [44.5 - 57.0] | [30.6 - 57.0] | 50.0 | 8.6 | 57.0 | [41.5 - 57.0] | [33.5 - 57.0] | 0.40 [ -5.10, 5.90] |
| POW 1 | 41.5 | 8.6 | 42.1 | [36.1 - 47.8] | [22.6 - 57.0] | 44.8 | 7.7 | 42.9 | [39.1 - 57.0] | [31.6 - 57.0] | -3.30 [ -8.50, 1.90] |
| POW 2 | 47.9 | 6.9 | 47.8 | [42.8 - 57.0] | [34.8 - 57.0] | 47.3 | 8.2 | 45.2 | [41.5 - 57.0] | [32.5 - 57.0] | 0.60 [ -4.30, 5.40] |
| POW 3 | 50.8 | 6.8 | 52.4 | [45.1 - 57.0] | [35.5 - 57.0] | 50.0 | 8.6 | 57.0 | [42.3 - 57.0] | [34.8 - 57.0] | 0.90 [ -4.10, 5.80] |
| POW 4 | 51.5 | 7.0 | 57.0 | [45.1 - 57.0] | [39.1 - 57.0] | 51.8 | 7.4 | 57.0 | [43.1 - 57.0] | [38.8 - 57.0] | -0.20 [ -4.90, 4.40] |

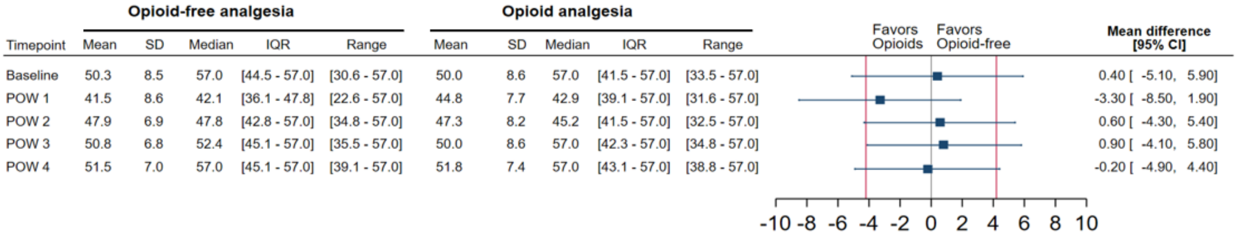


**B. Social participation**

| | Opioid-free analgesia | | | | | Opioid analgesia | | | | | |
|---|---|---|---|---|---|---|---|---|---|---|---|
| Timepoint | Mean | SD | Median | IQR | Range | Mean | SD | Median | IQR | Range | Mean difference [95% CI] |
| Baseline | 52.0 | 7.5 | 52.0 | [47.1 - 55.7] | [35.1 - 64.2] | 55.1 | 9.4 | 57.2 | [49.1 - 64.2] | [38.5 - 64.2] | -3.10 [ -8.50, 2.30] |
| POW 1 | 45.2 | 10.5 | 46.8 | [38.7 - 51.8] | [27.5 - 64.2] | 49.2 | 8.4 | 50.8 | [43.0 - 54.6] | [34.1 - 64.2] | -4.00 [ -10.10, 2.00] |
| POW 2 | 53.0 | 8.5 | 51.8 | [45.2 - 61.4] | [40.3 - 64.2] | 55.3 | 8.7 | 57.0 | [49.7 - 64.2] | [38.8 - 64.2] | -2.30 [ -7.80, 3.20] |
| POW 3 | 54.6 | 9.1 | 54.7 | [49.3 - 64.2] | [37.4 - 64.2] | 57.4 | 8.4 | 61.4 | [51.0 - 64.2] | [37.2 - 64.2] | -2.90 [ -8.50, 2.70] |
| POW 4 | 54.6 | 8.9 | 51.9 | [44.2 - 64.2] | [37.8 - 64.2] | 56.7 | 9.8 | 64.2 | [50.9 - 64.2] | [35.1 - 64.2] | -2.10 [ -8.20, 4.00] |

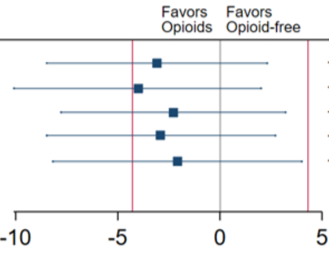


**C. Anxiety**

| | Opioid-free analgesia | | | | | Opioid analgesia | | | | | |
|---|---|---|---|---|---|---|---|---|---|---|---|
| Timepoint | Mean | SD | Median | IQR | Range | Mean | SD | Median | IQR | Range | Mean difference [95% CI] |
| Baseline | 50.5 | 8.3 | 52.0 | [40.3 - 56.0] | [40.3 - 61.8] | 51.4 | 9.3 | 54.0 | [40.3 - 56.0] | [40.3 - 67.4] | -0.90 [ -6.50, 4.80] |
| POW 1 | 48.8 | 7.8 | 49.7 | [40.3 - 52.9] | [40.3 - 68.1] | 47.3 | 8.4 | 40.3 | [40.3 - 55.7] | [40.3 - 65.1] | 1.50 [ -3.70, 6.70] |
| POW 2 | 44.1 | 6.2 | 40.3 | [40.3 - 49.3] | [40.3 - 56.0] | 45.9 | 8.1 | 40.3 | [40.3 - 51.4] | [40.3 - 65.2] | -1.80 [ -6.40, 2.80] |
| POW 3 | 41.4 | 3.4 | 40.3 | [40.3 - 40.3] | [40.3 - 54.1] | 45.4 | 6.9 | 40.3 | [40.3 - 51.4] | [40.3 - 59.7] | -4.00 [ -7.50, -0.50] |
| POW 4 | 43.7 | 6.7 | 40.3 | [40.3 - 40.3] | [40.3 - 57.9] | 45.5 | 7.5 | 40.3 | [40.3 - 48.4] | [40.3 - 63.5] | -1.90 [ -6.50, 2.80] |

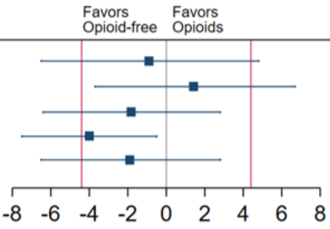


**D. Depression**

| | Opioid-free analgesia | | | | | Opioid analgesia | | | | | |
|---|---|---|---|---|---|---|---|---|---|---|---|
| Timepoint | Mean | SD | Median | IQR | Range | Mean | SD | Median | IQR | Range | Mean difference [95% CI] |
| Baseline | 48.3 | 7.8 | 46.1 | [41.0 - 56.0] | [41.0 - 61.2] | 45.6 | 7.4 | 41.0 | [41.0 - 49.2] | [41.0 - 64.0] | 2.60 [ -2.20, 7.50] |
| POW 1 | 46.2 | 7.1 | 41.0 | [41.0 - 51.0] | [41.0 - 60.6] | 44.9 | 6.2 | 41.0 | [41.0 - 48.9] | [41.0 - 60.6] | 1.30 [ -3.00, 5.60] |
| POW 2 | 43.8 | 6.0 | 41.0 | [41.0 - 41.0] | [41.0 - 58.7] | 43.0 | 6.3 | 41.0 | [41.0 - 41.0] | [41.0 - 62.2] | 0.80 [ -3.20, 4.70] |
| POW 3 | 43.5 | 4.7 | 41.0 | [41.0 - 45.0] | [41.0 - 57.7] | 45.5 | 7.5 | 41.0 | [41.0 - 50.6] | [41.0 - 60.6] | -2.00 [ -6.00, 2.00] |
| POW 4 | 44.4 | 6.1 | 41.0 | [41.0 - 48.9] | [41.0 - 57.5] | 44.8 | 6.2 | 41.0 | [41.0 - 49.2] | [41.0 - 57.5] | -0.40 [ -4.40, 3.60] |

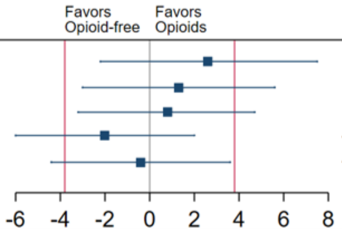


**E. Pain interference**

| | Opioid-free analgesia | | | | | Opioid analgesia | | | | | |
|---|---|---|---|---|---|---|---|---|---|---|---|
| Timepoint | Mean | SD | Median | IQR | Range | Mean | SD | Median | IQR | Range | Mean difference [95% CI] |
| Baseline | 50.1 | 9.4 | 47.1 | [41.6 - 57.4] | [41.6 - 68.9] | 50.7 | 9.7 | 47.8 | [41.6 - 60.1] | [41.6 - 65.5] | -0.60 [ -6.70, 5.50] |
| POW 1 | 57.4 | 9.4 | 57.9 | [53.3 - 61.9] | [41.6 - 75.6] | 57.3 | 6.9 | 55.7 | [53.8 - 61.3] | [41.6 - 71.3] | 0.10 [ -5.20, 5.40] |
| POW 2 | 49.0 | 7.9 | 46.6 | [41.6 - 55.7] | [41.6 - 61.3] | 51.3 | 9.1 | 52.8 | [41.6 - 57.5] | [41.6 - 67.8] | -2.30 [ -7.70, 3.20] |
| POW 3 | 46.5 | 7.1 | 41.6 | [41.6 - 53.9] | [41.6 - 61.3] | 49.2 | 8.4 | 45.9 | [41.6 - 55.8] | [41.6 - 65.2] | -2.70 [ -7.70, 2.20] |
| POW 4 | 48.1 | 7.8 | 41.6 | [41.6 - 53.7] | [41.6 - 62.3] | 47.0 | 7.7 | 41.6 | [41.6 - 54.8] | [41.6 - 61.1] | 1.10 [ -3.90, 6.10] |

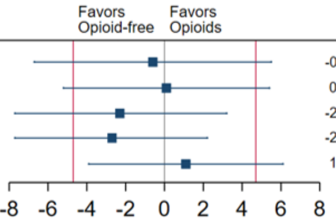


**F. Sleep disturbance**

| | Opioid-free analgesia | | | | | Opioid analgesia | | | | | |
|---|---|---|---|---|---|---|---|---|---|---|---|
| Timepoint | Mean | SD | Median | IQR | Range | Mean | SD | Median | IQR | Range | Mean difference [95% CI] |
| Baseline | 46.9 | 10.3 | 50.3 | [36.6 - 54.4] | [32.0 - 61.4] | 47.6 | 10.0 | 48.0 | [41.2 - 51.4] | [32.0 - 66.4] | -0.70 [ -7.20, 5.80] |
| POW 1 | 47.3 | 9.9 | 45.8 | [39.8 - 52.9] | [32.0 - 69.4] | 49.1 | 8.8 | 47.3 | [44.0 - 56.7] | [32.0 - 63.8] | -1.90 [ -7.90, 4.10] |
| POW 2 | 44.5 | 9.9 | 46.1 | [33.1 - 52.8] | [32.0 - 63.8] | 45.8 | 7.9 | 47.5 | [41.2 - 50.4] | [32.0 - 58.7] | -1.30 [ -7.00, 4.40] |
| POW 3 | 44.9 | 10.3 | 45.1 | [32.0 - 53.6] | [32.0 - 60.6] | 44.5 | 9.2 | 42.4 | [38.4 - 51.2] | [32.0 - 62.0] | 0.40 [ -5.90, 6.60] |
| POW 4 | 43.6 | 10.5 | 44.2 | [32.0 - 51.1] | [32.0 - 63.4] | 43.4 | 8.2 | 43.5 | [36.9 - 50.4] | [32.0 - 61.9] | 0.20 [ -5.80, 6.30] |

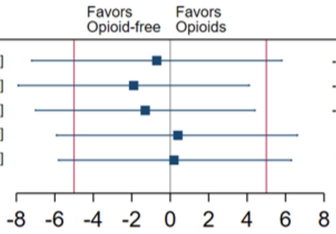


**G. Fatigue**

| | Opioid-free analgesia | | | | | Opioid analgesia | | | | | |
|---|---|---|---|---|---|---|---|---|---|---|---|
| Timepoint | Mean | SD | Median | IQR | Range | Mean | SD | Median | IQR | Range | Mean difference [95% CI] |
| Baseline | 48.0 | 9.7 | 48.6 | [38.5 - 57.1] | [33.7 - 61.3] | 45.0 | 8.6 | 46.0 | [36.7 - 51.0] | [33.7 - 60.7] | 3.00 [ -2.90, 8.90] |
| POW 1 | 50.9 | 11.1 | 47.7 | [43.2 - 59.3] | [33.7 - 75.8] | 49.4 | 10.1 | 48.6 | [44.1 - 56.5] | [33.7 - 71.5] | 1.50 [ -5.30, 8.30] |
| POW 2 | 44.7 | 10.0 | 46.0 | [33.7 - 49.8] | [33.7 - 64.7] | 42.3 | 10.4 | 36.8 | [33.7 - 52.1] | [33.7 - 62.7] | 2.40 [ -4.10, 8.90] |
| POW 3 | 41.8 | 9.9 | 38.4 | [33.7 - 46.1] | [33.7 - 64.7] | 42.2 | 8.2 | 41.9 | [33.7 - 49.8] | [33.7 - 55.2] | -0.40 [ -6.20, 5.40] |
| POW 4 | 43.3 | 10.8 | 39.8 | [33.7 - 53.1] | [33.7 - 64.8] | 40.9 | 7.5 | 40.2 | [33.7 - 48.3] | [33.7 - 53.2] | 2.40 [ -3.60, 8.40] |

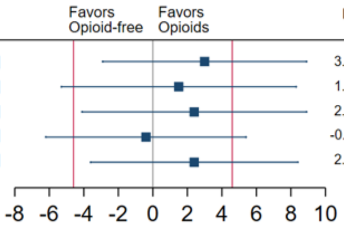


**eFigure 6. Subgroup analysis of PROMIS-29 domains T-scores in patients undergoing abdominal surgery. Red lines represent minimal clinically important differences (MCID) (MCIDs were estimated as 0.5 standard deviation at baseline for each domain[17]). POW3 OFA n =35; POW4 OFA n = 34 (due to losses of follow-up).**

Higher scores on physical function and social participation domains indicate desirable outcomes. Higher scores on anxiety, depression, pain interference, sleep disturbance, and fatigue domains indicate undesirable outcomes. Pain intensity score is not based on the same unit of measure of the other domains (T-scores) and, therefore, is not included in the graph. Pain intensity scores are not based on the same unit of measure of the other domains (T-scores) and, therefore, they are reported separately in the table below.

© 2022 Do U et al. *JAMA Network Open*.

## PROMIS-29® pain intensity scores in patients undergoing abdominal surgery[a]

| | Opioid analgesia (n=20) | | | Opioid-free analgesia (n=20) | | | Between-group difference (95%CI)[b] |
|---|---|---|---|---|---|---|---|
| | Mean (SD) | Median (IQR) | Range (max – min) | Mean (SD) | Median (IQR) | Range (max – min) | |
| Baseline | 2.5 (2.3) | 1.5 (0.5-4.5) | 7 (0-7) | 2.7 (2.6) | 2 (0.5-4) | 8 (0-8) | 0.2 (-1.3 to 1.8) |
| Postoperative week 1 | 2.8 (1.7) | 3 (1-4) | 6 (0-6) | 3.2 (1.9) | 3 (1.5-4) | 7 (0-7) | 0.4 (-1.5 to 0.8) |
| Postoperative week 2 | 1.8 (1.8) | 1 (0-3) | 6 (0-6) | 0.9 (0.9) | 1 (0-1.5) | 3 (0-3) | -0.9 (-0.1 to 1.8) |
| Postoperative week 3 | 1.1 (1.5) | 0.5 (0-2) | 5 (0-5) | 0.8 (1.2) | 0 (0-1) | 4 (0-4) | -0.3 (-0.6 to 1.1) |
| Postoperative week 4 | 1.2 (1.8) | 0.5 (0-1.5) | 7 (0-7) | 0.9 (1.2) | 1 (0-1) | 5 (0-5) | -0.3 (-0.8 to 1.3) |

Data are mean (SD). PROMIS® = Patient-Reported Outcomes Measurement Information System. POW3 OFA n =19; POW4 OFA n = 19 (due to losses of follow-up).

[a] Pain intensity rating is a 11-point rating scale (range, 0-10; 0 = no pain and 10 = worst imaginable pain). Recall period is 7 days.

[b] Between-group difference represents mean difference.

© 2022 Do U et al. *JAMA Network Open*.

**A. Physical functioning**

| Timepoint | Opioid-free analgesia Mean | SD | Median | IQR | Range | Opioid analgesia Mean | SD | Median | IQR | Range | Mean difference [95% CI] |
|---|---|---|---|---|---|---|---|---|---|---|---|
| Baseline | 51.3 | 8.9 | 57.0 | [47.8 - 57.0] | [33.9 - 57.0] | 50.4 | 8.3 | 57.0 | [45.1 - 57.0] | [31.1 - 57.0] | 0.80 [ -5.00, 6.60] |
| POW 1 | 47.1 | 8.9 | 46.1 | [41.6 - 57.0] | [32.2 - 57.0] | 45.0 | 9.1 | 44.6 | [40.9 - 57.0] | [27.9 - 57.0] | 2.10 [ -4.00, 8.20] |
| POW 2 | 47.9 | 9.5 | 47.8 | [43.8 - 57.0] | [27.7 - 57.0] | 47.2 | 6.9 | 45.7 | [41.6 - 57.0] | [33.6 - 57.0] | 0.70 [ -4.90, 6.30] |
| POW 3 | 50.3 | 8.0 | 56.3 | [43.7 - 57.0] | [36.6 - 57.0] | 49.3 | 7.0 | 47.8 | [44.6 - 57.0] | [34.5 - 57.0] | 1.10 [ -4.20, 6.30] |
| POW 4 | 51.4 | 7.7 | 57.0 | [46.1 - 57.0] | [37.2 - 57.0] | 52.4 | 6.6 | 57.0 | [47.8 - 57.0] | [36.9 - 57.0] | -1.10 [ -6.00, 3.90] |

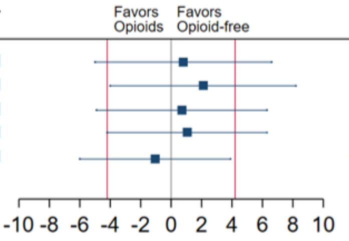


**B. Social participation**

| Timepoint | Opioid-free analgesia Mean | SD | Median | IQR | Range | Opioid analgesia Mean | SD | Median | IQR | Range | Mean difference [95% CI] |
|---|---|---|---|---|---|---|---|---|---|---|---|
| Baseline | 54.0 | 10.1 | 55.7 | [46.1 - 64.2] | [32.9 - 64.2] | 54.8 | 9.7 | 57.5 | [44.2 - 64.2] | [38.5 - 64.2] | -0.90 [ -7.60, 5.80] |
| POW 1 | 49.7 | 10.0 | 46.0 | [44.2 - 57.6] | [33.9 - 64.2] | 49.9 | 11.0 | 50.2 | [40.5 - 64.2] | [31.6 - 64.2] | -0.20 [ -7.40, 7.00] |
| POW 2 | 56.0 | 8.0 | 55.8 | [49.6 - 64.2] | [37.2 - 64.2] | 53.7 | 9.0 | 55.9 | [44.5 - 64.2] | [38.6 - 64.2] | 2.30 [ -3.40, 8.10] |
| POW 3 | 56.9 | 7.9 | 58.5 | [51.8 - 64.2] | [40.3 - 64.2] | 55.5 | 7.7 | 56.9 | [48.3 - 64.2] | [42.4 - 64.2] | 1.50 [ -4.00, 7.00] |
| POW 4 | 59.3 | 7.2 | 64.2 | [57.6 - 64.2] | [42.4 - 64.2] | 57.8 | 6.8 | 58.5 | [51.8 - 64.2] | [46.1 - 64.2] | 1.50 [ -3.40, 6.40] |

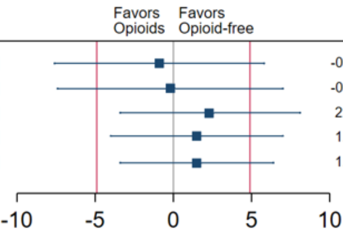


**C. Anxiety**

| Timepoint | Opioid-free analgesia Mean | SD | Median | IQR | Range | Opioid analgesia Mean | SD | Median | IQR | Range | Mean difference [95% CI] |
|---|---|---|---|---|---|---|---|---|---|---|---|
| Baseline | 56.1 | 8.0 | 56.0 | [48.1 - 63.5] | [40.3 - 61.8] | 55.4 | 10.3 | 56.0 | [40.3 - 65.2] | [40.3 - 71.4] | 0.70 [ -5.60, 7.00] |
| POW 1 | 48.4 | 8.6 | 48.1 | [40.3 - 52.9] | [40.3 - 56.0] | 45.6 | 8.5 | 40.3 | [40.3 - 53.8] | [40.3 - 63.5] | 2.80 [ -3.00, 8.60] |
| POW 2 | 45.5 | 7.8 | 40.3 | [40.3 - 49.3] | [40.3 - 51.7] | 45.9 | 8.4 | 40.3 | [40.3 - 51.4] | [40.3 - 63.5] | -0.40 [ -6.00, 5.10] |
| POW 3 | 45.6 | 7.1 | 40.3 | [40.3 - 40.3] | [40.3 - 54.1] | 43.4 | 5.9 | 40.3 | [40.3 - 48.1] | [40.3 - 59.4] | 2.30 [ -2.30, 6.80] |
| POW 4 | 45.4 | 6.8 | 40.3 | [40.3 - 40.3] | [40.3 - 54.7] | 43.4 | 5.6 | 40.3 | [40.3 - 47.9] | [40.3 - 57.5] | 2.00 [ -2.40, 6.30] |

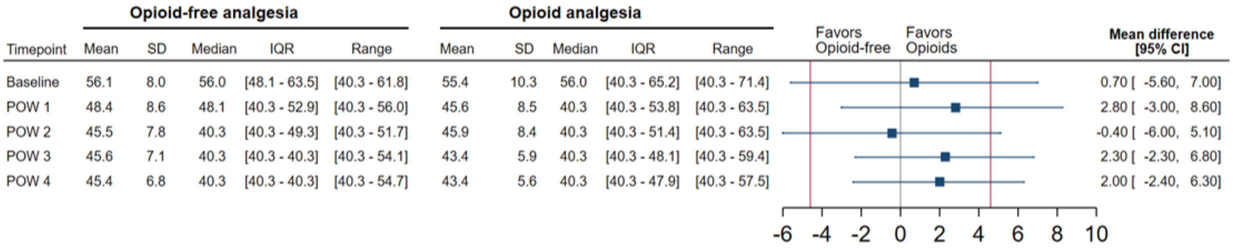


**D. Depression**

| Timepoint | Opioid-free analgesia Mean | SD | Median | IQR | Range | Opioid analgesia Mean | SD | Median | IQR | Range | Mean difference [95% CI] |
|---|---|---|---|---|---|---|---|---|---|---|---|
| Baseline | 47.6 | 8.9 | 41.0 | [41.0 - 55.2] | [41.0 - 69.5] | 48.6 | 9.8 | 41.0 | [41.0 - 58.3] | [41.0 - 67.5] | -1.10 [ -7.40, 5.30] |
| POW 1 | 46.3 | 7.4 | 41.0 | [41.0 - 49.3] | [41.0 - 62.2] | 46.4 | 7.6 | 41.0 | [41.0 - 55.4] | [41.0 - 60.5] | -0.10 [ -5.20, 5.00] |
| POW 2 | 43.4 | 5.8 | 41.0 | [41.0 - 41.0] | [41.0 - 62.2] | 47.2 | 6.5 | 49.1 | [41.0 - 53.0] | [41.0 - 59.2] | -3.80 [ -8.00, 0.40] |
| POW 3 | 44.0 | 5.5 | 41.0 | [41.0 - 48.9] | [41.0 - 55.9] | 43.9 | 5.1 | 41.0 | [41.0 - 48.9] | [41.0 - 54.1] | 0.10 [ -3.50, 3.80] |
| POW 4 | 44.3 | 6.0 | 41.0 | [41.0 - 48.9] | [41.0 - 58.7] | 43.4 | 4.2 | 41.0 | [41.0 - 48.9] | [41.0 - 52.2] | 0.90 [ -2.70, 4.50] |

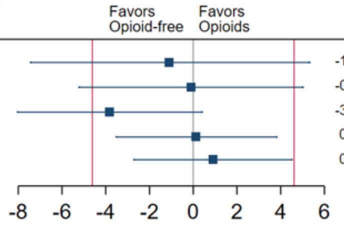


**E. Pain interference**

| Timepoint | Opioid-free analgesia Mean | SD | Median | IQR | Range | Opioid analgesia Mean | SD | Median | IQR | Range | Mean difference [95% CI] |
|---|---|---|---|---|---|---|---|---|---|---|---|
| Baseline | 46.5 | 8.2 | 41.6 | [41.6 - 53.9] | [41.6 - 65.4] | 47.3 | 8.1 | 41.6 | [41.6 - 55.7] | [41.6 - 65.2] | -0.90 [ -6.40, 4.70] |
| POW 1 | 53.2 | 8.5 | 55.7 | [41.6 - 59.0] | [41.6 - 65.0] | 54.1 | 9.4 | 53.9 | [41.6 - 61.3] | [41.6 - 69.4] | -0.90 [ -7.00, 5.20] |
| POW 2 | 51.0 | 6.1 | 52.6 | [49.2 - 53.9] | [41.6 - 61.3] | 49.6 | 8.9 | 50.2 | [41.6 - 55.7] | [41.6 - 66.7] | 1.30 [ -3.90, 6.60] |
| POW 3 | 46.5 | 7.8 | 41.6 | [41.6 - 53.7] | [41.6 - 64.7] | 47.7 | 6.9 | 41.6 | [41.6 - 53.9] | [41.6 - 60.3] | -1.20 [ -6.30, 4.00] |
| POW 4 | 44.9 | 5.8 | 41.6 | [41.6 - 51.7] | [41.6 - 56.9] | 44.7 | 5.7 | 41.6 | [41.6 - 49.2] | [41.6 - 60.0] | 0.30 [ -3.80, 4.30] |

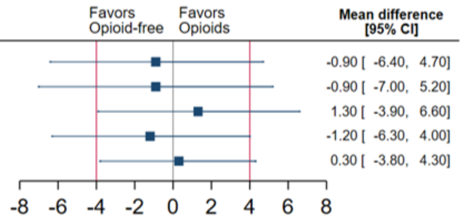


**F. Sleep disturbance**

| Timepoint | Opioid-free analgesia Mean | SD | Median | IQR | Range | Opioid analgesia Mean | SD | Median | IQR | Range | Mean difference [95% CI] |
|---|---|---|---|---|---|---|---|---|---|---|---|
| Baseline | 52.0 | 5.1 | 52.8 | [49.4 - 55.5] | [41.2 - 62.1] | 45.9 | 9.7 | 44.7 | [41.2 - 54.5] | [32.0 - 69.4] | 5.10 [ 0.80, 11.50] |
| POW 1 | 47.5 | 8.4 | 47.5 | [44.2 - 52.8] | [32.0 - 61.5] | 44.9 | 7.8 | 46.4 | [41.2 - 51.1] | [32.0 - 57.4] | 2.70 [ -2.80, 8.20] |
| POW 2 | 49.8 | 7.0 | 51.1 | [46.4 - 54.4] | [32.0 - 54.4] | 44.6 | 7.2 | 45.1 | [41.2 - 49.6] | [32.0 - 55.8] | 5.20 [ 0.40, 10.00] |
| POW 3 | 47.7 | 8.0 | 49.3 | [42.1 - 53.3] | [32.0 - 59.9] | 45.5 | 7.6 | 43.8 | [41.2 - 51.1] | [32.0 - 60.5] | 2.20 [ -3.20, 7.60] |
| POW 4 | 45.2 | 7.9 | 46.3 | [41.2 - 49.6] | [32.0 - 59.8] | 43.9 | 9.0 | 45.5 | [32.0 - 51.1] | [32.0 - 61.9] | 1.30 [ -4.70, 7.20] |

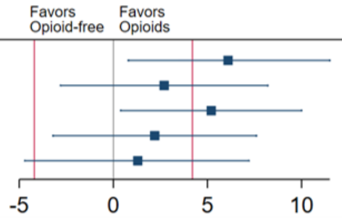


**G. Fatigue**

| Timepoint | Opioid-free analgesia Mean | SD | Median | IQR | Range | Opioid analgesia Mean | SD | Median | IQR | Range | Mean difference [95% CI] |
|---|---|---|---|---|---|---|---|---|---|---|---|
| Baseline | 47.3 | 10.5 | 43.4 | [39.7 - 57.1] | [33.7 - 64.7] | 46.9 | 10.5 | 48.6 | [33.7 - 55.2] | [33.7 - 66.6] | 0.40 [ -6.70, 7.50] |
| POW 1 | 49.4 | 11.6 | 48.6 | [43.1 - 55.5] | [33.7 - 75.8] | 47.3 | 8.7 | 46.0 | [43.1 - 53.2] | [33.7 - 64.7] | 2.10 [ -4.80, 9.00] |
| POW 2 | 44.5 | 10.9 | 46.0 | [33.7 - 48.6] | [33.7 - 64.7] | 43.7 | 8.8 | 43.3 | [33.7 - 48.6] | [33.7 - 59.0] | 0.80 [ -5.90, 7.50] |
| POW 3 | 43.2 | 10.2 | 39.8 | [33.7 - 48.6] | [33.7 - 64.7] | 41.4 | 8.4 | 39.8 | [33.7 - 48.6] | [33.7 - 57.1] | 1.80 [ -4.70, 8.30] |
| POW 4 | 40.7 | 8.6 | 33.7 | [33.7 - 48.6] | [33.7 - 58.8] | 41.0 | 8.1 | 33.7 | [33.7 - 48.6] | [33.7 - 55.2] | -0.20 [ -6.10, 5.60] |

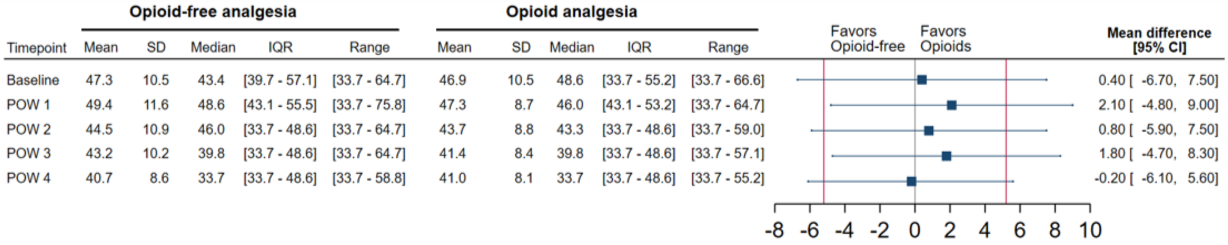


**eFigure 7. Subgroup analysis of PROMIS-29 domains T-scores in patients undergoing breast surgery. Red lines represent minimal clinically important differences (MCID) (MCIDs were estimated as 0.5 standard deviation at baseline for each domain[17]). POW3 OFA n =35; POW4 OFA n = 34 (due to losses of follow-up).**

Higher scores on physical function and social participation domains indicate desirable outcomes. Higher scores on anxiety, depression, pain interference, sleep disturbance, and fatigue domains indicate undesirable outcomes. Pain intensity scores are not based on the same unit of measure of the other domains (T-scores) and, therefore, they are reported separately in the table below.

© 2022 Do U et al. *JAMA Network Open*.

## PROMIS-29® pain intensity scores in patients undergoing breast surgery[a]

| | Opioid analgesia (n=19) | | | Opioid-free analgesia (n=17) | | | Between-group difference (95%CI)[b] |
|---|---|---|---|---|---|---|---|
| | Mean (SD) | Median (IQR) | Range (max – min) | Mean (SD) | Median (IQR) | Range (max – min) | |
| Baseline | 1.8 (2.6) | 1 (0-3) | 9 (0-9) | 1.6 (2.5) | 0 (0-3) | 7 (0-7) | -0.2 (-1.5 to 1.9) |
| Postoperative week 1 | 2.2 (2.1) | 2 (1-4) | 7 (0-7) | 2.1 (2.2) | 2 (0-3) | 6 (0-6) | -0.1 (-1.4 to 1.6) |
| Postoperative week 2 | 1.3 (1.6) | 1 (0-2) | 6 (0-6) | 1.6 (1.5) | 1 (0-2) | 6 (0-6) | 0.4 (-1.4 to 0.7) |
| Postoperative week 3 | 0.6 (0.8) | 0 (0-1) | 2 (0-2) | 1.2 (1.6) | 1 (0-2) | 6 (0-6) | 0.6 (-1.5 to 0.2) |
| Postoperative week 4 | 0.3 (0.6) | 0 (0-1) | 2 (0-2) | 0.6 (1.0) | 0 (0-1) | 3 (0-3) | 0.3 (-0.8 to 0.3) |

Data are mean (SD). PROMIS® = Patient-Reported Outcomes Measurement Information System. POW3 OFA n =15; POW4 OFA n = 15 (due to losses of follow-up).

[a] Pain intensity rating is a 11-point rating scale (range, 0-10; 0 = no pain and 10 = worst imaginable pain). Recall period is 7 days.

[b] Between-group difference represents mean difference.

© 2022 Do U et al. *JAMA Network Open*.

## eTable 5: Postoperative pain management regimens

| | Total | Abdominal surgery | Breast surgery |
|---|---|---|---|
| **Opioid analgesia group, n** | 39 | 20 | 19 |
| Acetaminophen (ATC) + Oxycodone (PRN) | 19 (49) | 1 (5) | 18 (95) |
| Acetaminophen (ATC) + Naproxen (ATC) + Oxycodone (PRN) | 16 (41) | 16 (80) | 0 (0) |
| Acetaminophen (ATC) + Ibuprofen (ATC) + Oxycodone (PRN) | 1 (3) | 1 (5) | 0 (0) |
| Acetaminophen (ATC) + Celecoxib (ATC) + Oxycodone (PRN) | 1 (3) | 1 (5) | 0 (0) |
| Acetaminophen (ATC) + Naproxen (ATC) + Hydromorphone (PRN) | 1 (3) | 1 (5) | 0 (0) |
| Acetaminophen (ATC) + Hydromorphone (PRN) | 1 (3) | 0 (0) | 1 (5) |
| *Amount of opioid prescribed at discharge (MME)* | *106 (82)* | *133 (88)* | *78 (67)* |
| **Opioid-free analgesia group, n** | 37 | 20 | 17 |
| Acetaminophen (ATC) + Celecoxib (PRN) | 17 (46) | 0 (0) | 17 (100) |
| Acetaminophen (ATC) + Naproxen (ATC, switch to Ibuprofen if breakthrough pain) | 17 (46) | 17 (85) | 0 (0) |
| Acetaminophen (ATC) + Celecoxib [ATC, switch to Ibuprofen or take one additional Celecoxib tablet if breakthrough pain] | 3 (8) | 3 (15) | 0 (0) |

Data are n (%) or mean (SD). MME = Morphine Milligram Equivalent; ATC = around-the-clock; PRN = *pro re nata* (as needed).

© 2022 Do U et al. *JAMA Network Open*.

**eTable 6. Adverse events identified via the Perioperative Opioid-Related Symptom Distress Scale**

| Adverse events | Opioid analgesia (n=39) | Opioid-free analgesia (n=37) | Between-group difference (95%CI)[a] |
|---|---|---|---|
| **Constipation** | | | |
| 7-day rate (any event) | 16 (41) | 12 (32) | -9 (-30.2 to 13.0) |
| 7-day rate (clinically meaningful event) | 4 (10) | 2 (5) | -5 (-16.8 to 7.1) |
| 30-day rate (any event) | 18 (46) | 15 (41) | -5 (-27.9 to 16.6) |
| 30-day rate (clinically meaningful event) | 7 (18) | 3 (8) | -10 (-33.9 to 14.2) |
| **Nausea** | | | |
| 7-day rate (any event) | 8 (21) | 6 (16) | -5 (-21.7 to 13.1) |
| 7-day rate (clinically meaningful event) | 1 (3) | 1 (3) | 0 (-7.1 to 7.3) |
| 30-day rate (any event) | 9 (23) | 8 (22) | -1 (-20.2 to 17.3) |
| 30-day rate (clinically meaningful event) | 1 (3) | 1 (3) | 0 (-7.1 to 7.3) |
| **Vomiting** | | | |
| 7-day rate (any event) | 3 (8) | 1 (3) | -5 (-14.9 to 4.9) |
| 7-day rate (clinically meaningful event) | 0 (0) | 1 (3) | 3 (-2.5 to 7.9) |
| 30-day rate (any event) | 6 (16) | 1 (3) | -13 (-25.2 to -0.2) |
| 30-day rate (clinically meaningful event) | 0 (0) | 1 (3) | 3 (-2.5 to 7.9) |
| **Itching** | | | |
| 7-day rate (any event) | 13 (33) | 7 (19) | -14 (-33.9 to 5.0) |
| 7-day rate (clinically meaningful event) | 1 (3) | 1 (3) | 0 (-7.1 to 7.3) |
| 30-day rate (any event) | 15 (38) | 15 (40) | 2 (-19.9 to 24.1) |
| 30-day rate (clinically meaningful event) | 1 (3) | 2 (5) | 2 (-6.2 to 11.8) |
| **Fatigue** | | | |
| 7-day rate (any event) | 31 (79) | 28 (76) | -3 (-22.6 to 14.9) |
| 7-day rate (clinically meaningful event) | 2 (5) | 5 (13) | 8 (-4.6 to 21.4) |
| 30-day rate (any event) | 32 (82) | 30 (81) | -1 (-18.4 to 16.5) |
| 30-day rate (clinically meaningful event) | 3 (8) | 11 (30) | 22 (-3.2 to 47.3) |
| **Drowsiness** | | | |
| 7-day rate (any event) | 14 (36) | 13 (35) | -1 (-22.3 to 20.8) |
| 7-day rate (clinically meaningful event) | 0 (0) | 1 (3) | 3 (-2.5 to 7.9) |
| 30-day rate (any event) | 14 (36) | 13 (35) | -1 (-22.3 to 20.8) |
| 30-day rate (clinically meaningful event) | 0 (0) | 1 (3) | 3 (-2.5 to 7.9) |
| **Dizziness** | | | |
| 7-day rate (any event) | 7 (18) | 6 (16) | -2 (-18.6 to 15.2) |
| 7-day rate (clinically meaningful event) | 0 (0) | 1 (3) | 3 (-2.5 to 7.9) |
| 30-day rate (any event) | 8 (21) | 7 (19) | -2 (-19.5 to 16.3) |
| 30-day rate (clinically meaningful event) | 0 (0) | 1 (3) | 3 (-2.5 to 7.9) |
| **Inability to concentrate** | | | |
| 7-day rate (any event) | 6 (15) | 11 (30) | 15 (-4.2 to 32.9) |
| 7-day rate (clinically meaningful event) | 1 (3) | 2 (5) | 3 (-6.0 to 11.7) |
| 30-day rate (any event) | 8 (21) | 11 (30) | 9 (-10.2 to 28.6) |
| 30-day rate (clinically meaningful event) | 1 (3) | 2 (5) | 3 (-6.0 to 11.7) |
| **Difficulty with urination** | | | |
| 7-day rate (any event) | 3 (8) | 3 (8) | 0 (-11.7 to 12.6) |
| 7-day rate (clinically meaningful event) | 1 (3) | 1 (3) | 0 (-7.1 to 7.3) |
| 30-day rate (any event) | 4 (10) | 4 (10) | 0 (-13.3 to 14.4) |
| 30-day rate (clinically meaningful event) | 1 (3) | 1 (3) | 0 (-7.1 to 7.3) |
| **Confusion** | | | |
| 7-day rate (any event) | 2 (5) | 3 (8) | 3 (-8.2 to 14.2) |
| 7-day rate (clinically meaningful event) | 0 (0) | 1 (3) | 3 (-2.5 to 7.9) |
| 30-day rate (any event) | 2 (5) | 3 (8) | 3 (-8.2 to 14.2) |
| 30-day rate (clinically meaningful event) | 0 (0) | 1 (3) | 3 (-2.5 to 7.9) |

Data are n (%). CME = clinically meaningful event; CI = confidence interval.

© 2022 Do U et al. *JAMA Network Open*.

A clinically meaningful event is any event with a rating of severe or very severe for all symptoms except confusion, where CME is indicated by a rating of moderate severe or very severe[12].
[a] Between-group difference indicates difference in percentages.

© 2022 Do U et al. *JAMA Network Open*.

## eTable 7. Patient-reported postoperative health issues (classified using MedDRA)

| Health issues[a] | Total (N = 76) | Opioid analgesia (n=39) | Opioid-free analgesia (n=37) | Between-group difference (95%CI)[b] |
|---|---|---|---|---|
| Headache | 5 (7) | 4 (10) | 1 (3) | -7 (-3.8 to 18.9) |
| Abdominal distension | 3 (4) | 0 (0) | 3 (8) | 8 (-16.9 to 0.7) |
| Diarrhea | 3 (4) | 1 (3) | 2 (5) | 2 (-11.8 to 6.2) |
| Postoperative wound infection | 2 (3) | 2 (5) | 0 (0) | -5 (-2.2 to 12.5) |
| Cough | 2 (3) | 1 (3) | 1 (3) | 0 (-7.6 to 7.3) |
| Urinary retention | 2 (3) | 1 (3) | 1 (3) | 0 (-7.6 to 7.3) |
| Hypertension | 1 (1) | 1 (3) | 0 (0) | -3 (-2.7 to 7.8) |
| Tachycardia | 1 (1) | 1 (3) | 0 (0) | -3 (-2.7 to 7.8) |
| Breast haematoma | 1 (1) | 1 (3) | 0 (0) | -3 (-2.7 to 7.8) |
| Productive cough | 1 (1) | 0 (0) | 1 (3) | 3 (-7.9 to 2.5) |
| Dyspepsia | 1 (1) | 0 (0) | 1 (3) | 3 (-7.9 to 2.5) |
| Ecchymosis | 1 (1) | 1 (3) | 0 (0) | -3 (-2.7 to 7.8) |
| Hypoesthesia | 1 (1) | 1 (3) | 0 (0) | -3 (-2.7 to 7.8) |
| Neuralgia | 1 (1) | 1 (3) | 0 (0) | -3 (-2.7 to 7.8) |
| Oropharyngeal pain | 1 (1) | 0 (0) | 1 (3) | 3 (-7.9 to 2.5) |
| Penile swelling | 1 (1) | 0 (0) | 1 (3) | 3 (-7.9 to 2.5) |
| Testicular swelling | 1 (1) | 0 (0) | 1 (3) | 3 (-7.9 to 2.5) |
| Seroma | 1 (1) | 0 (0) | 1 (3) | 3 (-7.9 to 2.5) |
| Urinary tract infection | 1 (1) | 0 (0) | 1 (3) | 3 (-7.9 to 2.5) |
| Cystitis | 1 (1) | 0 (0) | 1 (3) | 3 (-7.9 to 2.5) |
| Peripheral neuropathy | 1 (1) | 0 (0) | 1 (3) | 3 (-7.9 to 2.5) |

Data are n (%).

[a] Data was obtained from spontaneous patient reporting (Patients were asked, “Did you have any significant medical problem related or unrelated to your surgery since the last study assessment?” at every postoperative time-point) and from data reported by clinicians in electronic medical records.

[b] Between-group difference represents difference in percentages.

© 2022 Do U et al. *JAMA Network Open*.

**eReferences**


1. Moore RA, Derry S, Aldington D, Wiffen PJ. Single dose oral analgesics for acute postoperative pain in adults - an overview of Cochrane reviews. *Cochrane Database Syst Rev*. 2015;2015(9):Cd008659. doi:10.1002/14651858.CD008659.pub3
2. Gaskell H, Derry S, Wiffen PJ, Moore RA. Single dose oral ketoprofen or dexketoprofen for acute postoperative pain in adults. *Cochrane Database Syst Rev*. 2017;5(5):Cd007355. doi:10.1002/14651858.CD007355.pub3
3. Moore A, Derry S, Eccleston C, Kalso E. Expect analgesic failure; pursue analgesic success. *Bmj*. 2013;346:f2690. doi:10.1136/bmj.f2690
4. Suffeda A, Meissner W, Rosendahl J, Guntinas-Lichius O. Influence of depression, catastrophizing, anxiety, and resilience on postoperative pain at the first day after otolaryngological surgery: A prospective single center cohort observational study. *Medicine (Baltimore)*. 2016;95(28):e4256. doi:10.1097/md.0000000000004256
5. Sullivan MJL, Bishop SR, Pivik J. The pain catastrophizing scale: development and validation. *Psychol Assess*. 1995;7(4):524-532.
6. Akbik H, Butler SF, Budman SH, Fernandez K, Katz NP, Jamison RN. Validation and clinical application of the Screener and Opioid Assessment for Patients with Pain (SOAPP). *J Pain Symptom Manage*. 2006;32(3):287-93. doi:10.1016/j.jpainsymman.2006.03.010
7. Keller S, Bann CM, Dodd SL, Schein J, Mendoza TR, Cleeland CS. Validity of the brief pain inventory for use in documenting the outcomes of patients with noncancer pain. *Clin J Pain*. 2004;20(5):309-18. doi:10.1097/00002508-200409000-00005
8. Hah J, Mackey SC, Schmidt P, et al. Effect of Perioperative Gabapentin on Postoperative Pain Resolution and Opioid Cessation in a Mixed Surgical Cohort: A Randomized Clinical Trial. *JAMA Surg*. 2018;153(4):303-311. doi:10.1001/jamasurg.2017.4915
9. Krebs EE, Gravely A, Nugent S, et al. Effect of Opioid vs Nonopioid Medications on Pain-Related Function in Patients With Chronic Back Pain or Hip or Knee Osteoarthritis Pain: The SPACE Randomized Clinical Trial. *Jama*. 2018;319(9):872-882. doi:10.1001/jama.2018.0899
10. Abola RE, Bennett-Guerrero E, Kent ML, et al. American Society for Enhanced Recovery and Perioperative Quality Initiative Joint Consensus Statement on Patient-Reported Outcomes in an Enhanced Recovery Pathway. *Anesth Analg*. 2018;126(6):1874-1882. doi:10.1213/ane.0000000000002758
11. van der Meij E, Anema JR, Huirne JAF, Terwee CB. Using PROMIS for measuring recovery after abdominal surgery: a pilot study. *BMC Health Serv Res*. 2018;18(1):128. doi:10.1186/s12913-018-2929-9
12. Chan KS, Chen WH, Gan TJ, et al. Development and validation of a composite score based on clinically meaningful events for the opioid-related symptom distress scale. *Qual Life Res*. 2009;18(10):1331-40. doi:10.1007/s11136-009-9547-2
13. Knisely JS, Wunsch MJ, Cropsey KL, Campbell ED. Prescription Opioid Misuse Index: a brief questionnaire to assess misuse. *J Subst Abuse Treat*. 2008;35(4):380-6. doi:10.1016/j.jsat.2008.02.001
14. Slankamenac K, Graf R, Barkun J, Puhan MA, Clavien PA. The comprehensive complication index: a novel continuous scale to measure surgical morbidity. *Ann Surg*. 2013;258(1):1-7. doi:10.1097/SLA.0b013e318296c732
15. MedDRA. Medical Dictionary for Regulatory Activities. Accessed March 1, 2021. https://www.meddra.org/
16. Canada's Low-Risk Alcohol Drinking Guidelines. 2018. Accessed March 21, 2021. https://www.ccsa.ca/canadas-low-risk-alcohol-drinking-guidelines-brochure


© 2022 Do U et al. *JAMA Network Open*.

17. Ousmen A, Touraine C, Deliu N, et al. Distribution- and anchor-based methods to determine the minimally important difference on patient-reported outcome questionnaires in oncology: a structured review. *Health Qual Life Outcomes*. 2018;16(1):228. doi:10.1186/s12955-018-1055-z
18. Myles PS, Myles DB, Galagher W, et al. Measuring acute postoperative pain using the visual analog scale: the minimal clinically important difference and patient acceptable symptom state. *Br J Anaesth*. 2017;118(3):424-429. doi:10.1093/bja/aew466
19. Dworkin RH, Turk DC, Wyrwich KW, et al. Interpreting the clinical importance of treatment outcomes in chronic pain clinical trials: IMMPACT recommendations. *J Pain*. 2008;9(2):105-21. doi:10.1016/j.jpain.2007.09.005

© 2022 Do U et al. *JAMA Network Open*.
