# Supplementary material for: Feasibility of Prospectively Comparing Opioid Analgesia With Opioid-Free Analgesia After Outpatient General Surgery: A Pilot Randomized Clinical Trial
Source: JAMA Netw Open. 2022 Jul 18;5(7):e2221430. doi: 10.1001/jamanetworkopen.2022.21430 (PMC9294998; doi:10.1001/jamanetworkopen.2022.21430)
Supplement: Supplement 3. — Data Sharing Statement [file jamanetwopen-e2221430-s003.pdf]

Data Sharing Statement

Do. Feasibility of Prospectively Comparing Opioid Analgesia With Opioid-Free Analgesia After Outpatient General Surgery. *JAMA Netw Open*. Published July 18, 2022. doi:10.1001/jamanetworkopen.2022.21430

Data
Data available: No
